# Biosynthesis of the Isocoumarin Derivatives Fusamarins is Mediated by the PKS8 Gene Cluster in *Fusarium*

**DOI:** 10.1002/cbic.202200342

**Published:** 2022-10-18

**Authors:** Anna K. Atanasoff‐Kardjalieff, Bernhard Seidl, Katharina Steinert, Constantin G. Daniliuc, Rainer Schuhmacher, Hans‐Ulrich Humpf, Svetlana Kalinina, Lena Studt‐Reinhold

**Affiliations:** ^1^ Institute of Microbial Genetics Department of Applied Genetics and Cell Biology University of Natural Resources and Life Sciences Vienna Konrad-Lorenz Strasse 24 3430 Tulln an der Donau Austria; ^2^ Institute of Bioanalytics and Agro-Metabolomics Department of Agrobiotechnology (IFA-Tulln) University of Natural Resources and Life Sciences Vienna Konrad-Lorenz Strasse 20 3430 Tulln an der Donau Austria; ^3^ Institute of Food Chemistry Westfälische Wilhelms-Universität Münster Corrensstraße 45 48149 Münster Germany; ^4^ Organisch-Chemisches Institut Westfälische Wilhelms-Universität Münster Corrensstraße 40 48149 Münster Germany

**Keywords:** dihydroisocoumarins, *Fusarium fujikuroi* species complex (FFSC), heterochromatin, polyketides, secondary metabolites

## Abstract

*Fusarium mangiferae* causes the mango malformation disease (MMD) on young mango trees and seedlings resulting in economically significant crop losses. In addition, *F. mangiferae* produces a vast array of secondary metabolites (SMs), including mycotoxins that may contaminate the harvest. Their production is tightly regulated at the transcriptional level. Here, we show that lack of the H3 K9‐specific histone methyltransferase, FmKmt1, influences the expression of the *F. mangiferae* polyketide synthase (PKS) 8 (*FmPKS8*), a so far cryptic PKS. By a combination of reverse genetics, untargeted metabolomics, bioinformatics and chemical analyses including structural elucidation, we determined the FmPKS8 biosynthetic gene cluster (BGC) and linked its activity to the production of fusamarins (FMN), which can be structurally classified as dihydroisocoumarins. Functional characterization of the four *FMN* cluster genes shed light on the biosynthetic pathway. Cytotoxicity assays revealed moderate toxicities with IC_50_ values between 1 and 50 μM depending on the compound.

## Graphical abstract

**Fusaria** are plant‐pathogenic fungi infecting several staple crops, and accumulating often toxic compounds during infection and hence threaten food security worldwide. The full chemical potential of these notorious pathogens is still elusive. Here, we characterized the previously cryptic PKS8 gene cluster and show that four genes are responsible for the biosynthesis of the dihydroisocoumarin derivates fusamarins with moderate cytotoxic effects.

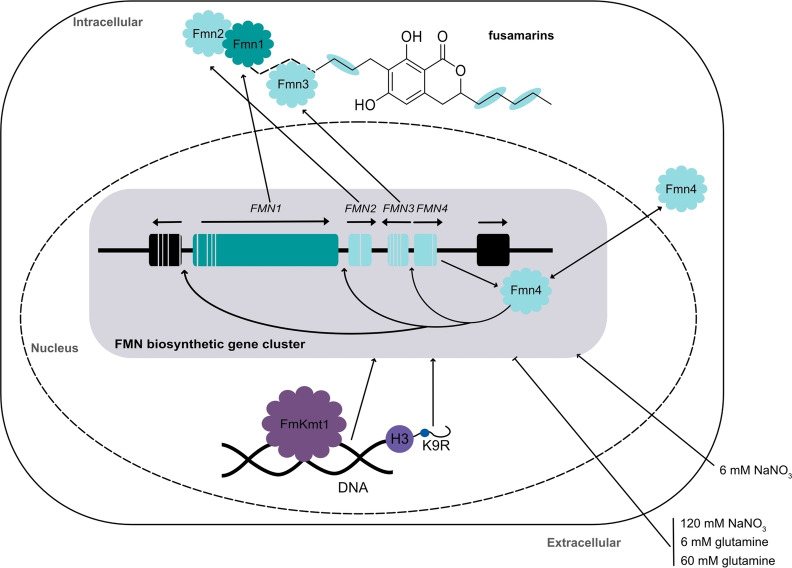

## Introduction

The genus *Fusarium* comprises at least 300 phylogenetically distinct species. Many of them are notorious plant‐pathogens, infecting various important crop plants, and as such are detrimental for modern agri‐ and horticulture.^[2]^ Next to infecting host plants, fusaria are well known to produce small molecular‐weight compounds, also referred to as secondary metabolites (SMs), often exhibiting a diverse array of biological activities. This includes potent mycotoxins that accumulate in the host during infection and may cause severe intoxications upon consumption of the derived food and feed.^[3]^ Several mycotoxigenic *Fusarium* species have been characterized over the past decades, while others are still not well described. One example of the latter is the plant‐pathogenic fungus *Fusarium mangiferae*, which belongs to the *Fusarium fujikuroi* species complex (FFSC).^[4]^

*F. mangiferae* is the causal agent of the mango malformation disease (MMD) on mango (*Mangifera indica*) seedlings and young trees in the southern hemisphere. MMD caused by *F. mangiferae* can lead either to vegetative or floral malformation resulting in enormous yield losses or even the complete forfeiture of the harvest.^[5]^ Similar to other fusaria, the *F. mangiferae* genome has the genetic potential to produce a broad spectrum of SMs. Overall, *F. mangiferae* harbors 52 SM key enzyme‐encoding genes, potentially giving rise to an array of structurally diverse compounds.^[4]^ Thus far, only 19 of these genes have been linked to their respective product either experimentally or *via* comparative genomic analysis, and even less is known regarding the biosynthesis of the respective SMs during axenic or *in planta* growth.^[4]^ This may also include toxic compounds, which are currently not considered for regular monitoring by legal authorities. In this regard, understanding SM gene regulation and decrypting the chemical potential of notorious plant‐pathogens is crucial.

Generally, SMs are synthesized from only a few building blocks originating from primary metabolism and belong to one or a combination of two of the four chemical groups depending on the involved SM key enzyme(s): polyketides, non‐ribosomal peptides, terpenoids and indole alkaloids that are synthesized by polyketide synthases (PKSs), non‐ribosomal peptide synthetases (NRPSs) and terpene synthases (TSs) respectively.^[6]^ Among these, polyketides constitute the most abundant chemical group comprising an enormous structural diversity and a wide range of biological activities. Fungal polyketides are generally synthesized by type I PKSs that constitute multi‐domain enzymes and orchestrate distinct numbers of decarboxylative Claisen condensations using acetyl‐CoA as starter unit, though other starter units have also been identified,^[7]^ and malonyl‐CoA as extending units in the polyketide assembly. The overall domain structure of the PKS is fundamental for the structural properties of the resulting polyketide. Non‐reducing (NR)‐PKS contain the minimal domain configuration required for polyketide assembly i. e., an acyltransferase (AT) domain, involved in recognition of the building blocks, a ketosynthase (KS) domain that catalyzes a certain number of successive Claisen condensations, and an acyl carrier protein (ACP) that serves as a covalent binding site for the intermediate formed during synthesis. Partially (PR‐) and highly reducing (HR)‐PKS may harbor additional domains involved in the step‐wise reduction of the keto groups formed during the elongation process. Those include a ketoreductase (KR), a dehydratase (DH) and an enoyl reductase (ER) domain, all of which are selectively utilized during chain elongation. Next to this, an intrinsic C‐methyltransferase (C‐Met) domain that catalyzes the transfer of methyl groups from S‐adenosylmethionine (SAM) to the β‐carbon of the growing polyketide chain may be part of the PKS (mainly but not exclusively found in HR‐PKSs). Since PKSs are almost universally iterative, the programming of HR‐PKS is particularly complex as C‐methylation, reduction, dehydration and enoyl reduction reactions may (or may not) occur during each round of elongation.^[8]^ Finally, the polyketide is released from the PKS. This is often facilitated either by a release domain, such as a thioesterase (TE)‐ or a carnitine O‐acyltransferase (cAT)‐domain, located at the C‐terminal end of the PKS. However, the product may also be offloaded by discrete proteins or by a spontaneous release. Thus, the offloading mechanism shapes the chemical structure of the resulting polyketide.[^9^, ^10^] The resulting polyketide may then be further modified by so‐called tailoring‐enzymes^[11]^ that install additional functional groups (e. g., hydroxyl or methyl groups), and are generally encoded within the same biosynthetic gene cluster (BGC).^[12]^ Next to these biosynthetic proteins, BGCs often comprise genes involved in the transcriptional regulation of the cluster genes or the transport of the SM formed.^[13]^ This spatial proximity of the genes involved in the biosynthesis, regulation and transport of single SMs is thought to facilitate their co‐expression upon receiving appropriate stimuli.

One decisive layer in transcriptional regulation is mediated by the chromatin structure. In eukaryotes, genomic DNA wraps around histone protein octamers to form nucleosome chains which finally are condensed to chromatin. The histones constitute a platform for various posttranslational modifications (PTMs) or histone marks, which in turn influence the accessibility of the DNA for the transcriptional machinery, and as such transcription of the underlying genes. The chromatin structure, as determined by changes in histone marks, plays a key role in the expression of BGCs in several filamentous fungi, including *Fusarium* spp.^[14]^ One such histone mark is trimethylation on histone H3 lysine 9 (H3K9me3). H3K9me3 is in general associated with heterochromatin formation and as such with gene silencing as shown for the fission yeast *Schizosaccharomyces pombe* (SpClr4)^[15]^ or *Neurospora crassa* (NcDIM5).^[16]^ H3K9me3 is established by FmKmt1 in *F. mangiferae*.^[17]^ The role of H3K9me3 in fungal development and virulence is diverse, not only in the genus *Fusarium* but in the fungal kingdom in general. While in *F. mangiferae* and *Fusarium verticillioides* loss of Kmt1 only had subtle influence on growth and development,^[17]^ more severe developmental phenotypes were observed for *Epichloë festucae* and *Zymoseptoria tritici*.^[18]^ Notably, not much is known about the role of H3K9me3 in SM‐gene regulation in filamentous fungi thus far. Previously, we have shown that FmKmt1 is crucial for wild type‐like expression of selected BGCs in *F. mangifera*e i. e., FmNRPS22 and FmPKS40 involved in beauvericin and fusapyrone biosynthesis, respectively, as well as the cryptic FmPKS8 BGC.^[17]^

In this study, we revealed that FmPKS8 is involved in the biosynthesis of a family of dihydroisocoumarins, designated fusamarins (FMN), which were firstly reported over 50 years ago.^[19]^ Yet, nothing was known about the genes involved in their biosynthesis or their regulation. Here, we show that in *F. mangiferae*, FMN biosynthesis is facilitated by the formerly cryptic FmPKS8 BGC. Expression of the FmPKS8 BGC depends on the functional H3K9‐specific histone methyltransferase, FmKmt1. Next to *FmPKS8* (*FmFMN1*), three additional genes belong to this BGC, i. e., *FmFMN2*−*FmFMN4*, as determined by bioinformatics, reverse genetics, molecular and chemical characterization. Interestingly, the PKS8 BGC is abrogated in all thus far sequenced isolates of the closely related *F. fujikuroi* and *Fusarium proliferatum* strains but conserved in *F. verticillioides* and some other fusaria mainly found within the FFSC. Cytotoxicity assays with FMN revealed moderate toxicities depending on the compound tested.

## Results and Discussion

### Identification of putative FmPKS8‐derived metabolites from *F. mangiferae*

We have previously shown that the cryptic *FmPKS8* is expressed in low nitrogen conditions, and that its expression depends on a functional ortholog of FmKmt1.^[17]^ To identify possible FmPKS8‐derived products, an untargeted metabolomics approach was used. For this, the *F. mangiferae* wild‐type strain MRC7560 (from now on referred to as FmWT), was grown simultaneously with the previously generated *FmPKS8* deletion strain, Δ*fmPKS8*,^[17]^ under *FmPKS8*‐inducing conditions i. e., synthetic liquid ICI medium using 6 mM sodium nitrate (NaNO_3_) as sole nitrogen source. Culture filtrates were subsequently subjected to high performance liquid chromatography‐high resolution mass spectrometry (HPLC‐HRMS). To facilitate this approach, we included another strain, Δ*fmppt1*, which lacks the 4′‐phosphopantetheinyl transferase (Ppt) that is used for binding biosynthetic intermediates to the ACP domain and is therefore crucial for PKS and NRPS function.^[20]^ Consequently, loss of *FmPPT1* is expected to lead to the abolishment of all PKS‐ and NRPS‐derived compounds in *F. mangiferae*.

For the comparative, untargeted metabolomics approach, aiming to identify the gene products of the PKS8 BGC at the metabolite level, the culture supernatant filtrates of the selected strains were analyzed in a dilute‐and‐shoot approach using reversed‐phase HPLC coupled to HRMS. The pure cultivation media and solvents used for sample preparation (ultra‐pure water, methanol and acetonitrile) were measured in the same way as the biological samples. Any chromatographic features, which were found in the media blank and/or solvent blank samples as well as in in the biological samples were assigned as background and discarded from further analysis. Thereafter, the HPLC‐HRMS data were specifically searched for features that were exclusive to the wild type when compared to the respective knockout strains, as would be expected for gene products from wild type versus gene knockout strains.

Overall, the untargeted, differential metabolite profile analysis identified 18 compounds, which were absent from Δ*fmPKS8* and Δ*fmPPT1* but highly abundant in culture filtrates of FmWT (Figure S1/S2). The metabolites and their putative molecular formulas, which were calculated from the accurate mass, as well as retention times and maximum peak areas are listed in Table S1. Of the 18 metabolites found exclusively in the FmWT, eight metabolites were selected as target compounds, based on their elemental composition, presumably synthesized by FmPKS8 and additional cluster genes (Table 1). In addition, a targeted search was made for other possible variants with the determined chain length varying only in the number of hydrogen and oxygen present (i. e., C_18_H_20_O_4_ molecular formulas C_18_H_20+n_O_4+m_ (n=0,2,4,6,8,10; m=0,1) and thereby C_18_H_28_O_5_ at retention time 25.42 min was found as an additional putative FmPKS8 product. Table 1 lists all nine identified target metabolites putatively being derived from the FmPKS8 BGC (from now on referred to as compound **1**–**9**).

**Table 1:** List of finally selected FmPKS8 target compounds sorted by retention time.

ID	Formula	Retention time [min]	[M+H]^+^ *m*/*z*	[M−H]^−^ *m*/*z*
1	C_18_H_22_O_4_	23.43	303.1591	301.1445
2	C_18_H_24_O_5_	23.44	321.1697	319.1551
3	C_18_H_26_O_5_	24.40	323.1853	321.1707
4	C_18_H_28_O_5_	25.42	325.2010	323.1864
5	C_18_H_20_O_4_	27.52	301.1434	299.1289
6	C_18_H_22_O_4_	28.30	303.1591	301.1445
7	C_18_H_24_O_4_	29.25	305.1747	303.1602
8	C_18_H_26_O_4_	29.93	307.1904	305.1758
9	C_18_H_22_O_4_	30.72	303.1591	301.1445

**Figure 1:**
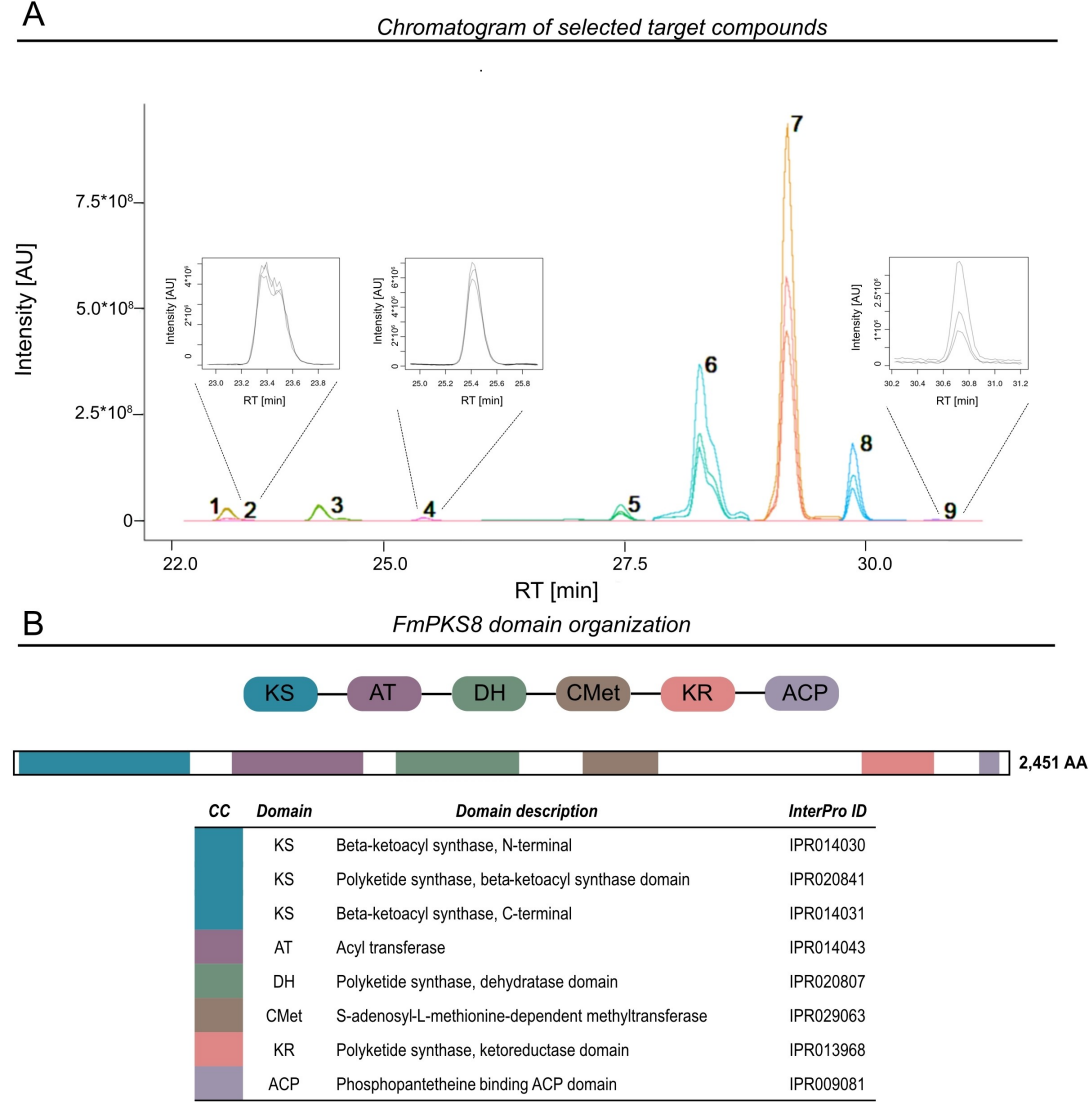
Untargeted metabolomics approach revealed nine possible target compounds. (A) Extracted ion chromatograms (EICs) of the respectively most abundant ion species [M+H]^+^ of all nine FmPKS8‐associated metabolite features (**1**–**9**), found in the HPLC‐HRMS data. Signal intensity is given in arbitrary units (AU).The numbers given next to the chromatographic peaks correspond to the target numbers of those from Table 1. The EICs show signals only for the three biological replicates of the wild‐type samples but no signals for the knock out strains (Figure S2). (B) Schematic and detailed domain organization of FmPKS8. FmPKS8 harbors typical domains of a highly reducing (HR)‐PKS, including a dehydratase (DH), intrinsic S‐adenosyl‐methionine (SAM)‐dependent methyltransferase (CMet) and keto reductase (KR) domain. The detailed domain structure was determined using InterPro^[1]^ database for depiction. CC denotes color code.

The predicted structures revealed similar structural properties that vary only in the number of hydrogen and oxygen present in the chemical formula (Figure 1A) and are in agreement with the domain architecture of FmPKS8 that comprises typical features of a HR‐PKS (Figure 1B).

### Formation of target compounds depends on the H3K9 methyltransferase FmKmt1 and are repressed by nitrogen

**Figure 2:**
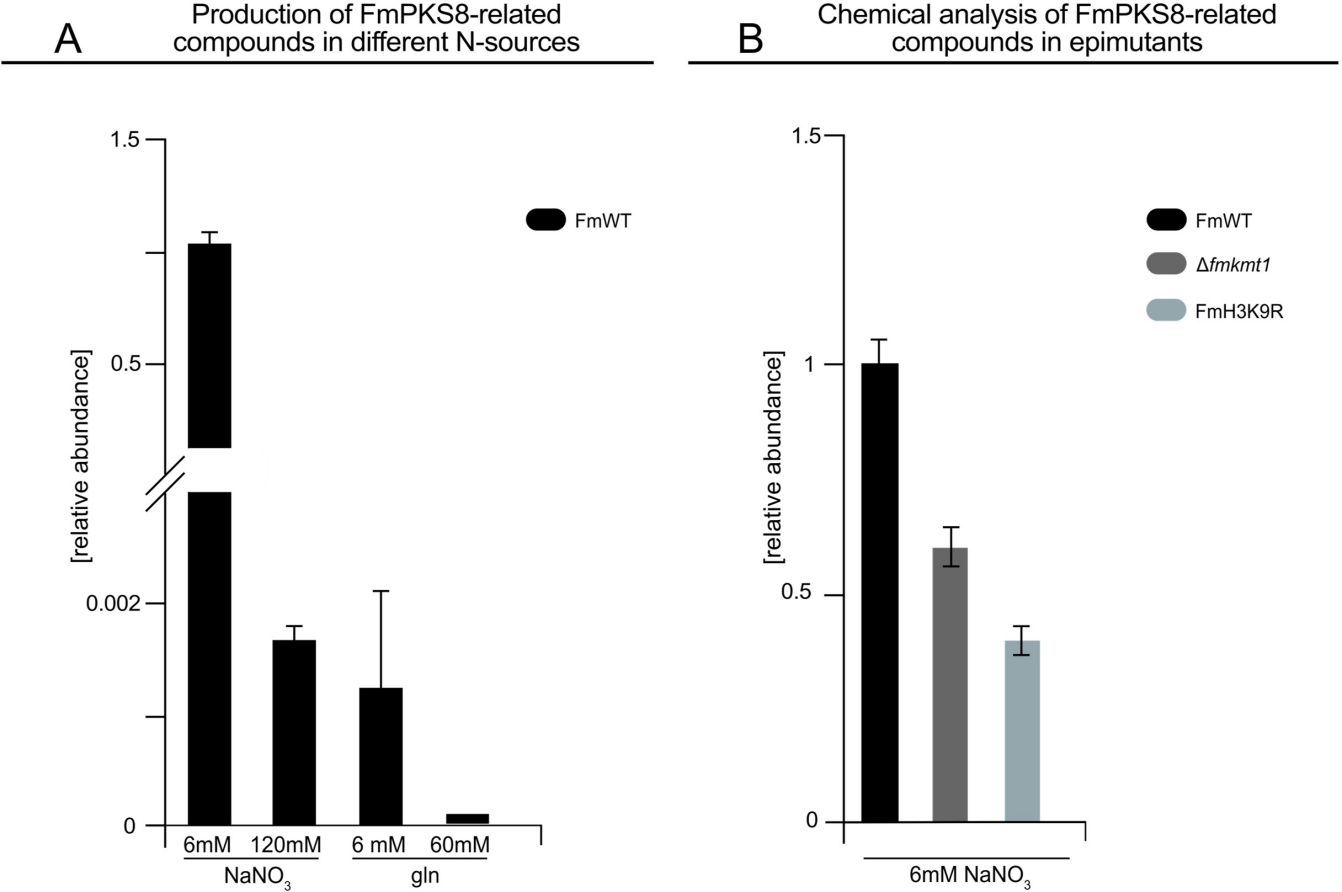
Biosynthesis of identified target compounds (**1**–**9**) depends on the nitrogen source and on the H3K9‐specific histone methyltransferase FmKmt1. (A) Biosynthesis of the identified target compounds in dependence of the nitrogen source. *Fusarium mangiferae* wild type strain (FmWT) was grown for seven days in synthetic ICI medium containing either 6 or 120 mM NaNO_3_, or 6 or 60 mM glutamine (gln), and culture filtrates were directly applied for HPLC‐HRMS. (B) Biosynthesis of the identified target compounds in dependence of H3K9me3. FmWT, Δ*fmkmt1* and FmH3K9R were grown in *FmPKS8*‐inducing conditions. Culture filtrates were subsequently applied for HPLC‐HRMS. Experiments were performed in triplicates. For comparison of the overall production levels, relative peak areas of the target compounds were summed up and related to the respective biomass. Mean values and standard deviations are shown. Biosynthesis of FmWT was arbitrarily set to 1.

We have previously shown that *FmPKS8* expression greatly depends on the nitrogen source and concentration i. e., *FmPKS8* expression is specifically induced in liquid ICI medium with 6 mM NaNO_3_ as sole nitrogen source (*FmPKS8*‐inducing conditions). While only low or no transcripts are detectable when either 6 or 60 mM glutamine or 120 mM NaNO_3_ is used.^[17]^ To further validate the FmPKS8 origin of compound **1**–**9**, FmWT was grown for seven days under these different nitrogen supply conditions, and culture filtrates were subsequently analyzed by targeted chemical analysis *via* HPLC‐HRMS. As expected, and in line with our previous results, the target compounds **1**–**9** were abundant in *FmPKS8*‐inducing conditions, whereas only low amounts were produced in presence of 6 and 60 mM glutamine, and 120 mM NaNO_3_ (Figure 2A).

Expression of many SM genes depends on the available nitrogen sources,^[21]^ and regulation of at least some of these genes is influenced by the GATA‐type transcription factor (TF) AreA in the closely related rice pathogen *F. fujikuroi*.^[22]^ Notably, the affected metabolites include also nitrogen‐free SMs such as gibberellins, phytohormones which are strictly AreA‐dependent.^[23]^ AreA facilitates the activation of genes involved in the utilization of non‐preferred nitrogen sources, including NaNO_3_.[^23^, ^24^] Thus, mutants deficient for *AREA* are not able to grow on medium supplemented with NaNO_3_ as sole nitrogen source,^[24]^ thereby impeding to analyze the involvement of AreA in regulating *FmPKS8* expression.

Next, FmWT and Δ*fmkmt1* strains were grown for seven days under *FmPKS8‐*inducing conditions, as we have previously shown that *FmPKS8* expression is negatively affected by the loss of FmKmt1 involved in the formation of the H3K9me3 mark.^[17]^ To unequivocally connect *FmPKS8* expression to H3K9me3, a strain in which lysine 9 of histone 3 (H3K9) was exchanged for an arginine (FmH3K9R), thereby preventing the modification of K9, including methylation but also acetylation.^[17]^ Not surprisingly, this strain had a more severe phenotypic appearance as compared to Δ*fmkmt1* (Figure S3). Subsequent chemical analysis revealed high abundance of the target compounds **1**–**9** in cultures of FmWT, while their biosynthesis was significantly decreased in Δ*fmkmt1* and FmH3K9R (Figure 2B) as expected. This finding is in line with the gene expression data (Figure S4). To compare production levels among selected fungal strains and culture conditions, the relative abundances of the single [M+H]^+^ ion peak areas of the selected target compounds **1**–**9** were summed up for better clarity. This was possible since the relative abundance distribution of the nine targets always remained constant for all strains.

Thus, we identified overall nine target compounds (**1**–**9**) with similar structural properties. In each case, production levels were in agreement with gene expression data thereby underlining a possible FmPKS8 origin of **1**–**9**.

### Delineation of the FmPKS8 gene cluster

To identify additional genes belonging to the FmPKS8 BGC and to determine the cluster borders, the expression levels of genes in close proximity to *FmPKS8* were analyzed by reverse‐transcriptase quantitative polymerase chain reaction (RT‐qPCR).

**Figure 3:**
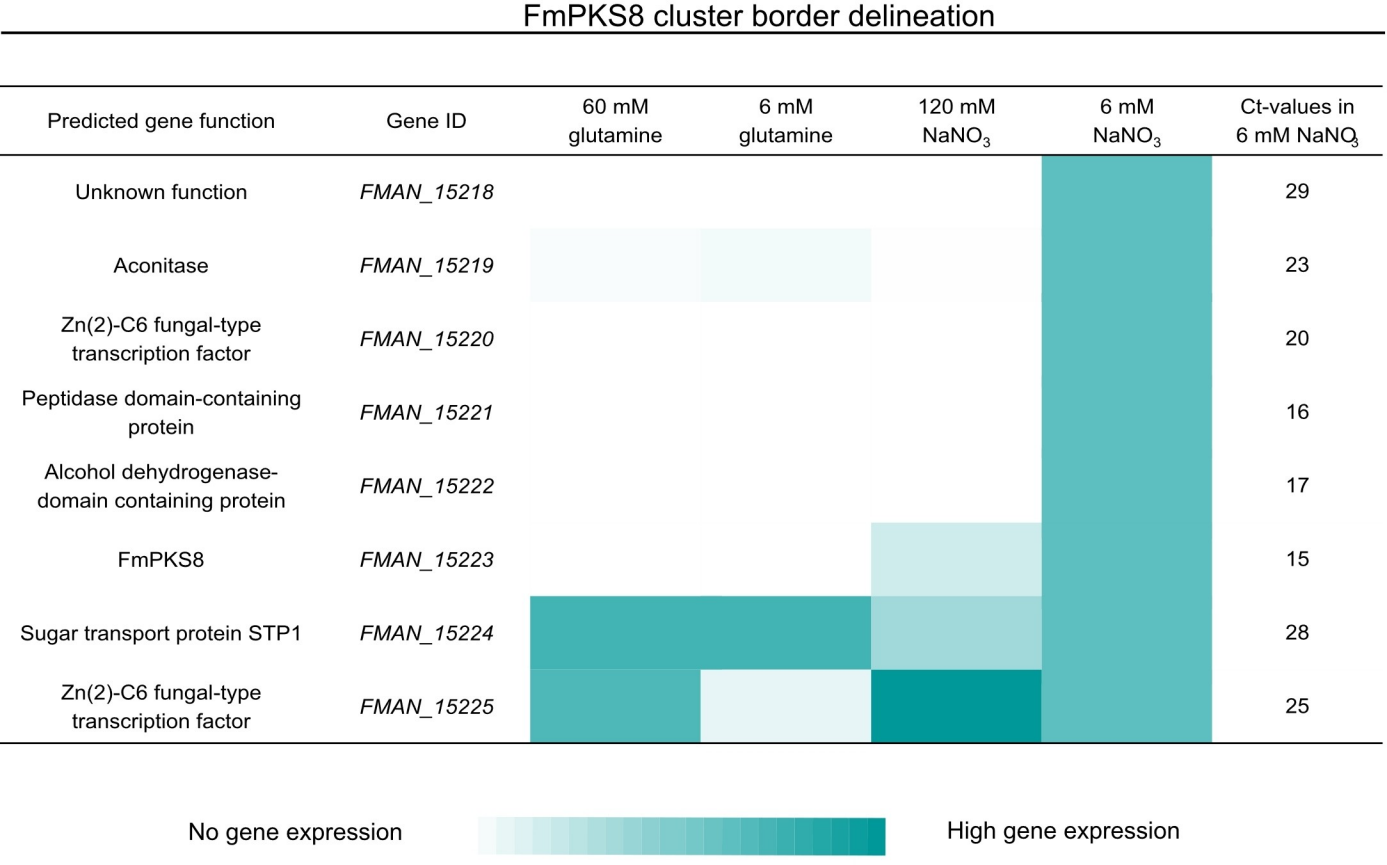
Cluster border delineation of the FmPKS8 BGC. Gene expression (normalized values) in form of a heat map of FmPKS8 and adjacent genes to elucidate FmPKS8 cluster borders. For this, *F. mangiferae* wild type (FmWT) was grown in different nitrogen sources and conditions. Mycelium was harvested 3 days post inoculation (dpi), followed by RNA extraction and cDNA synthesis for RT‐qPCR analysis. Primers are listed in Supporting Information Table S2.

For this, FmWT was cultivated under *FmPKS8*‐inducing as well as ‐repressing conditions i. e., 120 mM NaNO_3_, 6 and 60 mM glutamine respectively. Next to *FmPKS8* (*FMAN*_*15223*), co‐expression of seven other genes was detected under *FmPKS8*‐inducing conditions i. e., *FMAN*_*15218*‐*FMAN*_*15225* (Figure 3).

While expression of the genes putatively encoding a sugar transport protein (*FMAN*_*15224*) and a Zn(2)‐C6 fungal‐type TF (*FMAN*_*15225*) was also detected and even increased under *FmPKS8*‐repressing conditions, expression of *FMAN*_*15218*‐*FMAN*_*15223* was restricted to *FmPKS8*‐inducing conditions. Thus, the putative sugar transporting protein (*FMAN*_*15224*) and the nearby Zn(2)‐C6 fungal‐type TF (*FMAN*_*15225*) are likely not part of the FmPKS8 BGC. Moreover, though expression was highest in *FmPKS8*‐inducing conditions, for a protein with unknown function (*FMAN*_*15218*) transcript levels were almost non‐detectable under these conditions, suggesting that the protein with an unknown function (*FMAN*_*15218*) is also not included in the FmPKS8 BGC. Thus, the expression data suggest that the FmPKS8 BGC spans five genes i. e., *FMAN*_*15219*‐*FMAN*_*15223*.

**Figure 4:**
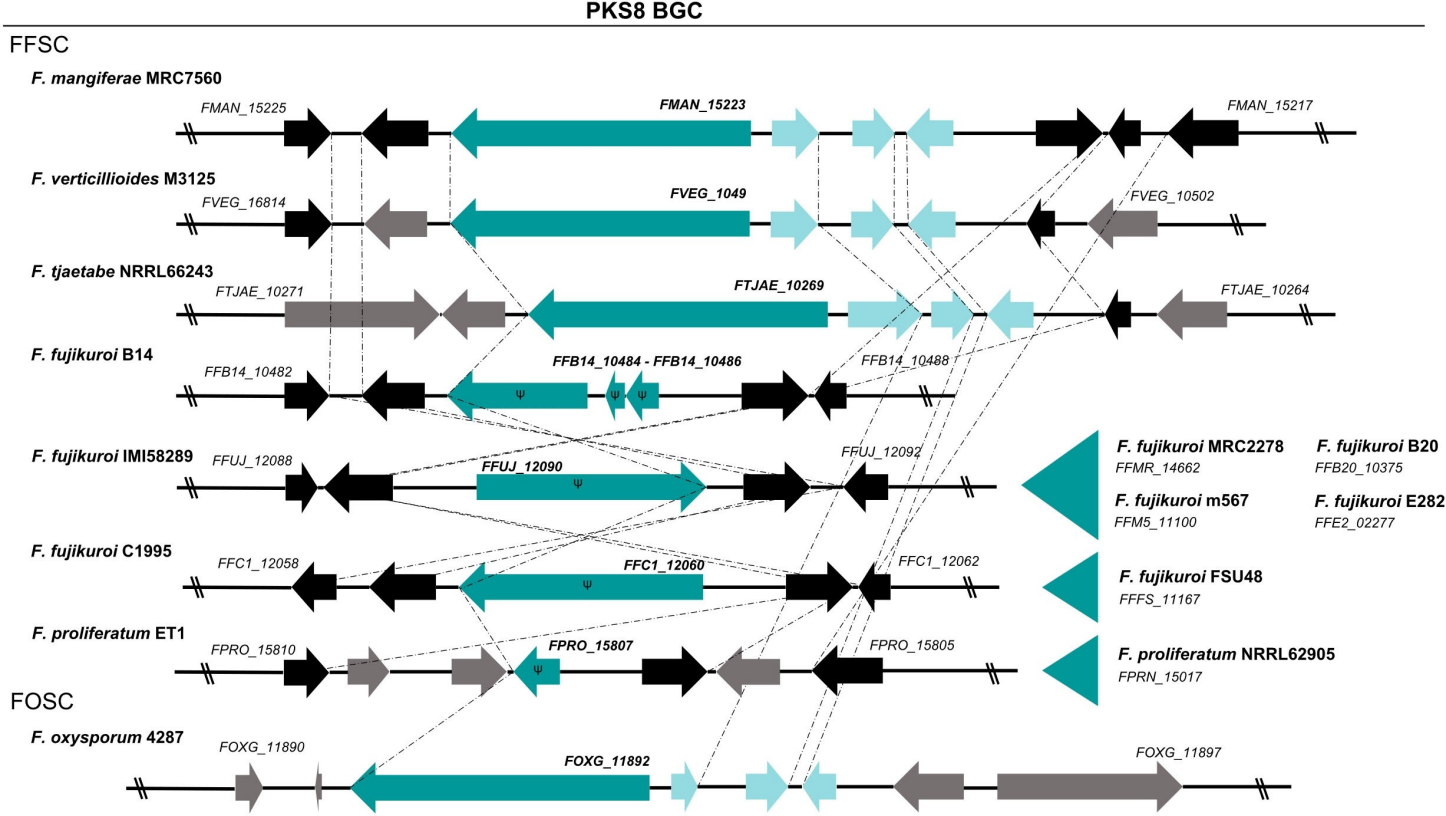
Comparative genomic analysis of PKS8 and putative cluster genes in the genus *Fusarium*. Optical maps of putative PKS8 BGCs in different fusaria, including species from the FFSC and *F. oxysporum*. Turquoise arrows indicate the PKS8 key enzyme‐encoding gene, while light turquoise arrows show putative cluster genes. The black arrows represent genes that are orthologs from other species and grey arrows denote genes which do not have an ortholog within the species. Ψ designates a pseudogene.

To further delineate the cluster borders and to get more insight into the presence of an orthologous FmPKS8 BGC in other fusaria, the FmPKS8 protein sequence was compared with publicly available *Fusarium* sequence data by using pBlast^[25]^ and/or QuartetS.^[26]^ PKS8 orthologs were detected mostly in members of the FFSC, but also in some *F. oxysporum* isolates, in members of the *Fusarium nisikadoi* species complex (FNSC) as well as in some other isolates such as *Fusarium austroafricanicum* and *Fusarium babinda*. Genome sequences were accessible for some members of the FFSC, including several *F. fujikuroi* and *F. proliferatum* isolates, *F. verticillioides* and *F. tjaetabe* as well as *F. oxysporum* (Figure 4). Notably, the sequenced *F. fujikuroi strains* i. e., B20, B14, C1995, E282, FUS48, m567, MRC2276 and IMI58289,^[27]^ do not harbor a functional PKS8 as the KS domain, that is involved in polymerization of the building blocks and as such vital for chain elongation,^[28]^ is absent from all FfPKS8 orthologs (Figure 4). Moreover, none of the other putative cluster genes are present in the *F. fujikuroi* genomes. Likewise, in both sequenced *F. proliferatum* strains i. e., ET1 and NRRL62905, only remnants of the PKS8 key enzyme‐encoding gene and none of the putative cluster genes are present, suggesting that the cluster is abrogated in these closely related species. Contrary to this, *F. verticillioides* M3125, *F. oxysporum* 4287 and *F. tjaetabe* NRRL66243 harbor an ortholog of *PKS8*. While collinearity was observed for *FMAN*_*15223*‐*FMAN*_*15220*, orthologs of the putative cluster gene *FMAN*_*15219*, encoding a putative aconitase, were missing from those strains. Moreover, orthologs of *FMAN*_*15224* encoding a putative sugar transporter are also absent from the *F. verticillioides*, *F. oxysporum* and *F. tjaetabe* genomes. Thus, next to the PKS8, only three additional genes are commonly present in the different *Fusarium* genomes. These encode a putative alcohol dehydrogenase domain‐containing protein predicted to own *trans*‐ER function (*FMAN*_*15222*), a putative peptidase‐containing protein with an α/β hydrolase fold (*FMAN*_*15221*) and a Zn(2)‐C6 fungal‐type TF (*FMAN*_*15220*).

To evaluate whether the four genes, *FMAN*_*15223*‐*FMAN*_*15220*, are sufficient for the biosynthesis of the target compounds **1**–**9**, the *F. verticillioides* M3125 strain (from now on referred to as FvWT), harboring four (*FVEG*_*10497*‐*FVEG*_*10500*) instead of the five co‐expressed PKS8 cluster genes in *F. mangiferae* (Figure 4), was cultivated under *FmPKS8‐*inducing conditions. Subsequently, expression of *FvPKS8* was analyzed four to seven days post inoculation (dpi) by semi‐quantitative PCR (Figure S5). Semi‐quantitative PCR indeed showed expression of *FvPKS8* four to seven days, though to a lesser extent compared to *FmPKS8*. Next, culture filtrates of FvWT grown for seven days under *PKS8*‐inducing conditions were analyzed by HPLC‐HRMS, to determine whether the target compounds **1**–**9** are also produced in *F. verticillioides*. In accordance with the expression data, the target compounds **1**–**9** were also produced by FvWT though less abundant as compared to FmWT (Figure S6A), thereby indicating that four genes are sufficient for the biosynthesis of **1**–**9**. Subsequent partial gene deletion of *FvPKS8* (*FVEG*_*10497*) further verified the involvement of FvPKS8 in the biosynthesis of **1**–**9** as none of the target compounds was produced in Δ*fvPKS8* (Figure S6B).

Finally, to unequivocally verify FmPKS8 cluster borders, targeted deletion of the putative aconitase‐ (*FMAN*_*15219*) and the putative sugar transporter‐encoding gene (*FMAN*_*15224*), that are located immediately downstream and upstream of the putative FmPKS8 BGC, respectively, was performed. The resulting Δ*FMAN*_*15219* and Δ*FMAN*_*15224* strains were subsequently grown simultaneously as FmWT under *FmPKS8*‐inducing conditions, and culture filtrates were analyzed by HPLC‐HRMS. Deletion of neither Δ*FMAN*_*15219* nor Δ*FMAN*_*15224* impacted production of the target compounds **1**–**9** (Figure S7).

Thus, the FmPKS8 BGC comprises four genes i. e., *FMAN*_*15220*‐*FMAN*_*15223* encoding a putative Zn(2)‐C6 fungal‐type TF, a peptidase‐containing protein with an α/β hydrolase fold, an alcohol dehydrogenase domain‐containing protein predicted to own *trans*‐ER function and PKS8, respectively. Next to *F. mangiferae*, a functional PKS8 BGC is also present in *F. verticillioides* as the same FmPKS8‐related compounds, albeit to a lesser extent, were also produced in *F. verticillioides*. This is likely also true for *F. oxysporum* and *F. tjaetabe* based on collinearity of the DNA sequence. It is noteworthy to mention, that PKS8 orthologs in *F. fujikuroi* IMI58289, MRC2276, C1995, B14, E282 and FUS48 are moderately to strongly expressed in synthetic ICI with 6 mM glutamine as sole nitrogen source,^[27]^ while these conditions repress *PKS8* expression in *F. mangiferae* and *F. verticillioides*. This suggests distinct regulation mechanisms of the PKS8 BGC among the different fusaria, and may very well be attributed to the different lifestyles of the fungi.

### The fungal‐specific TF *FMAN*_*15220* positively regulates FmPKS8 cluster genes

**Figure 5:**
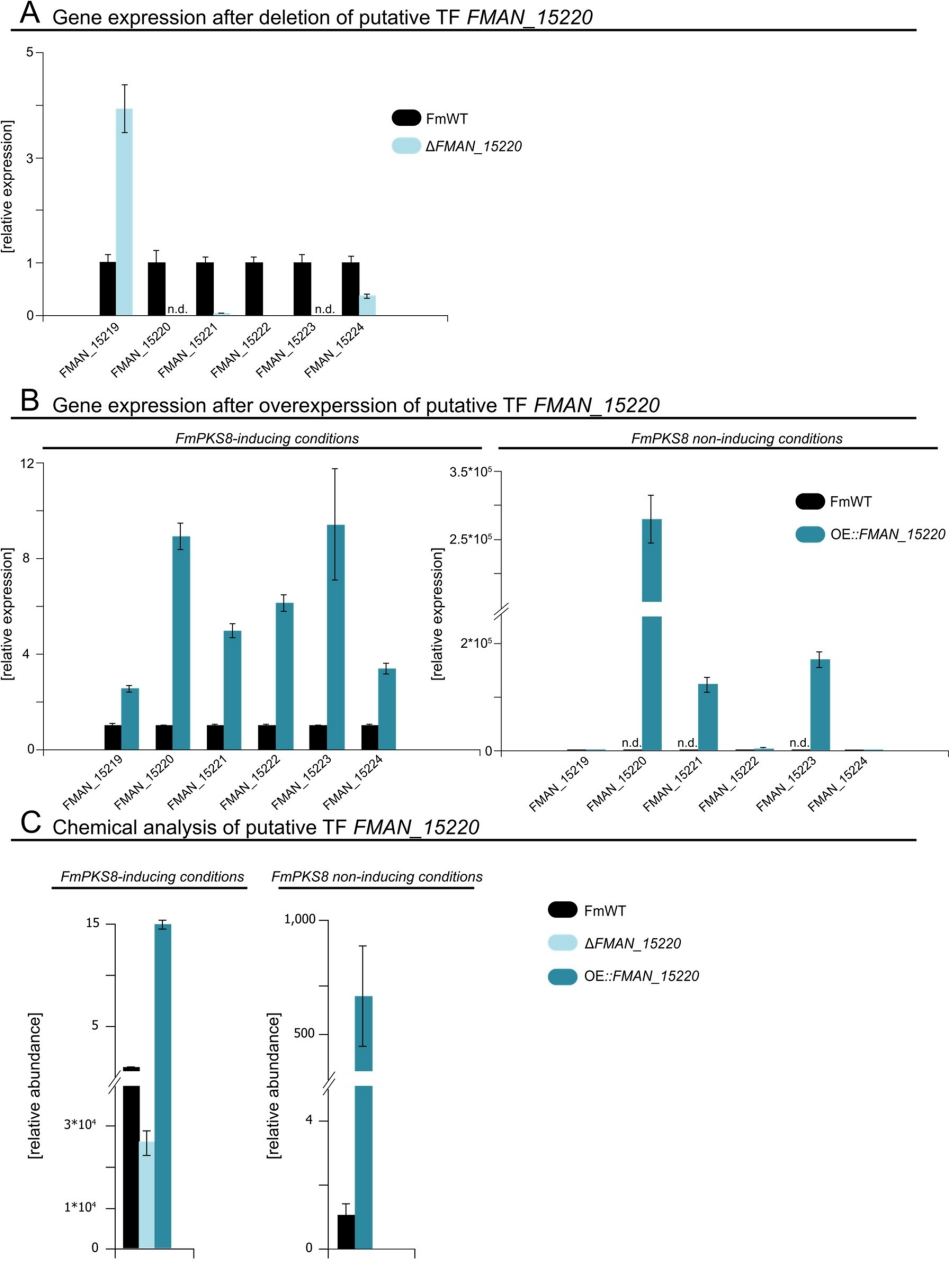
The Zn(2)‐Cys6 TF, *FMAN*_*15220*, positively regulated FmPKS8 cluster genes and verifies cluster borders. (A) Impact of *FMAN*_*15220* deletion on putative FmPKS8 cluster gene expression. *F. mangiferae* (FmWT) and Δ*FMAN*_*15220*, were grown in FmPKS8‐inducing conditions, and harvested three days post inoculation (dpi). Putative FmPKS8 cluster genes were significantly down regulated upon loss of *FMAN*_*15220*. (B) Constitutive strong overexpression of *FMAN*_*15220* induces expression of the remaining PKS8 cluster genes in inducing and non‐inducing culture conditions. FmWT and OE*::FMAN*_*15220*, were cultivated in *FmPKS8*‐inducing (6 mM NaNO_3_) and ‐repressing (60 mM glutamine) conditions, and mycelium was harvested 3 dpi. In *FmPKS8*‐inducing conditions, a significant upregulation of putative cluster genes was observed, including *FMAN*_*15218* and *FMAN*_*15219*, while in non‐inducing conditions gene expression of *FMAN*_*15220*‐*FMAN*_*15223* was de‐repressed by the overexpression strain. Gene expression for the cluster genes was determined by RT‐qPCR. The FmWT gene expression was arbitrarily set to 1. Mean values and standard deviations are shown. n.d., not detected. (C) Confirmation of expressional data *via* targeted metabolomics approach using HPLC‐HRMS measurements of supernatant samples of TF deletion (Δ*FMAN*_*15220*) and overexpression (OE*::FMAN*_*15220*) grown in different culture conditions. FmWT, Δ*FMAN*_*15220* and OE*::FMAN*_*15220* were cultivated in *FmPKS8*‐inducing (6 mM NaNO_3_) and ‐repressing (60 mM glutamine) conditions. Culture filtrates were collected 7 dpi, and measured *via* HPLC‐HRMS. Deletion of the putative TF‐encoding gene, *FMAN*_*15220*, led to a nearly abolished production of **1**–**9** under inducing conditions, while the overexpression of *FMAN*_*15220* produced significantly more of **1**–**9** compared to FmWT. Under *FmPKS8*‐repressing conditions, **1**–**9** were barely detectable in FmWT but abundant in OE*::FMAN*_15220. Experiments were performed in triplicates each. Mean values and standard deviations are shown in the diagram.

To explore the function of the putative Zn(2)‐C6 fungal‐type TF‐encoding gene within the FmPKS8 BGC, deletion as well as overexpression of *FMAN*_*15220* were approached resulting in Δ*FMAN*_*15220* and OE*::FMAN*_*15220*, respectively. Both strains and FmWT were cultivated for three days in either *FmPKS8*‐inducing or ‐repressing conditions (60 mM glutamine) for subsequent gene expression analysis. RT‐qPCR verified the involvement of *FMAN*_*15220* in the positive regulation of FmPKS8 cluster genes: deletion of *FMAN*_*15220* nearly abolished transcription of *FMAN*_*15221*‐*FMAN*_*15223* (Figure 5A), whereas overexpression of *FMAN*_*15220* led to a significant upregulation of all FmPKS8 cluster genes under *FmPKS8*‐inducing (6 mM NaNO_3_) as well as ‐repressing conditions (60 mM glutamine) (Figure 5B). Noteworthy, in *FmPKS8*‐inducing conditions, transcript levels of *FMAN*_*15219*, encoding a putative aconitase were increased in both Δ*FMAN*_*15220* and OE*::FMAN*_*15220*. For the latter, higher transcript levels were also observed in the case of the bordering genes, *FMAN*_*15218* and *FMAN*_*15224*. Though this observation is puzzling, cultivation under *FmPKS8*‐repressing conditions gave much clearer results. Here, cultivation of OE*::FMAN*_*15220* clearly showed that *FMAN*_*15220* positively affects *FMAN*_*15221*‐*FMAN*_*15223* gene expression, as transcripts of all three cluster genes were highly abundant under these normally repressing conditions, while no transcripts were detected for the bordering genes *FMAN*_*15218*, *FMAN*_*15219* and *FMAN*_*15224* (Figure 5B). It is noteworthy to mention, that under *FmPKS8*‐repressing conditions 400 times more *FMAN*_*15222* transcript is detected compared to FmWT, while *FMAN*_*15221* and *FMAN*_*15223* even revealed transcript levels exceeding 10,000 time the amount of FmWT. To further confirm the expression data, culture filtrates of Δ*FMAN*_*15220* and OE*::FMAN*_*15220* grown in either *FmPKS8*‐inducing and/or ‐repressing conditions were analyzed *via* HPLC‐HRMS, and biosynthesis of the target compounds **1**–**9** was compared to FmWT production levels. In agreement with the transcriptional data, **1**–**9** were abundant and increased in culture filtrates of FmWT and OE*::FMAN*_*15220*, respectively, but nearly abolished in Δ*FMAN*_*15220* under *FmPKS8*‐inducing conditions, whereas under *FmPKS8*‐repressing conditions **1**–**9** were absent from FmWT and only identified in cultures of OE*::FMAN*_*15220* (Figure 5C).

Thus, *FMAN*_*15220* encodes a pathway‐specific TF that positively regulates the expression of the FmPKS8 cluster genes i. e., *FMAN*_*15221*‐*FMAN*_*15223*. Moreover, nitrogen repression is circumvented by constitutively overexpressing *FMAN*_*15220*. These results further support the presence of a four‐gene cluster involved in the biosynthesis of FmPKS8‐related compounds **1**–**9**.

### Isolation and structural elucidation of the main FmPKS8 metabolites

**Figure 6:**
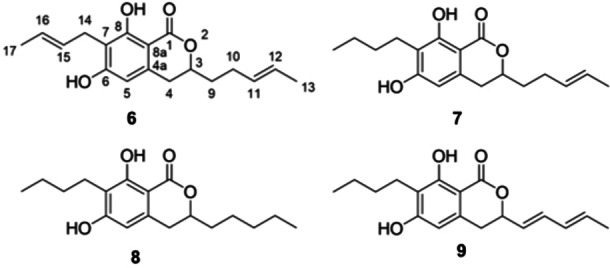
Structures of isolated dihydroisocoumarin derivatives **6**–**9**.

To elucidate the chemical structure of **1**–**9**, we took advantage of the high production levels in the OE*::FMAN*_*15220* strain. For this, OE*::FMAN*_*15220* was cultivated in *FmPKS8*‐inducing conditions for seven days, and fungal cultures were extracted with ethyl acetate (EtOAc), followed by separation and purification using preparative HPLC‐UV. Four of the nine target compounds were isolated in sufficient quantities to be structurally identified by NMR and HRMS. More precisely, compounds **6**–**9** (Figure 6) were isolated as pale‐white powders. Their chemical structures were characterized by HRMS, NMR spectroscopy, and in the case of **6** also by X‐ray diffraction. The substances were identified as dihydroisocoumarin derivatives carrying partially unsaturated pentyl and butyl residues at positions 3 and 7, respectively. The four representatives differ in the number and position of double bonds in the side chains, which are evident from the ^1^H chemical shifts and ^1^H, ^1^H COSY, HSQC and HMBC correlations (Figures S8–S31). Comparison of the spectral data of compounds **6**, **7** and **8** revealed their identity as 7‐But‐15‐enyl‐6,8‐dihydroxy‐3(*R*)‐pent‐11‐enylisochroman‐1‐one (**6**), 7‐Butyl‐6,8‐dihydroxy‐3(*R*)‐pent‐11‐enylisochroman‐1‐one (**7**) and 7‐Butyl‐6,8‐dihydroxy‐3(*R*)‐pentylisochroman‐1‐one (**8**).

**Figure 7:**
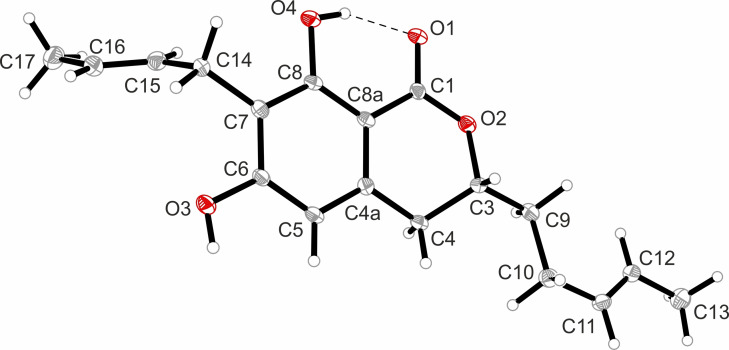
View of the one independent molecule of two found in the asymmetric unit of compound **6**. Thermal ellipsoids are shown at 30 % probability.

By recrystallization of **6** from MeCN/H_2_O single crystals suitable for X‐ray measurements were produced (Figure 7). The full set of the X‐ray data is found in the Supporting Information (Figure S32, Table S3).

Compound **9**, 7‐Butyl‐6,8‐dihydroxy‐3(*R*)‐pent‐9,11‐dienylisochroman‐1‐one, was found to be a regioisomer (positional isomer) to **6**, which does not contain a double bond at position 15 and instead carries a second double bond at the pentyl side chain at position 9.

To provide further proof that compounds **1**–**5** have similar structures and thus also originate from the FmPKS8 BGC, MS/MS spectra of compounds **1**–**5** (Figures S33–S37) were compared with those of compounds **6**–**9** (Figures S38–S41). To achieve a complex fragmentation pattern, stepped collision energy of CE 20, 45 and 70 eV was applied. Analysis of the MS/MS spectra of compounds **6**–**9** resulted in 121 fragment ions (all simply protonated ions; Table S4) and 30 typical neutral losses (Table S5), which appear to be characteristic for this group. For compounds **1**–**5** characteristic fragments and neutral losses as well as the percentage of agreement in relation to compounds **6**–**9** are listed in Table S6‐S10. Based on the degree of agreement between the fragments and neutral losses found in the MS/MS spectra, a shared biosynthetic origin of compounds **1**–**9** can be assumed.

Dihydroisocoumarins are ubiquitously found in the fungal kingdom and compounds **6**–**9** were described earlier for *Geotrichum* sp.^[29]^ On top of that, similar structures were isolated from cultures of *Acremonium strictum*,^[30]^ a marine‐derived *Aspergillus* sp.^[31]^ and *Cladosporium* sp.^[32]^ The 3,4‐dihydroisocoumarin derivates are also known as melleins and are produced by fungi, bacteria and plants alike^[33]^ For *Fusarium*, the first dihydroisocoumarin derivates were isolated from an unknown *Fusarium* sp. in 1970 and named fusamarins^[19]^ Given the same dihydroisocoumarin backbone and the similar structure of fusamarin identified in 1970, the target compounds **1**–**9** are from now on referred to as fusamarins (FMN). Next to this, similar compounds were also found in culture extracts from *F. verticillioides* M3125,^[34]^ that also harbors a functional PKS8 and the identified FMN BGC (Figure 4). In contrast to the current study, FMN were not detected in *F. verticillioides* wild‐type cultures, but only after the KS domain of Fum1 involved in fumonisin biosynthesis was exchanged for the KS domain of LovB responsible for lovastatin biosynthesis in *Aspergillus terreus*.^[34]^ Whether the absence of FMN in the *F. verticillioides* strain is due to the distinct cultivation conditions used in the different studies remains unclear at this point, but the origin of the dihydroisocoumarins in the mutated strains requires future attention.

It is noteworthy, that albeit presence of an intrinsic C‐Met domain, no C‐methylation was observed in the isolated metabolites (**6**–**9**). Similarly, also compounds **1**–**5** appear to be not C‐methylated (see Table 1). Overall C‐Met domains belong to a multifaceted superfamily, characterized by low sequence identity,^[35]^ which impedes the *in silico* analysis of C‐Met domains in general and for FmPKS8 in particular. It is, however, not uncommon for a HR‐PKS to retain an inactive C‐Met domain,^[36]^ suggesting that the C‐Met domain in the FMN BGC is indeed non‐functional in *F. mangiferae* and *F. verticillioides*.

### Cytotoxicity test

**Figure 8:**
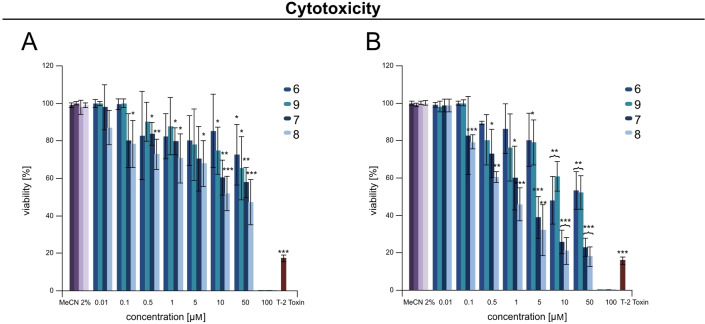
Viability of HepG2 (A) and HT29 (B) cells after treatment with FMN (**6**–**9**). The results represent the sum of three individual experiments each with three triplicates (n≥9); * p≤0.05, ** statistically significant (p≤0.01), *** statistically highly significant (p≤0.001); positive control: T‐2 toxin (10 μM).

Although several bioactive effects, including antimalarial, antituberculosis and antifungal activities were described previously for the compounds **6**, **7** and **8**,^[29]^ data about their cytotoxicity (**6**–**9**) is not available thus far. In general, dihydroisocoumarins were shown to possess moderate toxic effects.^[37]^ Moreover, previous studies confirmed the contribution of the lipophilic residues at both dihydroisocoumarin rings on the toxic effects.[^37^, ^38^] Here, the possible cytotoxicity of the isolated dihydroisocoumarins (**6**–**8**) from OE*::FMAN*_*15220* was tested against human liver cancer cells (HepG2) and human colon cancer cells (HT29) (Figure 8). The viability of the cells was determined by using resazurin assay after incubation of both cell lines with test compounds independently for 24 h. Interestingly, on both cell lines compounds **6** and **9** were demonstrating moderate cytotoxicity. A dramatic decrease in cell viability for those metabolites was observed after the tested concentration reached and exceeded 50 μM. In contrast compounds **7** and **8** were found to be toxic against both cell lines starting already at 0.5 μM, demonstrating higher effects when tested against HT29 cells (Figure 8B). Thus, IC_50_ values for the compounds **7** and **8** were calculated as 2.2 μM and 1 μM, respectively.

### Verification and functional characterization of putative PKS8 cluster genes

**Figure 9:**
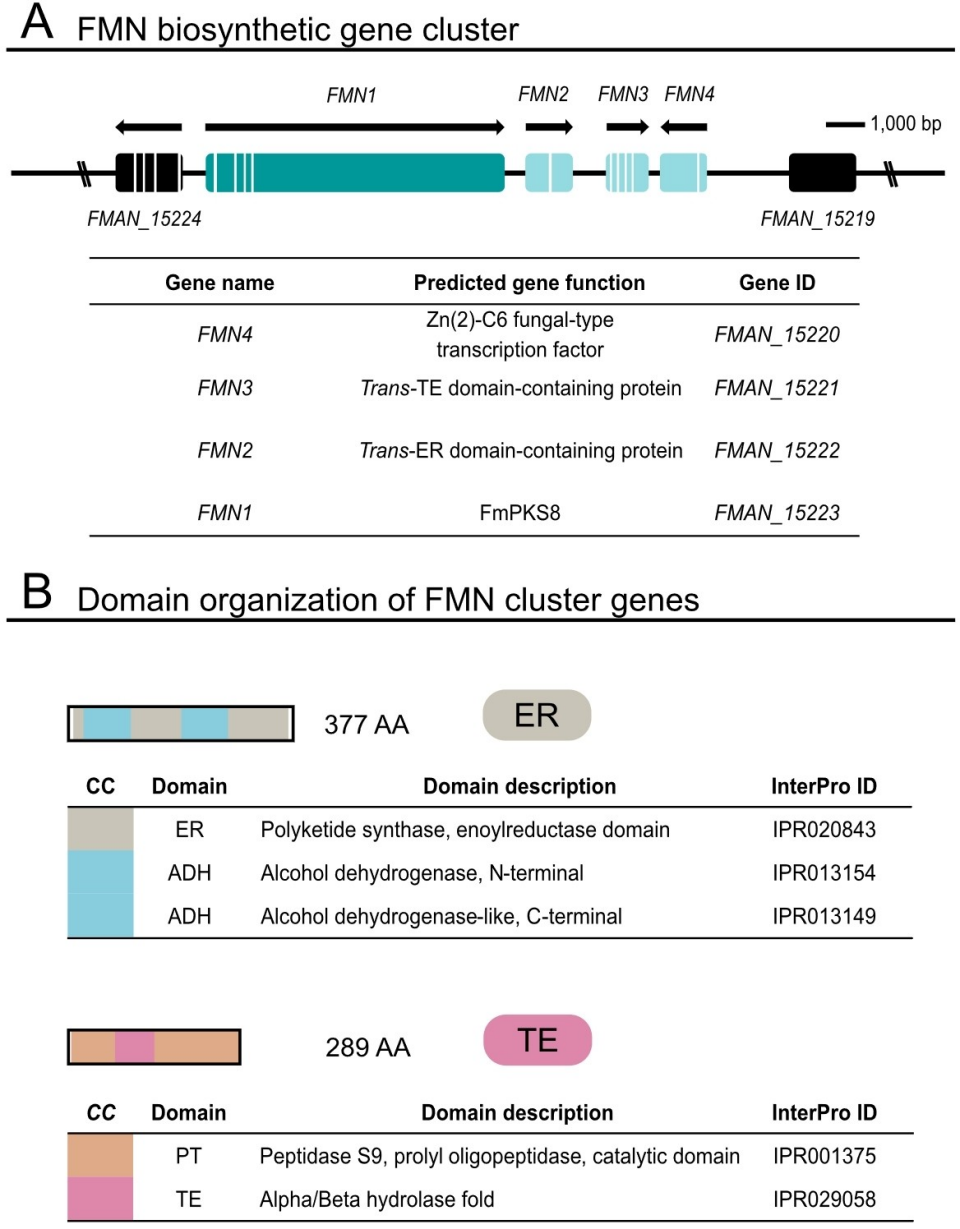
Functional characterization of additional FMN cluster genes. (A) The turquoise rectangle depicts the key enzyme *FmFMN1* (*FmPKS8*), and the light turquoise rectangles designate to *FmFMN2*, *FmFMN3* and *FmFMN4*, cluster genes necessary for the biosynthesis of **1**–**9**. Black rectangles indicate genes adjacent to the FMN BGC. The direction of transcription is indicated with arrows and white bars represent intronic sequences. (B) Detailed depiction of FmFmn2 and FmFmn3 domains. FmFmn2 harbors an ER domain as well as domains with putative alcohol dehydrogenase function (ADH), while FmFmn3 owns a peptidase domain including an α/β hydrolase fold. The domain structure was determined using InterPro^[1]^ database for depiction. CC denotes color code.

The FMN BGC comprises four genes (Figure 9A). These include, next to *FmFMN1* and *FmFMN4* encoding the PKS8 key enzyme (*FMAN*_*15223*) and a pathway‐specific TF (*FMAN*_*15220*), respectively, *FMAN*_*15221* and *FMAN*_*15222* (from now on referred to as *FmFMN2* and *FmFMN3*, respectively). FmFmn2 harbors a peptidase and an α/β hydrolase fold domain, and FmFmn3 owns an alcohol dehydrogenase as well as an ER domain (Figure 9B). To characterize the remaining two FmFMN cluster genes in more detail and to evaluate their contribution to the biosynthesis of **1**–**9**, *FmFMN2* (*FMAN*_*15221*) and *FmFMN3* (*FMAN*_*15222*) were deleted independently in the FmWT background to create single mutant strains. Next, FmWT was simultaneously grown with Δ*fmfmn1*, Δ*fmfmn2*, Δ*fmfmn3* and Δ*fmfmn4* strains for three days under *FmFMN*‐inducing conditions. Subsequent expression analysis by RT‐qPCR showed that loss of either of the FMN cluster genes, except for Δ*fmfmn4* encoding the pathway‐specific TF, did not affect expression of the remaining intact FMN cluster genes (Figure 6, Figure S42), a pre‐requisite to further analyze the function of FmFmn2 and FmFmn3. To assess their role in the biosynthesis of **1**–**9**, Δ*fmfmn2* and Δ*fmfmn3* were grown in parallel with FmWT for seven days in *FMN*‐inducing conditions, and the culture filtrates were subjected to HPLC‐HRMS measurements. While **1**–**9** were abundant in FmWT samples, no FMN or any related compounds were formed in any of the deletion strains, Δ*fmfmn2* and Δ*fmfmn3*. This suggests that both FmFmn2 and FmFmn3 are vital for the biosynthesis of **1**–**9**.

In general, *trans*‐acting ER domains are commonly found in BGCs of fungal HR‐PKS lacking a functional ER domain. This is exemplified by e. g., *lovC*, *mokE*, *mlcG*, *eqi9/fsdC*, *apdC*, *tenC*, *dmbC*, *cheB*, *ccsC*, *eqxC*, *fsl5* and *CalK* responsible for lovastatin, monacolin, compactin, fusaridione, aspyridone, tenelin, desmethylbassianin, chaetoglobosins, cytochalasin E and K, eqisetin, fusarilien and calbistrins[^39^, ^40^, ^41^] biosynthesis, respectively. These proteins are typically encoded within the same fungal BGC and act cooperatively with the PKS to assemble the final product. Presence of the exogenous ER domain appears as a prerequisite for the correct product assembly as for example shown for lovastatin, aspyridione and fusarilien biosynthesis.[^39^, ^41^, ^42^] Here, lack of the *trans*‐ER domain led to an abolished production of the final polyketide, a finding that is in agreement with the absence of **1**–**9** upon the loss of FmFmn2. Thus, it is tempting to speculate that FmFmn2 works together with FmFmn1 to implement the final reduction steps of the polyketide backbone yielding the identified fusamarins (**1**–**9**).

In general, TE domains belong to the superfamily of α/β hydrolase fold‐containing proteins, such as FmFmn3, and can be subdivided into two distinct classes. TE modules that are located at the C‐terminus of PKSs/NRPSs and facilitate the offloading of the nascent polyketide are categorized as Class I. In contrast, TE domains of Class II are either located at the C‐terminal end of the PKSs/NRPSs or even found as stand‐alone proteins typically encoded within the same BGC. Next to this, Class II TEs comprise two distinct functions: they may either be responsible for the removal of aberrant intermediates from the acyl carrier domain or involved in the product release *via* hydrolyzation or macrocyclization (lactam or lactone formation) from the ACP domain of the emerging product.[^9^, ^43^] An additional function of the exogenous TE was proposed for the biosynthetic pathway of brefeldin.^[44]^ Here the *trans*‐TE domain Bref‐TE is associates with the respective PKS (Bref‐PKS) and by that determines the chain length of the assembled product. Loss of the Bref‐TE led to a significant decrease in brefeldin biosynthesis. Similar observations were made in fusarilien and lovastatin biosynthesis. Here, loss of the exogenous TEs Fsl2 and LovG, respectively, resulted in abolished biosynthesis of both products,[^39^, ^45^] which is in agreement with the abolished biosynthesis of **1**–**9** in Δ*fmfmn3* compared to FmWT. Thus, FmFmn1, FmFmn2 and FmFmn3 are all essential for FMN biosynthesis (**1**–**9**).

**Figure 10:**
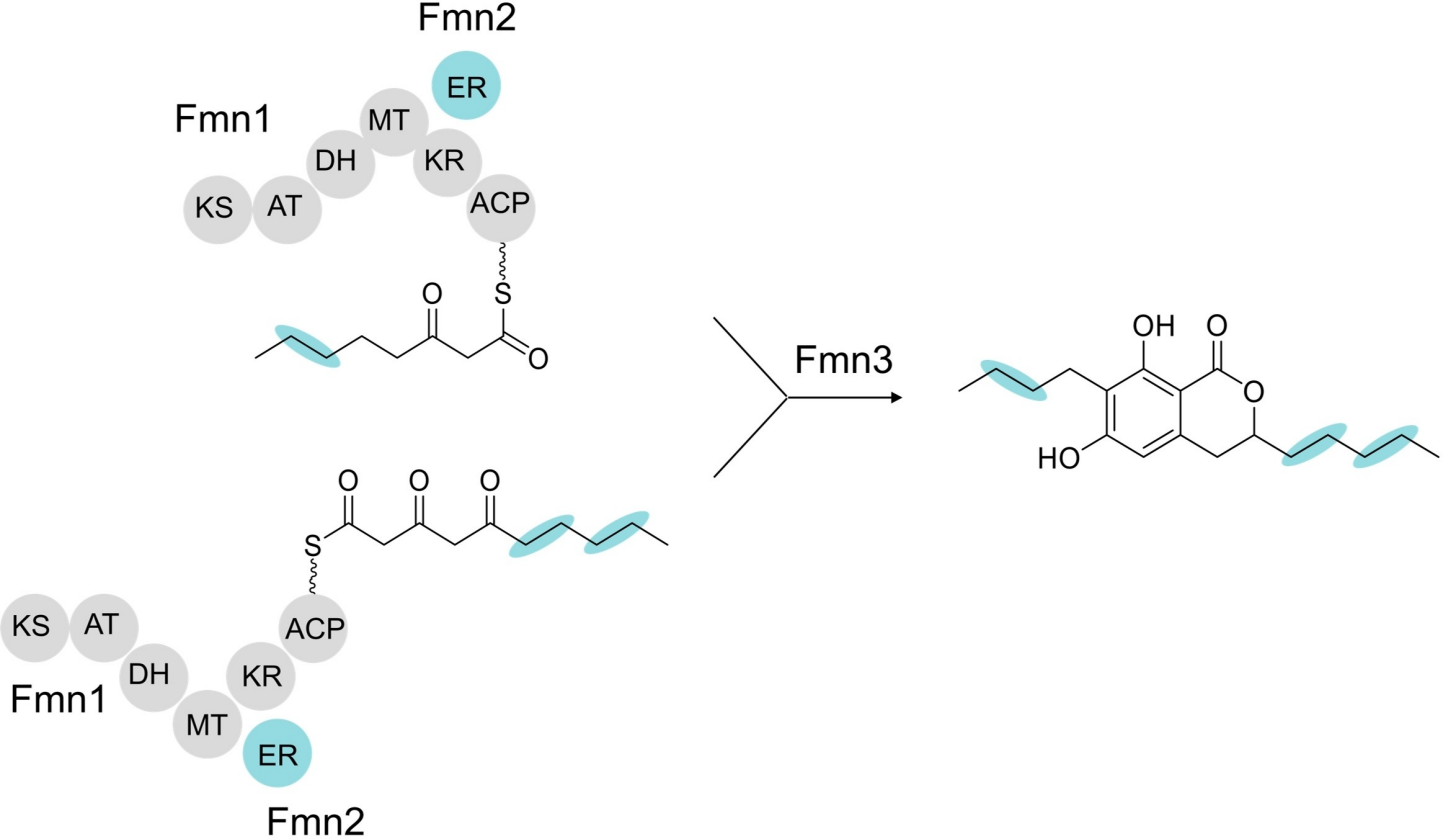
Proposed biosynthetic pathway of FMN.

Interestingly, the structural properties of the isolated FMN suggest that their biosynthesis involves the fusion of two distinct carbon chains. Since only one PKS is encoded within the FMN BGC and no additional PKS‐encoding gene was found in close proximity, we propose that FmFmn1 synthesizes two different polyketides, a tetra‐ and a pentaketide, containing a varying number of double bonds depending on the selective actions of the *trans*‐acting ER FmFmn2. Chain fusion will presumably be mediated by the KS domain before finally offloading is catalyzed by the α/β hydrolase fold enzyme FmFmn3 (Figure 10). A similar mechanism has only recently been elegantly demonstrated for gregatin A biosynthesis in *Penicillium* sp. sh18,^[46]^ and illustrates the versatile biochemical reactions catalyzed by fungal type I PKSs. Next to FmFmn1‐3, FmFmn4 functions as a positive TF and governs *FmFMN* gene expression.

## Conclusion

Through a combination of molecular, chemical and bioinformatic analyses we show that the previously cryptic PKS8 BGC is involved in biosynthesis of the dihydroisocoumarins **1**–**9** (also referred to as fusamarins, FMN) in *F. mangiferae*. FMN biosynthesis is negatively influenced by the loss (Δ*fmkmt1*) or prevention (H3K9R) of H3K9me3 and repressed by nitrogen as well as an acidic pH in *F. mangiferae*. The FMN BGC comprises four genes encoding the HR‐PKS FmFmn1 (*FmFMN1*), a *trans*‐ER domain containing protein (*FmFMN2*), a *trans*‐TE containing protein (*FmFMN3*) as well as a fungal‐type TF (*FmFMN4*). FmFmn4 functions in the transcriptional activation of FmFmn1‐FmFmn3, and overexpression of *FmFMN4* de‐represses *FMN* genes in high nitrogen. FmFmn1 and FmFmn2 likely function in the synthesis of two different carbon chains, that are fused and hydrolyzed by FmFmn3 giving rise to the dihydroisocoumarins FMN (Figure 10).

Next to *F. mangiferae*, an orthologous FMN BGC is also present in other members of the FFSC, including *F. verticillioides* and *F. tjaetabe* as well as members of the *Fusarium nisikadoi* species complex (FNSC) and distinct *Fusarium* isolates including *Fusarium austroafricanicum* and *Fusarium babinda*. Expression of *FmFMN* is induced under identical conditions in *F. mangiferae* and *F. verticillioides*, possibly pointing towards a conserved role of these SMs in both species. FMN are described to own several bioactive properties. Here, we show the cytotoxic properties of **6**–**9**. All isolated dihydroisocoumarin derivatives demonstrate moderate (cyto)toxicity when tested on HepG2 cells. The HT29 cells, however, were found to be more sensitive to metabolites **7** and **8** with IC_50_ values of 2.2 μM and 1 μM, respectively.

## Experimental Section

**Fungal strains and culture conditions**: *F. mangiferae* wild‐type isolate MRC7560 (FmWT) originated in Israel (National Research Institute for Nutritional Diseases, Tygerberg, South Africa) and kindly provided by Stanley Freeman, was used to generate deletion (Δ*FMAN*_*15219*, Δ*FMAN*_*15220*, Δ*FMAN*_*15221*, Δ*FMAN*_*15222*, Δ*FMAN*_*15224*) and overexpression (OE*::FMAN*_*15220*) strains. Next to this, the Δ*fmkmt1* deletion strain, the partial gene deletion Δ*fmPKS8* and the amino acid exchange of histone 3 (H3) lysine9 (K9) to arginine 9 (R9) (FmH3K9R) were used in this study but generated elsewhere.^[17]^ The *F. verticillioides* wild type M3125, was kindly provided by D. Brown from the U.S. Department of Agriculture, U.S.A and used for expressional analysis and the partial gene deletion of Δ*fvPKS8*.

For the extraction of genomic DNA (gDNA), fungal mycelia were collected after 2–3 days growth on solid complete medium (CM).^[47]^ All plates were covered with cellophane sheets (FoliaBringmann) and incubated at 30 °C in the dark. To assess fungal growth, strains were cultivated on solid CM, V8 (30 mM CaCO3, 20 %, v/v, vegetable juice; Campbell Food, Puurs, Belgium), and synthetic ICI medium^[48]^ supplemented with 6 mM glutamine (Carl Roth). Plates were inoculated with 10μL of a 10^5^ conidia/mL solution and incubated at 30 °C in the dark for 5 days. Production of conidiospores was triggered on solid V8 medium, inoculated with a 5 mm agar plug each and incubated at 20 °C for 7 days under the presence of 16 h light and 8 h dark (L/D) or dark conditions (D) and 70 % humidity. Conidia were quantified with a Neubauer Improved hemocytometer under a light microscope (Carl Zeiss). For fungal liquid cultivation pre‐culturing in 100 mL Darken medium^[49]^ inoculated with two 5 mm plugs in a 300 mL Erlenmeyer flask for 3 days in the dark at 30 °C and 180 rpm was performed. Next, 0.5 mL pre‐culture was transferred to liquid ICI media supplemented with either 6 or 60 mM glutamine or with 6 or 120 mM NaNO_3_ (Carl Roth) as sole nitrogen source and incubated on an orbital shaker (180 rpm, 30 °C). Mycelia were harvested after 3–7 or 7 days for RNA or SM analysis, respectively.

**Plasmid construction**: For cloning of gene deletion as well as overexpression vectors yeast recombinational cloning was performed as described earlier. All primers used for the amplification of DNA sequences were obtained from Merck and are listed in Table S2. For the amplification of PCR products, the Q5 high‐fidelity DNA polymerase (New England Biolabs) was used. Gene deletion constructs were generated by the amplification of approximately 1kb upstream (5’) and downstream (3’) of the gene of interest using FmWT gDNA as template by the respective primer pairs 5F/5R and 3F/3R. The hygromycin B resistance (hygR) cassette was used as resistance marker throughout this study for all deletion constructs. Therefore, the cassette was amplified using the primer pair hphF and hphR from the template vector pCSN44.^[50]^ For the in loco overexpression of the pathway specific TF *FMAN*_*15220* again of approximately 1kb of the 5’ region of *FMAN*_*15220* was amplified using FmWT gDNA as template. Next to this, the primer YRC_pOE‐FMAN_15220_F was paired with the respective 3R primer to amplify the native WT gene including the 3’ region, including its native terminator sequence from FmWT gDNA. Next to this, the strong constitutive olic promoter from *Aspergillus nidulans* coupled to the hygR cassette was amplified from a *Sac*II/*Eco*RI digested shuttle vector pNAH‐OGG.^[51]^ The *Eco*RI/*Xho*I‐digested shuttle vector pRS426,^[52]^ served as backbone construct for both deletion and overexpression constructs. The yeast *Saccharomyces cerevisiae* FY834 strain was used for the assembly of the final constructs.^[53]^

**Standard molecular techniques**: Cultivations of *S. cerevisiae* and *Escherichia coli* were performed as described elsewhere.^[51]^ Yeast plasmid DNA (pDNA) was extracted using the GeneJET Plasmid MiniprepKit (Thermo Fisher Scientific) and directly used as template for the amplification of the linear gene replacement cassettes from the circular vector. Transformation of the OE*::FMAN*_*15220* plasmid was performed using the *E. coli* DH5α (InvitrogenTM) strain as described in the manufacturer's procedure. Subsequent extraction of pDNA was performed using the GeneJET Plasmid Midiprep Kit (Thermo Fisher Scientific) as described in the manufactures protocol. Correctness of the overexpression plasmid was verified *via* sequencing (LGC Genomics, Germany). Sequencing primers are listed in Table S2.

For expressional analysis, lyophilized and ground mycelium from SM‐producing cultivation under standard conditions (ICI supplemented with 6 or 120 mM NaNO_3_, 6 or 60 mM glutamine) for 3–7 days was used for RNA isolation. RNA was isolated using the reagent TRIzol (Thermo Fisher Scientific) according to the manufacturer's instructions.

**Fungal transformation and strain generation**: Generation of *F. mangiferae* and *F. verticillioides* protoplast followed by fungal transformation was performed as described elsewhere.^[54]^ The linear gene deletion constructs were amplified from the vectors pΔ*FMAN*_*15219*, pΔ*FMAN*_*15220*, p*ΔFMAN*_*15221*, pΔ*FMAN*_*15222* and pΔ*FMAN*_*15224* using the respective primer pair 5F/3R using Q5‐high fidelity polymerase, while the 10 μg of the pOE*::FMAN*_*15220* vector was digested with *Eco*RV prior transformation. Since homologous recombination events for *F. verticillioides* are low the split marker approach^[55]^ was performed for to partial gene deletion of *FvPKS8*. Here, fragments were amplified from pΔ*fvPKS8* with the primer pairs 5F/Split‐mark_hphF and 3R/Split‐mark_hphR (Supporting Information Table S2).

Selection of fungal transformants was ensured using minimal regeneration media supplemented with 100 ppm hygromycin B (Merck Millipore).

Extraction of genomic DNA from lyophilized and ground mycelia from transformants was performed as described earlier.^[56]^ Successful homologous integration events of both, deletion and overexpression constructs and absence or presence of the respective wild‐type gene, was verified by diagnostic PCR using GoTaq® DNA Polymerase (Promega). For deletion the respective primer pairs dia5F/pCSN44_trpCT and dia3F/pCSN44_trpcP2 were used to verify in loco integration, while absence (deletion) and presence (overexpression) of the wild‐type gene was tested using the primer pair diaWT_F/R. In loco integration of the overexpression construct was verified using the primer pairs dia5F/pCSN44‐hph‐trpC−T and dia3R2/WTdia5F.

Absence of the respective wild‐type genes as well as the constitutive overexpression of *FMAN*_*15220* was additionally verified via RT‐qPCR.

The deletion of *FMAN*_*15219* and *FMAN*_*15220* yielded in one independent transformant each i. e., Δ*FMAN*_*15219*_T16 (Supporting Information Figure S43) and Δ*FMAN*_*15220*_T8 (Supporting Information Figure S44). Deletion of *FMAN*_*15221* resulted in three independent deletion mutants Δ*FMAN*_*15221*_T1, T5 and T8 (Supporting Information Figure S45), while deletion of *FMAN*_*15222* led to the transformation of two independent mutant Δ*FMAN*_*15222*_T1 and T7 (Supporting Information Figure S46). Deletion of *FMAN*_*15224* resulted in three independent deletion mutants i. e., Δ*FMAN*_*15224*_T2, T10 and T11 (Supporting Information Figure S47). The partial gene deletion of *FvPKS8* resulted in five independent transformants designated as Δ*fvPKS8*_T3, T4, T7, T13, T31 (Supporting Information Figure S48). For the *FMAN*_*15220* overexpression strain four independent transformants i. e., OE*::FMAN*_*15220*_T16, T24, T32, T39 (Supporting Information Figure S49A) were obtained, which showed in locus integration of the construct (Supporting Information Figure S49B) as well as overexpression of *FMAN*_*15220* (Supporting Information Figure S49C).

**Expressional Analysis by semi‐quantitative PCR and quantitative RT‐qPCR**: For expressional analysis 1 μg RNA was transcribed to cDNA using the LunaScript^TM^ RT SuperMix Kit (New England Biolabs). Semi‐quantitative PCR reactions were performed using GoTaq®G2 DNA Polymerase and cDNA served as a template. The fragments were amplified using RT‐qPCR primers and 30 cycles per run. To compare cDNA quantities, the house‐keeping gene actin (*FMAN*_*05925*) was amplified with the primer pair Actin_F/R. For RT‐qPCR analysis the SYBR©Green Supermix (Bio‐Rad) was used for quantitation in an iCycler iQ Real‐Time PCR System (Bio‐Rad). Primer efficiency was determined and set between 90 and 110 %, while Ct values greater than 31 were taken as not expressed. Results were calculated according to the ΔΔCt^[57]^ method. Gene expression of genes of interest was normalized to the expression of actin (*FMAN*_*05925*), Glyceraldehyde‐3‐phosphate dehydrogenase (GPD, *FMAN*_*05925*), and β‐tubulin (*FMAN*_*07563*). Used primers are listed in Supporting Information Table S2.

**Secondary metabolite production and sample preparation**: For chemical analysis, fungal culture was retrieved from liquid ICI (supplemented with different nitrogen sources and concentrations) was incubated at 30 °C for seven days in the dark. The fungal cultures were quenched 30 % v/v with ice cold acetonitrile (MeCN) (obtained from Honywell, Seelze, Germany) and subsequently centrifuged. The pre‐diluted supernatant was then applied to HPLC‐HRMS analysis.

**HPLC‐HRMS for the analysis of fungal culture supernatant samples**: Prepared culture supernatant samples were diluted 10‐fold with ddH_2_O/MeCN 7 : 3 (*v*/*v*) before measurement and analyzed using high performance liquid chromatography‐high resolution mass spectrometry (HPLC‐HRMS). Chromatographic separation was performed on a Thermo Vanquish Duo UHPLC system interfaced with an Orbitrap QExactive HF mass spectrometer (Thermo Fisher Scientific, San Jose, CA, USA), equipped with a heated electrospray ionization (HESI‐II) source operated in fast polarity switching mode (1 s/cycle); scan range 100–1,500 *m*/*z*; resolving power 120,000 @ 200 *m*/*z*. The LC conditions were as follows: column, XBridge BEH C18 column (150×2.1 mm i.d., 3.5 μm particle size, Waters, Milford, MA, USA); mobile phase, (**A**) ELGA Purelab Ultra water (VWS, High Wycombe, UK) with 0.1 % formic acid (obtained from Carl Roth, Karlsruhe, Germany) and (**B**) methanol (obtained from Honywell, Seelze, Germany) with 0.1 % formic acid; flow rate 250 μL/min; temperature 25 °C; injection volume, 2 μL; separation started with an initial isocratic step at 10 % of **B** for 2 min followed by a linear gradient of 10 % to 100 % of **B** over 30 min, an isocratic step at 100 % of **B** for 5 min, a gradient of 100 to 10 % of **B** over 0.1 min and finally an isocratic step at 100 % of **B** for 7.9 min. The HESI‐II parameters were as follows: source voltage, 3.5 kV (pos), −3.3 kV (neg); sheath gas flow rate (N_2_), 50 units; auxiliary gas flow rate, 11 units; spare gas flow rate, 2; capillary temperature, 320 °C; and S‐Lens RF Level, 55.

**Untargeted metabolomics workflow**: For the untargeted comparative metabolite profile data evaluation Compound Discoverer Software, version 3.1.0.305 (Thermo Fisher Scientific, San Jose, CA, USA) was used. An untargeted metabolomics workflow was applied including feature detection in the individual samples, grouping of identified features across all samples, filling of gaps across all samples, hiding of chemical background by means of removal of all features also found in culture media blank samples (r=3) or extraction solvent blank samples (r=3), predicting the elemental composition for found compounds and finally identifying the differences between the defined sample groups, FmWT (r=3) vs. Δ*fmPKS8* (r=3) and FmWT (r=3) vs. Δ*fmPPT1* (r=3).

The data processing workflow included the following nodes and settings: “Select Spectra” (with lower RT limit 2 min and upper RT limit 37 min), “Align Retention Times” (Adaptive curve mode, max. Shift 0.5 min, max mass tolerance 5 ppm), “Detect Compounds” (mass tolerance 5 ppm, intensity tolerance 30, min peak intensity 1E6 counts), “Group Compounds” (mass tolerance 5 ppm, RT tolerance 0.2 min), “Fill Gaps” (mass tolerance 5 ppm, S/N threshold 1.5), “Mark Background Compounds” for removal of signals derived from cultivation media (r=3) or extraction solvent (r=3) (max. sample/blank ratio 5, max blank/sample ratio 0, hide background true) and finally “predict composition” (mass tolerance 5 ppm; min. element counts C_2_, H_4_; max element counts C_90_, H_190_, N_10_, O_18_, P_3_, S_5_; min. C/H 0.1, max. C/H 4.0) and as post‐processing node “Differential Analysis” of sample groups (*t*‐test and ANOVA, determination of *p*‐values, ratios and fold‐change as well as CV).

**Targeted metabolomics workflow**: For the targeted screening of the tentative PKS8 metabolite candidates in culture supernatant samples of different fungal KO or overexpressing strains, the XCMS R‐package^[58]^ was used. The target features ([M+H]^+^ ions in positive mode or [M−H]^−^ ions in negative mode) were searched for and peak areas were integrated using the findChromPeaks() function of XCMS utilizing the Centwave peak picking algorithm. A maximum *m*/*z* deviation of +/−3 ppm and a maximum retention time deviation of +/−4 seconds was permitted.

**NMR and X‐ray diffraction analysis**: NMR spectra (^1^H, ^13^C, gHMBC, gHSQC, and gCOSY) were recorded with an Agilent DD2 600 MHz spectrometer (Agilent Technologies, Waldbronn, Germany); chemical shifts (*δ*) are reported in ppm relative to tetramethylsilane. Optical rotation, α [deg], was determined with a UniPol L1000 polarimeter (Schmidt+Haensch, Berlin, Germany), path length 1 dm, wavelength 589 nm (sodium D line); the unit of specific rotation ${\left[\alpha \right]}_{D}^{20}$ [deg ⋅ mL ⋅ dm^−1^ ⋅ g^−1^] is omitted; the concentrations of the sample c [mg ⋅ mL‐1] and solvent used are given in brackets. The UV spectra and purity measurements were recorded on an LC‐UV‐ELSD with LC PU‐2089 system with UV detection (MD‐2010) (Jasco, Groß‐Umstadt, Germany) and evaporative light scattering detection (ELSD) (Shimadzu, Duisburg, Germany). High‐resolution mass spectrometric data for structural characterization were obtained using an LTQ Orbitrap XL^TM^ mass spectrometer (Thermo Fisher Scientific, Dreieich, Germany) operated in positive mode with a resolution of 60000 and direct sample injection of a 10 μg/mL solution in MeCN/H_2_O/FA (50/49.9/0.1, *v*/*v*/*v*). The heater was turned off, the capillary temperature was set to 275 °C, and the sheath gas was set to 5 arbitrary units. The auxiliary and sweep gases were switched off. The spray voltage, capillary voltage, and tube‐lens voltage were put to 4 kV, 30 V, and 80 V, respectively.

X‐ray diffraction. Data sets for compound **6** were collected with a Bruker D8 Venture Photon III Diffractometer. Programs used: data collection: APEX3V2019.1‐0;^[59]^ cell refinement: SAINT V8.40A;^[59]^ data reduction: SAINT V8.40A;^[59]^ absorption correction, SADABS V2016/2;^[59]^ structure solution SHELXT‐2015:^[60]^ structure refinement SHELXL‐2015^[61]^ and graphics, XP.^[62]^ R‐values are given for observed reflections, and wR2 values are given for all reflections. Exceptions and special features: For compound **6** one −CH_2_−CH=CH−CH_3_ group was found disordered over two positions in the asymmetric unit. Several restraints (SADI, SAME, ISOR and SIMU) were used in order to improve refinement stability.

**X‐ray crystal structure analysis of 6**: A colorless, needle‐like specimen of C_18_H_22_O_4_, approximate dimensions 0.047 mm×0.049 mm×0.211 mm, was used for the X‐ray crystallographic analysis. The X‐ray intensity data were measured on a single crystal diffractometer Bruker D8 Venture Photon III system equipped with a micro focus tube Cu ImS (CuKα, λ=1.54178 Å) and a MX mirror monochromator. A total of 2030 frames were collected. The total exposure time was 27.34 h. The frames were integrated with the Bruker SAINT software package using a wide‐frame algorithm. The integration of the data using a triclinic unit cell yielded a total of 12828 reflections to a maximum θ angle of 66.66° (0.84 Å resolution), of which 5275 were independent (average redundancy 2.432, completeness=99.8 %, R_int_=7.15 %, R_sig_=7.06 %) and 4219 (79.98 %) were greater than 2σ(F^2^). The final cell constants of a=5.2255(1) Å, b=10.5198(2) Å, c=15.1266(4) Å, α=75.893(2)°, β=87.542(2)°, γ=82.119(2)°, volume=798.79(3) Å^3^, are based upon the refinement of the XYZ‐centroids of 4855 reflections above 20σ(I) with 6.024°<2θ<133.2°. Data were corrected for absorption effects using the Multi‐Scan method (SADABS). The ratio of minimum to maximum apparent transmission was 0.852. The calculated minimum and maximum transmission coefficients (based on crystal size) are 0.8640 and 0.9670. The structure was solved and refined using the Bruker SHELXTL Software Package, using the space group *P*1, with Z=2 for the formula unit, C_18_H_22_O_4_. The final anisotropic full‐matrix least‐squares refinement on F^2^ with 446 variables converged at R1=5.43 %, for the observed data and wR2=14.00 % for all data. The goodness‐of‐fit was 1.028. The largest peak in the final difference electron density synthesis was 0.192 e^−^/Å^3^ and the largest hole was −0.236 e^−^/Å^3^ with an RMS deviation of 0.052 e^−^/Å^3^. On the basis of the final model, the calculated density was 1.257 g/cm^3^ and F(000), 324 e^−^. Two independent molecules of compound **6** were found in the asymmetric unit. Flack parameter was refined to 0.1(2). The hydrogen atoms at O3, O4, O7 and O8 were refined freely (Figure 7). Deposition Number 2178933 contains the supplementary crystallographic data for this paper. These data are provided free of charge by the joint Cambridge Crystallographic Data Centre and Fachinformationszentrum Karlsruhe Access Structures service www.ccdc.cam.ac.uk/structures.

**Isolation of dihydroisocoumarin derivatives**: The OE*::FMAN*_*15220* strain was pre‐cultured in 125 mL Darken medium^[49]^ in a 300 mL Erlenmeyer flask in the dark (30 °C, 180 rpm). After 3 days, 125 mL ICI‐glucose medium supplemented with 6 mM NaNO_3_ were inoculated with 0.5 mL of the pre‐culture and incubated at 180 rpm, 30 °C in the dark for 7 days. The cultures (8×125 mL) were extracted (3×) with EtOAc and the crude extract was partitioned between H_2_O and EtOAc. The EtOAc‐soluble fraction was evaporated in vacuo and redissolved in 15 mL MeCN/H_2_O (50/50, *v*/*v*). The extract was centrifuged at 3000×*g* for 5 min and further filtrated through a 15 mm syringe filter containing a 0.2 μm pore size RC membrane (Phenomenex, Aschaffenburg, Germany). Purification of compounds **6**–**9** was performed by preparative LC‐UV on an LC‐UV Jasco HPLC PU‐2087/2087 system and a UV‐2075 detector (Jasco, Groß‐Umstadt, Germany). Separation occurred on a Symmetry C8 Prep Column (7 μm, 19×300 mm, Waters, Eschborn, Germany) applying a binary gradient consisting of MeCN (solvent **A**) and H_2_O (solvent **B**) both containing 0.1 % FA at a consistent flow rate of 15 mL/min and a detection wavelength at 230 nm. The gradient started with 30 % **A** for 2 min followed by linearly increasing the percentage of **A** to 75 % within 10 min and holding a plateau for 8 min. Within 5 min **A** was increased to 95 %. After holding **A** at 95 % it was decreased to 30 % again for 2 min of equilibration. Peaks containing **6**–**9** were collected, evaporated in vacuo and obtained as pale white powders (**6**: *t*_R_=18.7 min, yield: 16.39 mg; **9**: *t*_R_=19.3 min, yield: 7.05 mg; **7**: *t*_R_=21.0 min, yield: 39.47 mg; **8**: *t*_R_=23.5 min, yield: 4.45 mg).

**6: 7‐But‐15‐enyl‐6,8‐dihydroxy‐3(R)‐pent‐11‐enylisochroman‐1‐one**: ^1^H NMR (600 MHz, MeCN‐*d*_3_): *δ*=11.45 (s, 1H; 8‐OH),7.63 (s, 1H; 6‐OH), 6.24 (s, 1H; H‐5), 5.48 (overlapped, 1H, H‐11), 5.47 (overlapped, 1H, H‐16), 5.46 (overlapped, 1H, H‐15), 5.42 (overlapped, 1H, H‐12), 4.48 (dddd, ^1^ *J*_C,H_=4.20, 5.17, 7.66, 10.51 Hz, 1H, H‐3), 3.21 (dt, ^1^ *J*_C,H_=1.39, 6.16 Hz, 2H, H‐14), 2.82–2.77 (overlapped, 2H, H‐4), 2.15–2.10 (overlapped, 2H, H‐10), 1.81 (dddd, ^1^ *J*_C,H_=5.88, 7.65, 9.00, 13.60 Hz, 1H, H‐9), 1.70 (dddd, ^1^ *J*_C,H_=5.14, 6.65, 9.28, 14.14 Hz, 1H, H‐9), 1.61 (dd, ^1^ *J*_C,H_=1.18, 5.96 Hz, 3H, H‐17), 1.57 (dq, ^1^ *J*_C,H_=1.43, 1.43, 1.40, 6.21 Hz, 3H, H‐13); ^13^C NMR (150 MHz, MeCN‐*d*_3_): *δ*=171.3 (C‐1), 162.5 (C‐8), 162.0 (C‐6), 140.4 (C‐4a), 130.9 (C‐15), 129.1 (C‐11), 126.7 (C‐16), 126.0 (C‐12), 113.4 (C‐7), 106.8 (C‐5), 102.1(C‐8a), 79.7 (C‐3), 35.2 (C‐9), 33.1 (C‐4), 28.5 (C‐10), 26.0 (C‐14), 18.1 (C‐17), 17.9 (C‐13), *λ*_max_ =228; 272; 304 nm; HRMS (ESI): *m*/*z* calcd for C_18_H_22_O_4_+H^+^: 303.1591 [M+H]^+^; found: 303.1589 (−0.146 ppm); ${\left[\alpha \right]}_{D}^{20}$ −27.6 (*c* 10 in MeOH).

**9: 7‐Butyl‐6,8‐dihydroxy‐3(R)‐pent‐9,11‐dienylisochroman‐1‐one**: ^1^H NMR (600 MHz, MeCN‐*d*_3_): *δ*=11.44 (s, 1H; 8‐OH),7.66 (s, 1H; 6‐OH), 6.36 (dd, ^1^ *J*_C,H_=10.64, 15.19 Hz, 1H, H‐10), 6.27 (s, 1H; H‐5), 6.11 (dddd, ^1^ *J*_C,H_=0.55, 1.66, 10.60, 15.20 Hz, 1H, H‐11), 5.86 (m, 1H, H‐12), 5.69 (dd„ ^1^ *J*_C,H_=6.90; 15.41 Hz, 1H, H‐9), 5.04 (dt, ^1^ *J*_C,H_=6.09, 7.63 Hz, 1H, H‐3), 2.93 (m, 2H, H‐4), 2.57 (m, 2H, H‐14), 1.75 (dd, ^1^ *J*_C,H_=1.63, 6.69 Hz, 3H, H‐13), 1.46 (m, 2H, H‐15), 1.36 (m, 2H, H‐16), 0.92 (t, ^1^ *J*_C,H_=7.34 Hz, 3H, H‐17); ^13^C NMR (150 MHz, MeCN‐*d*_3_): *δ*=171.1 (C‐1), 162.8 (C‐8), 162.2 (C‐6), 139.5 (C‐4a), 134.7 (C‐10), 133.2 (C‐12), 131.1 (C‐11), 127.7 (C‐9), 115.5 (C‐7), 106.9 (C‐5), 102.0 (C‐8a), 80.1 (C‐3), 33.5 (C‐4), 31.7 (C‐15), 23.5 (C‐16), 22.9 (C‐14), 18.3 (C‐13), 14.3 (C‐17), *λ*_max_ =232; 272; 304 nm; HRMS (ESI): *m*/*z* calcd for C_18_H_22_O_4_+H^+^: 303.1591 [M+H]^+^; found: 303.1590 (−0.086 ppm); ${\left[\alpha \right]}_{D}^{20}$ 5 (*c* 4 in MeOH).

**7: 7‐Butyl‐6,8‐dihydroxy‐3(R)‐pent‐11‐enylisochroman‐1‐one**: ^1^H NMR (600 MHz, MeCN‐*d*_3_): *δ*=11.42 (s, 1H; 8‐OH),8.01 (s, 1H; 6‐OH), 6.22 (s, 1H; H‐5), 5.45 (overlapped, 1H, H‐11), 5.45 (overlapped, 1H, H‐12), 4.46 (dddd, ^1^ *J*_C,H_=4.34, 5.14, 7.65, 10.51 Hz, 1H, H‐3), 2.79 (m, 2H, H‐4), 2.53 (m, 2H, H‐14), 2.12 (m, 2H, H‐10), 1.80 (dddd, ^1^ *J*_C,H_=5.87, 7.63, 8.95, 13.50 Hz, 1H, H‐9), 1.68 (dddd, ^1^ *J*_C,H_=5.12, 6.57, 9.32, 14.22 Hz, 1H, H‐9), 1.59 (dd, ^1^ *J*_C,H_=1.32, 2.17 Hz, 3H, H‐13), 1.42 (ddt, ^1^ *J*_C,H_=3.58, 7.68, 9.35 Hz, 2H, H‐15), 1.31 (dq, ^1^ *J*_C,H_=7.16, 7.28, 17.76 Hz, 2H, H‐16), 0.88 (t, ^1^ *J*_C,H_=7.35 Hz, 3H, H‐17); ^13^C NMR (150 MHz, MeCN‐*d*_3_): *δ*=171.4 (C‐1), 162.6 (C‐8), 162.2 (C‐6), 139.9 (C‐4a), 130.9 (C‐11), 126.6 (C‐12), 115.4 (C‐7), 106.8 (C‐5), 101.9 (C‐8a), 79.7 (C‐3), 35.2 (C‐9), 33.1 (C‐4), 31.7 (C‐15), 28.5 (C‐10), 23.5 (C‐16). 22.9 (C‐14), 18.1 (C‐13), 14.3 (C‐17), *λ*_max_=224; 272; 304 nm; HRMS (ESI): *m*/*z* calcd for C_18_H_24_O_4_+H^+^: 305.1747 [M+H]^+^; found: 305.1749 (−0.154 ppm); ${\left[\alpha \right]}_{D}^{20}$ −18 (*c* 0.3 in MeOH).

**8: 7‐Butyl‐6,8‐dihydroxy‐3(R)‐pentylisochroman‐1‐one**: ^1^H NMR (600 MHz, MeCN‐*d*_3_): *δ*=11.49 (s, 1H; 8‐OH),7.65 (s, 1H; 6‐OH), 6.25 (t, ^1^ *J*_C,H_=0.96 Hz, 1H; H‐5), 4.51 (dddd, ^1^ *J*_C,H_=3.86, 5.30, 7.47, 11.29 Hz, 1H, H‐3), 2.84 (m, 2H, H‐4), 2.56 (m, 2H, H‐14), 1.79 (dddd, ^1^ *J*_C,H_=5.29, 7.50, 10.27, 13.91 Hz, 1H, H‐9), 1.68 (ddt, ^1^ *J*_C,H_=5.56, 10.16, 13.83 Hz, 1H, H‐9), 1.50‐1.43 (overlapped, 2H, H‐11), 1.45 (overlapped, 2H, H‐15), 1.34 (overlapped, 2H, H‐10), 1.34 (overlapped, 2H, H‐12), 1.34 (overlapped, 2H, H‐16), 0.92 (overlapped, 3H, H‐17), 1.57 (overlapped, 3H, H‐13); ^13^C NMR (150 MHz, MeCN‐*d*_3_): *δ*=171.5 (C‐1), 162.7 (C‐8), 162.1 (C‐6), 140.2 (C‐4a), 115.3 (C‐7), 106.7 (C‐5), 102.0 (C‐8a), 80.3 (C‐3), 35.3 (C‐9), 33.2 (C‐4), 32.2 (C‐10), 31.7 (C‐15), 25.2 (C‐11), 23.5 (C‐16), 23.2 (C‐12), 22.8 (C‐14), 14.3 (C‐13), 14.2 (C‐17), *λ*_max_ =228; 272; 304 nm; HRMS (ESI): *m*/*z* calcd for C_18_H_26_O_4_+H^+^: 307.1904 [M+H]^+^; found: 307.1902 (−0.146 ppm); ${\left[\alpha \right]}_{D}^{20}$ −°16 (*c* 3 in MeOH).

**Acquisition of MS/MS spectra for compounds 1–9**: Structural elucidation was only possible for compounds **6**–**9**. To prove that compounds **1**–**5** also have a similar structure, MS/MS spectra of compounds **1**–**5** were compared with those of compounds **6**–. MS/MS spectra were generated using parallel reaction monitoring (PRM) and an inclusion with the m/z values and retention times of the nine compounds. The HCD cell was set using a stepped collision energy (CE=20, 45 and 70 eV), to achieve a complex, meaningful fragmentation pattern.

**Cytotoxicity test**: To examine the cytotoxicity, isolated compounds **6**–**9** with purity ≥96 % were dissolved in MeCN at a concentration of 10 mM (stock solution). Human liver cancer cells (HepG2, HB‐8065) (ATCC, Manassas, VA, USA) and human colon cancer cells (HT29, DSMZ Cat# ACC‐299, CVCL_0320) (Leibnitz Institute DSMZ, Braunschweig, Germany) were cultivated as described in Kalinina et al.^[63]^ Cytotoxicity of **6**–**9** was evaluated with resazurin assay. The assay was performed according to previous studies.^[64]^ Briefly, the cells were seeded in 96‐well plates of a suspension of 10 000 cells/well for both cell lines. Cells were incubated for 24 h after the medium was replaced by serum‐free medium and the cells were cultivated for another 24 h. The HepG2 and HT29 cells were treated with compounds **6**–**9** in a concentration range of 0.01–100 μM. Both cell lines were incubated with isolated metabolites for 24 h. After compound exposure, the dye solution of resazurin was added to the cells, followed by incubation for 1.5 h at 37 °C. The fluorescence of reduced resazurin (resorufin) was measured at λ=590 nm with a microplate reader (Infinite M200 PRO, Tecan, Mannendorf, Switzerland). Cytotoxicity tests were repeated three times from three independent passages (n≥9). The data are shown as the mean ± standard deviation (SD). As a positive control, T‐2 toxin, which was previously isolated^[65]^ in a concentration of 10 μM was used. The effect of different concentrations of compounds was investigated by analysis of variance (one‐way ANOVA) and a Tukey post hoc test; * p≤0.05, ** p≤0.01, *** p≤0.001. The IC_50_ values were calculated by log‐linear regression, and the significance indicated refers to the significance level as compared to the solvent‐treated control (MeCN 2 %) calculated with the OriginPro 2016G (64‐bit) Sr2 b9.3.2.303 (SF8T5‐3089‐7901139) (OriginLab Corporation, Northampton, MA, USA).

## Conflict of interest

The authors declare no conflict of interest.

1

## Supporting information

As a service to our authors and readers, this journal provides supporting information supplied by the authors. Such materials are peer reviewed and may be re‐organized for online delivery, but are not copy‐edited or typeset. Technical support issues arising from supporting information (other than missing files) should be addressed to the authors.

Supporting Information

## Data Availability

Deposition Number 2178933 contains the supplementary crystallographic data for this paper. These data are provided free of charge by the joint Cambridge Crystallographic Data Centre and Fachinformationszentrum Karlsruhe Access Structures service.

## References

[1] M. Blum , H.-Y. Chang , S. Chuguransky , T. Grego , S. Kandasaamy , A. Mitchell , G. Nuka , T. Paysan-Lafosse , M. Qureshi , S. Raj , L. Richardson , G. A. Salazar , L. Williams , P. Bork , A. Bridge , J. Gough , D. H. Haft , I. Letunic , A. Marchler-Bauer , H. Mi , D. A. Natale , M. Necci , C. A. Orengo , A. P. Pandurangan , C. Rivoire , C. J. A. Sigrist , I. Sillitoe , N. Thanki , P. D. Thomas , S. C. E. Tosatto , C. H. Wu , A. Bateman , R. D. Finn , Nucleic Acids Res. 2021, 49, D344–D354. 33156333 10.1093/nar/gkaa977 PMC7778928

[2a] J. F. Leslie , B. A. Summerell , in The Fusarium Laboratory Manual, Vol. 6, Blackwell, Iowa, 2006, p. 388;

[2b] T. Aoki , K. O'Donnell , D. M. Geiser , J. Gen. Plant Pathol. 2014, 80, 189–201.

[3a] M. Li , R. Yu , X. Bai , H. Wang , H. Zhang , Nat. Prod. Rep. 2020, 37, 1568–1588; 32785347 10.1039/d0np00038h

[3b] L. Perincherry , J. Lalak-Kańczugowska , Ł. Stępień , Toxin Rev. 2019, 11, 664; 10.3390/toxins11110664 PMC6891594 31739566

[3c] A. Bertero , A. Moretti , L. Spicer , F. Caloni , Toxin Rev. 2018, 10, 244. 10.3390/toxins10060244 PMC6024576 29914090

[4] E.-M. Niehaus , M. Münsterkötter , R. H. Proctor , D. W. Brown , A. Sharon , Y. Idan , L. Oren-Young , C. M. Sieber , O. Novák , A. Pěnčík , D. Tarkowská , K. Hromadová , S. Freeman , M. Maymon , M. Elazar , S. A. Youssef , E. S. M. El-Shabrawy , A. B. A. Shalaby , P. Houterman , N. L. Brock , I. Burkhardt , E. A. Tsavkelova , J. S. Dickschat , P. Galuszka , U. Güldener , B. Tudzynski , Genome Biol. 2016, 8, 3574–3599. 10.1093/gbe/evw259 PMC5203792 28040774

[5] D. K. Chakrabarti , in Mango Malformation, Springer, Netherlands, 2011.

[6] N. P. Keller , Nat. Rev. Microbiol. 2019, 17, 167–180. 30531948 10.1038/s41579-018-0121-1 PMC6381595

[7] B. S. Moore , C. Hertweck , Nat. Prod. Rep. 2002, 19 1, 70–99. 11902441 10.1039/b003939j

[8a] R. J. Cox , T. J. Simpson , Methods Enzymol. 2009, 459, 49–78; 19362635 10.1016/S0076-6879(09)04603-5

[8b] R. J. Cox , E. J. Skellam , K. Williams , in Biosynthesis of Fungal Polyketides, The Mycota Vol. XV: Physiology and Genetics, 2 ^nd^ ed., Springer, 2018, 385–412.

[9] L. Du , L. Lou , Nat. Prod. Rep. 2010, 27, 255–278. 20111804 10.1039/b912037h

[10] L. Hang , M.-C. Tang , C. J. B. Harvey , C. G. Page , J. Li , Y.-S. Hung , N. Liu , M. E. Hillenmeyer , Y. Tang , Angew. Chem. 2017, 129, 9684–9688; 10.1002/anie.201705237 PMC5580239 28679030

[11] U. Rix , C. Fischer , L. L. Remsing , J. R. Rohr , Nat. Prod. Rep. 2002, 19, 542–580. 12430723 10.1039/b103920m

[12] N. P. Keller , T. M. Hohn , Fungal Genet. Biol. 1997, 21 1, 17–29. 9126615

[13] E. K. Shwab , N. P. Keller , Mycol. Res. 2008, 112, 225–230. 18280128 10.1016/j.mycres.2007.08.021

[14] A. K. Atanasoff-Kardjalieff , L. Studt , Toxin Rev. 2022, 14, 96. 10.3390/toxins14020096 PMC8880415 35202124

[15] J.-i. Nakayama , J. C. Rice , B. D. Strahl , C. D. Allis , S. I. S. Grewal , Science 2001, 292, 110–113. 11283354 10.1126/science.1060118

[16] H. Tamaru , E. U. Selker , Nature 2001, 414, 277–283. 11713521 10.1038/35104508

[17] A. K. Atanasoff-Kardjalieff , F. Lünne , S. Kalinina , J. Strauss , H.-U. Humpf , L. Studt , Front. Fungal Biol. 2021, 2:671796 . 10.3389/ffunb.2021.671796 PMC10512364 37744112

[18a] M. Möller , K. Schotanus , J. L. Soyer , J. Haueisen , K. Happ , M. Stralucke , P. Happel , K. M. Smith , L. R. Connolly , M. Freitag , E. H. Stukenbrock , PLoS Genet. 2019, 15, e1008093; 31009462 10.1371/journal.pgen.1008093 PMC6510446

[18b] T. Chujo , B. Scott , Mol. Microbiol. 2014, 92, 413–434. 24571357 10.1111/mmi.12567

[19] Y. Suzuki , Agric. Biol. Chem. 1970, 34, 760–766.

[20a] P. Wiemann , S. Albermann , E.-M. Niehaus , L. Studt , K. W. Von Bargen , N. L. Brock , H.-U. Humpf , J. S. Dickschat , B. Tudzynski , PLoS ONE 2012, 7, e37519; 22662164 10.1371/journal.pone.0037519 PMC3360786

[20b] C. Neville , A. Murphy , K. Kavanagh , S. Doyle , ChemBioChem 2005, 6, 679–685; 15719355 10.1002/cbic.200400147

[20c] R. Horbach , A. Graf , F. Weihmann , L. Antelo , S. Mathea , J. C. Liermann , T. Opatz , E. Thines , J. S. Aguirre , H. B. Deising , Plant Cell 2009, 21, 3379–3396. 19880801 10.1105/tpc.108.064188 PMC2782280

[21] P. Wiemann , C. M. K. Sieber , K. W. Von Bargen , L. Studt , E.-M. Niehaus , J. J. Espino , K. Huß , C. B. Michielse , S. Albermann , D. Wagner , S. V. Bergner , L. R. Connolly , A. Fischer , G. Reuter , K. Kleigrewe , T. Bald , B. D. Wingfield , R. Ophir , S. Freeman , M. Hippler , K. M. Smith , D. W. Brown , R. H. Proctor , M. Münsterkötter , M. Freitag , H.-U. Humpf , U. Güldener , B. Tudzynski , PLoS Pathog. 2013, 9, e1003475. 23825955 10.1371/journal.ppat.1003475 PMC3694855

[22] A. Pfannmüller , J. Leufken , L. Studt , C. B. Michielse , C. M. K. Sieber , U. Güldener , S. Hawat , M. Hippler , C. Fufezan , B. Tudzynski , PLoS One 2017, 12, e0176194. 28441411 10.1371/journal.pone.0176194 PMC5404775

[23] M. Mihlan , V. Homann , T. W. Liu , B. Tudzynski , Mol. Microbiol. 2003, 47, 975–991. 12581353 10.1046/j.1365-2958.2003.03326.x

[24] C. B. Michielse , A. Pfannmüller , M. Macios , P. Rengers , A. Dzikowska , B. Tudzynski , Mol. Microbiol. 2014, 91, 472–493. 24286256 10.1111/mmi.12472

[25] S. Altschul , Nucleic Acids Res. 1997, 25, 3389–3402. 9254694 10.1093/nar/25.17.3389 PMC146917

[26] C. Yu , N. Zavaljevski , V. Desai , J. Reifman , Nucleic Acids Res. 2011, 39, e88–e88. 21572104 10.1093/nar/gkr308 PMC3141274

[27] E.-M. Niehaus , H.-K. Kim , M. Münsterkötter , S. Janevska , B. Arndt , S. A. Kalinina , P. M. Houterman , I.-P. Ahn , I. Alberti , S. Tonti , D.-W. Kim , C. M. K. Sieber , H.-U. Humpf , S.-H. Yun , U. Güldener , B. Tudzynski , PLoS Pathog. 2017, 13, e1006670. 29073267 10.1371/journal.ppat.1006670 PMC5675463

[28] M. Sabatini , S. Comba , S. Altabe , A. I. Recio-Balsells , G. R. Labadie , E. Takano , H. Gramajo , A. Arabolaza , FEBS J. 2018, 285, 4494–4511. 30300504 10.1111/febs.14675 PMC6334511

[29] P. Kongsaeree , S. Prabpai , N. Sriubolmas , C. Vongvein , S. Wiyakrutta , J. Nat. Prod. 2003, 66, 709–711. 12762815 10.1021/np0205598

[30] Y. Cai , L. Rao , Y. Zou , Org. Lett. 2021, 23, 2337–2341. 33688736 10.1021/acs.orglett.1c00458

[31] D.-C. Kim , T. H. Quang , N. T. T. Ngan , C.-S. Yoon , J. H. Sohn , J. H. Yim , Y. Feng , Y. Che , Y.-C. Kim , H. Oh , J. Nat. Prod. 2015, 78, 2948–2955. 26651366 10.1021/acs.jnatprod.5b00614

[32] X. Pang , X. Lin , J. Wang , R. Liang , Y. Tian , L. Salendra , X. Luo , X. Zhou , B. Yang , Z. Tu , Y. Liu , Steroids 2018, 129, 41–46. 29223616 10.1016/j.steroids.2017.12.001

[33] P. Reveglia , M. Masi , A. Evidente , Biomol. Eng. 2020, 10, 772. 10.3390/biom10050772 PMC7277180 32429259

[34] X. Zhu , F. Yu , X.-C. Li , L. Du , J. Am. Chem. Soc. 2007, 129, 36–37. 17199276 10.1021/ja0672122

[35] M. A. Skiba , A. P. Sikkema , W. D. Fiers , W. H. Gerwick , D. H. Sherman , C. C. Aldrich , J. L. Smith , ACS Chem. Biol. 2016, 11, 3319–3327. 27723289 10.1021/acschembio.6b00759 PMC5224524

[36] P. A. Storm , P. Pal , C. R. Huitt-Roehl , C. A. Townsend , ACS Chem. Biol. 2018, 13, 3043–3048. 30350943 10.1021/acschembio.8b00429 PMC6855380

[37] V. Hampl , Sci. Pharm. 2011, 79, 21–30. 21617770 10.3797/scipharm.1011-10 PMC3097510

[38] Y.-F. Huang , L.-H. Li , L. Tian , L. Qiao , H.-M. Hua , Y.-H. Pei , J. Antibiot. 2006, 59, 355–357. 10.1038/ja.2006.50 16915820

[39] A. Droce , W. Saei , S. Jørgensen , R. Wimmer , H. Giese , R. Wollenberg , T. Sondergaard , J. Sørensen , Molecules 2016, 21, 1710. 27983606 10.3390/molecules21121710 PMC6274466

[40a] B. D. Ames , C. Nguyen , J. Bruegger , P. Smith , W. Xu , S. Ma , E. Wong , S. Wong , X. Xie , J. W.-H. Li , J. C. Vederas , Y. Tang , S.-C. Tsai , Proc. Natl. Acad. Sci. USA 2012, 109, 11144–11149; 22733743 10.1073/pnas.1113029109 PMC3396468

[40b] Y.-P. Chen , C.-P. Tseng , L.-L. Liaw , C.-L. Wang , I.-C. Chen , W.-J. Wu , M.-D. Wu , G.-F. Yuan , J. Agric. Food Chem. 2008, 56, 5639–5646; 18578535 10.1021/jf800595k

[40c] Y. Abe , T. Suzuki , C. Ono , K. Iwamoto , M. Hosobuchi , H. Yoshikawa , Mol. Genet. Genomics 2002, 267, 636–646; 12172803 10.1007/s00438-002-0697-y

[40d] K. L. Eley , L. M. Halo , Z. Song , H. Powles , R. J. Cox , A. M. Bailey , C. M. Lazarus , T. J. Simpson , ChemBioChem 2007, 8, 289–297; 17216664 10.1002/cbic.200600398

[40e] M. N. Heneghan , A. A. Yakasai , K. Williams , K. A. Kadir , Z. Wasil , W. Bakeer , K. M. Fisch , A. M. Bailey , T. J. Simpson , R. J. Cox , C. M. Lazarus , Chem. Sci. 2011, 2, 972;

[40f] K. Qiao , Y.-H. Chooi , Y. Tang , Metab. Eng. 2011, 13, 723–732; 21983160 10.1016/j.ymben.2011.09.008 PMC3254600

[40g] T. B. Kakule , D. Sardar , Z. Lin , E. W. Schmidt , ACS Chem. Biol. 2013, 8, 1549–1557; 23614392 10.1021/cb400159f

[40h] J. W. Sims , J. P. Fillmore , D. D. Warner , E. W. Schmidt , Chem. Comm. 2005, 186–188. 10.1039/b413523g 15724180

[41] J. Kennedy , K. Auclair , S. G. Kendrew , C. Park , J. C. Vederas , C. R. Hutchinson , Science 1999, 284, 1368–1372. 10334994 10.1126/science.284.5418.1368

[42a] W. Xu , X. Cai , M. E. Jung , Y. Tang , Am. Chem. J. 2010, 132, 13604–13607; 10.1021/ja107084d PMC2950873 20828130

[42b] H. Tao , T. Mori , X. Wei , Y. Matsuda , I. Abe , Angew. Chem. Int. Ed. 2021, 60, 8851–8858. 10.1002/anie.202016525 33480463

[43] M. L. Adrover-Castellano , J. J. Schmidt , D. H. Sherman , ChemCatChem 2021, 13, 2095–2116. 34335987 10.1002/cctc.202001886 PMC8320681

[44] A. O. Zabala , Y.-H. Chooi , M. S. Choi , H.-C. Lin , Y. Tang , ACS Chem. Biol. 2014, 9, 1576–1586. 24845309 10.1021/cb500284t PMC4215887

[45] W. Xu , Y.-H. Chooi , J. W. Choi , S. Li , J. C. Vederas , N. A. Da Silva , Y. Tang , Angew. Chem. Int. Ed. Engl. 2013, 52, 6472–6475. 23653178 10.1002/anie.201302406 PMC3844545

[46] W.-G. Wang , H. Wang , L.-Q. Du , M. Li , L. Chen , J. Yu , G.-G. Cheng , M.-T. Zhan , Q.-F. Hu , L. Zhang , M. Yao , Y. Matsuda , J. Am. Chem. Soc. 2020, 142, 8464–8472. 32275405 10.1021/jacs.0c02337

[47] G. Pontecorvo , J. A. Roper , L. M. Hemmons , K. D. Macdonald , A. Bufton , Adv. Genet. 1953, 5, 141–238. 13040135 10.1016/s0065-2660(08)60408-3

[48] T. A. Geissman , A. J. Verbiscar , B. O. Phinney , G. M. Cragg , Phytochemistry 1966, 5, 933–947.

[49] M. A. Darken , A. L. Jensen , P. Shu , Appl. Microbiol. 1959, 7, 301–303. 13814121 10.1128/am.7.5.301-303.1959 PMC1057525

[50] C. Staben , B. Jensen , M. Singer , J. Pollock , M. Schechtman , J. Kinsey , E. Selker , Fungal Genet. Rep. 1989, 36.

[51] J. Schumacher , Fungal Genet. Biol. 2012, 49, 483–497. 22503771 10.1016/j.fgb.2012.03.005

[52] T. W. Christianson , R. S. Sikorski , M. Dante , J. H. Shero , P. Hieter , Gene 1992, 110, 119–122. 1544568 10.1016/0378-1119(92)90454-w

[53] F. Winston , C. Dollard , S. L. Ricuperohovasse , Yeast 1995, 11, 53–55. 7762301 10.1002/yea.320110107

[54] B. Tudzynski , V. Homann , B. Feng , G. A. Marzluf , Mol. Gen. Genet. 1999, 261, 106–114. 10071216 10.1007/s004380050947

[55] R. S. Goswami , Methods Mol. Biol. 2012, 835, 255–269. 22183659 10.1007/978-1-61779-501-5_16

[56] J. L. Cenis , Nucleic Acids Res. 1992, 20, 2380. 1594460 10.1093/nar/20.9.2380 PMC312363

[57] M. W. Pfaffl , Nucleic Acids Res. 2001, 29, 45e–45. 10.1093/nar/29.9.e45 PMC55695 11328886

[58] C. A. Smith , E. J. Want , G. O′Maille , R. Abagyan , G. Siuzdak , Anal. Chem. 2006, 78, 779–787. 16448051 10.1021/ac051437y

[59] Bruker AXS Inc., Madison, Wisconsin, USA., **2019**.

[60] G. M. Sheldrick , Acta Crystallogr. Sect. A 2015, 71, 3–8. 10.1107/S2053273314026370 PMC4283466 25537383

[61] G. M. Sheldrick , Acta Crystallogr. Sect. C 2015, 71, 3–8. 10.1107/S2053273314026370 PMC4283466 25537383

[62] Bruker AXS Inc., Madison, Wisconsin, USA, **1998**.

[63] S. A. Kalinina , A. Jagels , S. Hickert , L. M. Mauriz Marques , B. Cramer , H.-U. Humpf , J. Agric. Food Chem. 2018, 66, 1264–1269. 29338236 10.1021/acs.jafc.7b06001

[64a] T. Mosmann , J. Immunol. Methods 1983, 65, 55–63; 6606682 10.1016/0022-1759(83)90303-4

[64b] M. M. Nociari , A. Shalev , P. Benias , C. Russo , J. Immunol. Methods 1998, 213, 157–167; 9692848 10.1016/s0022-1759(98)00028-3

[64c] J. O′Brien , I. Wilson , T. Orton , F. Pognan , Eur. J. Biochem. 2000, 267, 5421–5426. 10951200 10.1046/j.1432-1327.2000.01606.x

[65] M. Beyer , I. Ferse , H.-U. Humpf , Mycotoxin Res. 2009, 25, 41–52. 23604935 10.1007/s12550-009-0006-2

