# Supplementary material for: Biosynthesis of the Isocoumarin Derivatives Fusamarins is Mediated by the PKS8 Gene Cluster in Fusarium
Source: Chembiochem. 2022 Oct 18;24(6):e202200342. doi: 10.1002/cbic.202200342 (PMC10947347; doi:10.1002/cbic.202200342)
Supplement: Supplementary file 1 — Supporting Information [file CBIC-24-0-s001.pdf]

ChemBioChem

Supporting Information

Biosynthesis of the Isocoumarin Derivatives Fusamarins is Mediated by the PKS8 Gene Cluster in *Fusarium*

Anna K. Atanasoff-Kardjalieff, Bernhard Seidl, Katharina Steinert, Constantin G. Daniliuc, Rainer Schuhmacher, Hans-Ulrich Humpf, Svetlana Kalinina, and Lena Studt-Reinhold*

Table of contents

FIGURE S1 VENN DIAGRAMS ILLUSTRATING THE RESULTS OF THE UNTARGETED METABOLITE PROFILE COMPARISONS OF THE RESPECTIVE CULTURE SUPERNATANT SAMPLES. 3
FIGURE S2: METABOLITE SPECTRUM OF FMWT COMPARED TO Δ*FMPPT1* AND Δ*FMPKS8*. 4
FIGURE S3: PHENOTYPIC APPEARANCE OF *F. MANGIFERAE* WILD TYPE (FMWT) AND THE FMH3K9R MUTANT ON V8 MEDIUM. 5
FIGURE S4: GENE EXPRESSION ANALYSIS OF FMPKS8 IN *FUSARIUM MANGIFERAE* (FMWT) IN Δ*FMKMT1* AND FMH3K9R MUTANT STRAINS. 6
FIGURE S5: SEMI-QUANTITATIVE PCR OF *FVPKS8* GENE EXPRESSION IN *FUSARIUM VERTICILLIOIDES* (FVWT). 7
FIGURE S6 COMPARISON AND VERIFICATION OF PKS8-RELATED COMPOUND PRODUCTION BY *F. VERTICILLIOIDES* (FVWT) AND *F. MANGIFERAE* (FMWT). 8
FIGURE S7 CHEMICAL ANALYSIS OF FMPKS8 TARGET COMPOUNDS AFTER THE DELETION OF CLUSTER BORDER GENES. 9
FIGURE S8 ^{1}H NMR SPECTRUM (600 MHZ, MECN-D_3) OF COMPOUND **6**. 10
FIGURE S9 ^{13}C NMR SPECTRUM (150 MHZ, MECN-D_3) OF COMPOUND **6**. 11
FIGURE S10 ^{1}H, ^{1}H COSY SPECTRUM (MECN-D_3) OF COMPOUND **6**. 12
FIGURE S11 HSQC SPECTRUM (MECN-D_3) OF COMPOUND **6**. 13
FIGURE S12 HMBC SPECTRUM (MECN-D_3) OF COMPOUND **6**. 14
FIGURE S13 ^{1}H NMR SPECTRUM (600 MHZ, MECN-D_3) OF COMPOUND **9**. 15
FIGURE S14 ^{13}C NMR SPECTRUM (150 MHZ, MECN-D_3) OF COMPOUND **9**. 16
FIGURE S15 ^{1}H, ^{1}H COSY SPECTRUM (MECN-D_3) OF COMPOUND **9**. 17
FIGURE S16 HSQC SPECTRUM (MECN-D_3) OF COMPOUND **9**. 18
FIGURE S17 HMBC SPECTRUM (MECN-D_3) OF COMPOUND **9**. 19
FIGURE S18 ^{1}H NMR SPECTRUM (600 MHZ, MECN-D_3) OF COMPOUND **7**. 20
FIGURE S19 ^{13}C NMR SPECTRUM (150 MHZ, MECN-D_3) OF COMPOUND **7**. 21
FIGURE S20 ^{1}H, ^{1}H COSY SPECTRUM (MECN-D_3) OF COMPOUND **7**. 22
FIGURE S21 HSQC SPECTRUM (MECN-D_3) OF COMPOUND **7**. 23
FIGURE S22 HMBC SPECTRUM (MECN-D_3) OF COMPOUND **7**. 24
FIGURE S23: ^{1}H NMR SPECTRUM (600 MHZ, MECN-D_3) OF COMPOUND **8**. 25
FIGURE S24 ^{13}C NMR SPECTRUM (150 MHZ, MECN-D_3) OF COMPOUND **8**. 26
FIGURE S25 ^{1}H, ^{1}H COSY SPECTRUM (MECN-D_3) OF COMPOUND **8**. 27
FIGURE S26 HSQC SPECTRUM (MECN-D_3) OF COMPOUND **8**. 28
FIGURE S27 HMBC SPECTRUM (MECN-D_3) OF COMPOUND **8**. 29
FIGURE S28 HRMS SPECTRUM OF COMPOUND **6**. 30
FIGURE S29 HRMS SPECTRUM OF COMPOUND **9**. 30
FIGURE S30 HRMS SPECTRUM ON COMPOUND **7**. 31
FIGURE S31 HRMS SPECTRUM OF COMPOUND **8**. 31
FIGURE S32 EXCERPT OF THE PACKING DIAGRAM. 32
FIGURE S33 FULL MS/MS SPECTRUM OF COMPOUND 1. 33
FIGURE S34 FULL MS/MS SPECTRUM OF COMPOUND 2. 34
FIGURE S35 FULL MS/MS SPECTRUM OF COMPOUND 3. 35
FIGURE S36 FULL MS/MS SPECTRUM OF COMPOUND 4. 36
FIGURE S37 FULL MS/MS SPECTRUM OF COMPOUND 5. 37
FIGURE S38 FULL MS/MS SPECTRUM OF COMPOUND 6. 38
FIGURE S39 FULL MS/MS SPECTRUM OF COMPOUND 7. 39
FIGURE S40 FULL MS/MS SPECTRUM OF COMPOUND 8. 40
FIGURE S41 FULL MS/MS SPECTRUM OF COMPOUND 9. 41
FIGURE S42 EXPRESSIONAL ANALYSIS OF PUTATIVE CLUSTER GENES INVOLVED IN FUSAMARIN BIOSYNTHESIS. 42
FIGURE S43 DELETION STRATEGY AND VERIFICATION OF Δ*FMAN_15219* DELETION BY DIAGNOSTIC PCR. 43

FIGURE S44 DELETION STRATEGY AND VERIFICATION OF Δ*FMAN_15220* DELETION BY DIAGNOSTIC PCR. 44
FIGURE S45 DELETION STRATEGY AND VERIFICATION OF Δ*FMAN_15221* DELETION BY DIAGNOSTIC PCR. 45
FIGURE S46 DELETION STRATEGY AND VERIFICATION OF Δ*FMAN_15222* DELETION BY DIAGNOSTIC PCR. 46
FIGURE S47 DELETION STRATEGY AND VERIFICATION OF Δ*FMAN_15224* DELETION BY DIAGNOSTIC PCR. 47
FIGURE S48 DELETION STRATEGY AND VERIFICATION OF Δ*FVPKS8* PARTIAL GENE DELETION BY DIAGNOSTIC PCR. 48
FIGURE S49 OVEREXPRESSION STRATEGY AND VERIFICATION OF OE*::FMAN_15220*. 49

TABLE S1: METABOLOMIC COMPARISON OF DIFFERENT STRAINS. 50
TABLE S2: OVERVIEW OF PRIMERS USED IN THIS WORK. 51
TABLE S3 NON-COVALENT INTERMOLECULAR INTERACTIONS IN COMPOUND **6** (Å AND DEG).. 57
TABLE S4: TABLE OF FRAGMENTED IONS OF COMPOUNDS **6-9** 58
TABLE S5: TABLE OF NEUTRAL LOSSES OF NEUTRAL LOSSES OF COMPOUNDS **6-9** 58
TABLE S6: TABLE OF FRAGMENTED IONS OF COMPOUND **1.** 58
TABLE S7: TABLE OF FRAGMENTED IONS OF COMPOUND **2.** 58
TABLE S8: TABLE OF FRAGMENTED IONS OF COMPOUND **3.** 58
TABLE S9: TABLE OF FRAGMENTED IONS OF COMPOUND **4.** 58
TABLE S10: TABLE OF FRAGMENTED IONS OF COMPOUND **5.** 58

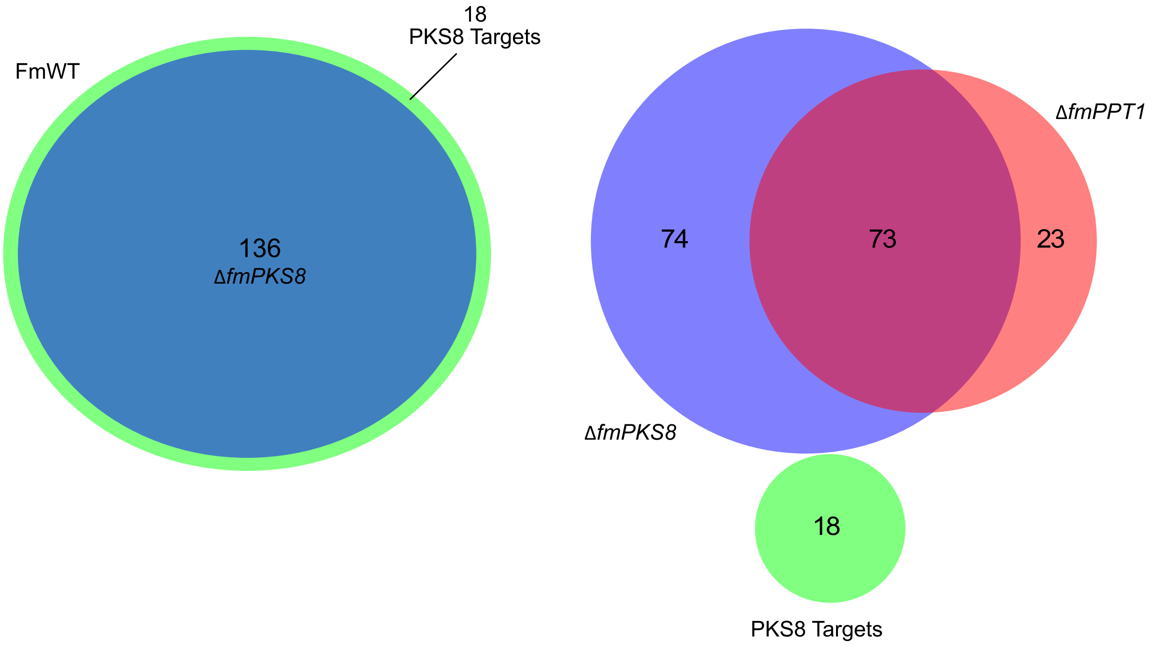

Figure S1 Venn diagrams illustrating the results of the untargeted metabolite profile comparisons of the respective culture supernatant samples. (A) Comparison of the detected metabolites in FmWT and ΔfmPKS8. 136 metabolites were detected in both strains. Additional 18 metabolites were found, representing the possible PKS8 Targets. (B) Comparison of all detected metabolites in Δ*fmPPT1 and* Δ*fmPKS8*. 73 metabolites were found in both genotypes, further 74 metabolites were exclusively found in the Δ*fmPKS8* samples whereas another 23 metabolites were only found in the Δ*fmPPT1* strain. Additionally, the 18 possible PKS8 Target metabolites are indicated in green. None of them were found in the Δ*fmPKS8* and Δ*fmPPT1* strain samples.

Metabolite spectrum of FmWT and Δ*fmPPT1* and Δ*fmPKS8* deletion strains

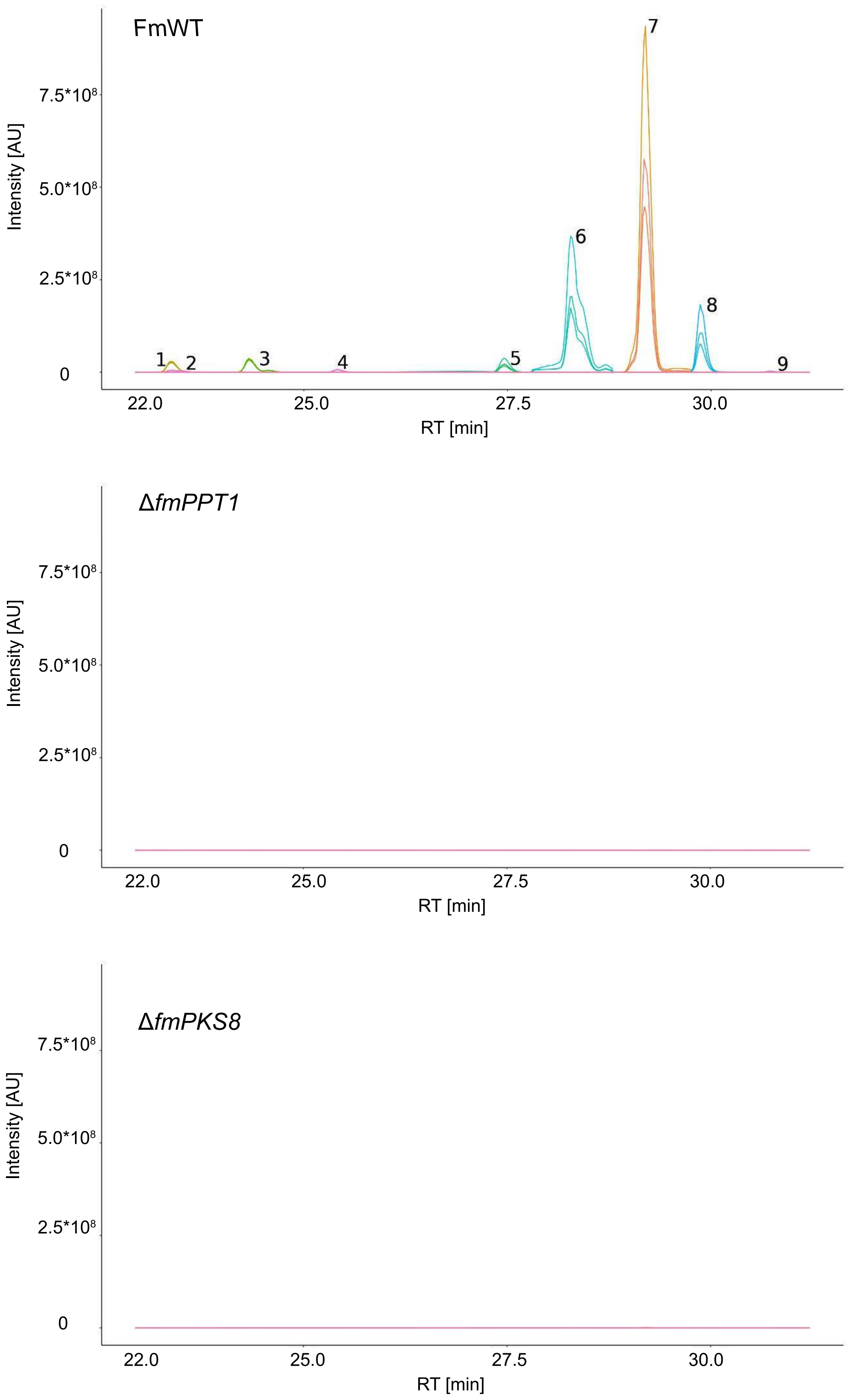

Figure S2: Metabolite spectrum of FmWT compared to Δ*fmPPT1* and Δ*fmPKS8.* Extracted ion chromatograms (EICs) of all nine PKS8 associated metabolite features (**1**-**9**), found in the LC-HRMS data. Numbers shown in the diagram correspond to the target numbers **1-9** from Table 1. The EICs are depicted for three biological replicates for *F. mangiferae* wild-type (FmWT) and the Δ*fmPPT1* and Δ*fmPKS8* deletion strains.

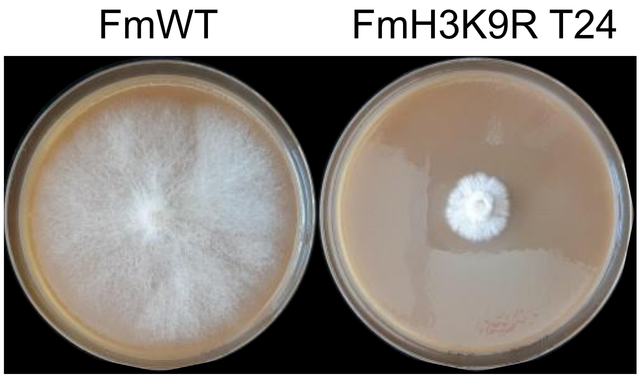

Figure S3: Phenotypic appearance of *F. mangiferae* wild type (FmWT) and the FmH3K9R mutant on V8 medium. A V8 plate was inoculated with a 5 mm agar plug of FmWT and FmH3K9R mutant strain and incubated at 30 °C for 7 days in the dark. Loss of H3K9me3 and H3K9ac lead to severe growth retardations compared to FmWT.

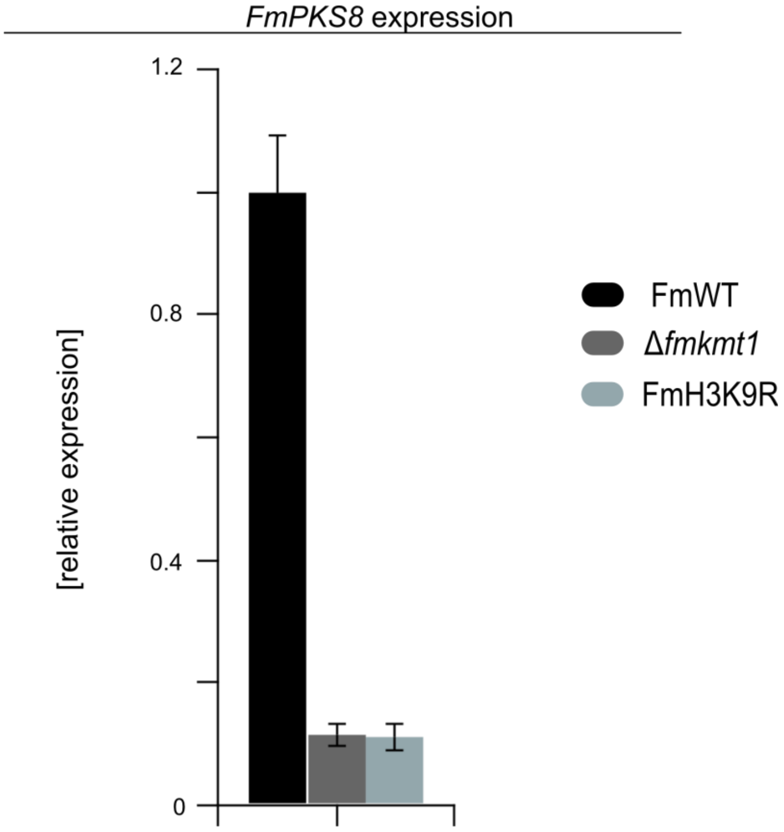

Figure S4: Gene expression analysis of FmPKS8 in *Fusarium mangiferae* (FmWT) in ∆*fmkmt1* and FmH3K9R mutant strains. FmWT, ∆*fmkmt1* and FmH3K9R were cultivated for 4 days in FmPKS8-inducing conditions**.** FmPKS8 gene expression was measured after RNA extraction with subsequent cDNA synthesis by RT-qPCR. For data normalization, the house-keeping genes actin, GPD and β-tubulin were used. Primers are listed in Supplementary Table 2. The FmWT gene expression was arbitrarily set to 1. Mean values and standard deviations are shown.

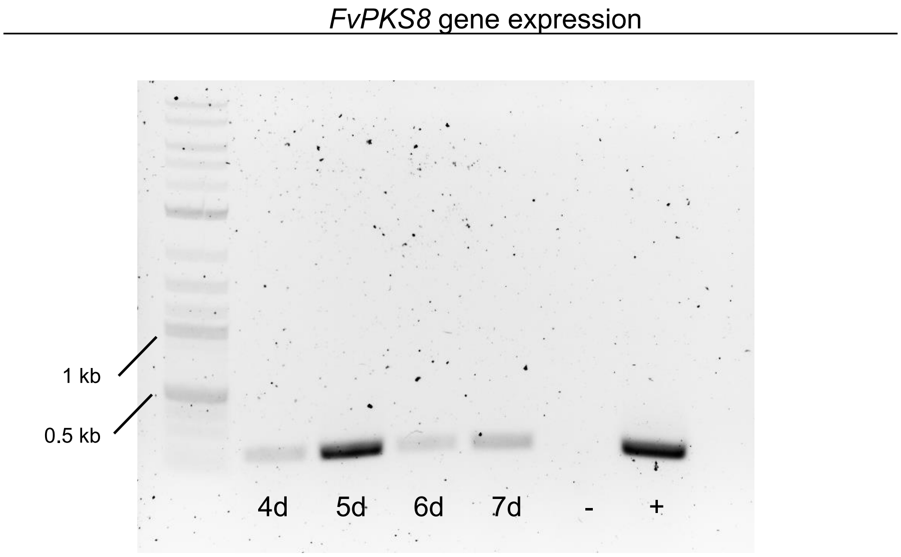

Figure S5: Semi-quantitative PCR of *FvPKS8* gene expression in *Fusarium verticillioides* (FvWT). FvWT was cultivated four to seven days in PKS8-inducing conditions (6 mM $NaNO_3$). RNA was reverse transcribed to cDNA and used as template for semi-quantitative PCR. FvWT gDNA served as positive (+) control, while sterile IonEx was used as negative control. The 1 kb Plus DNA ladder (NEB) was used as a size marker. All used primers are listed in Supplementary Table 2.

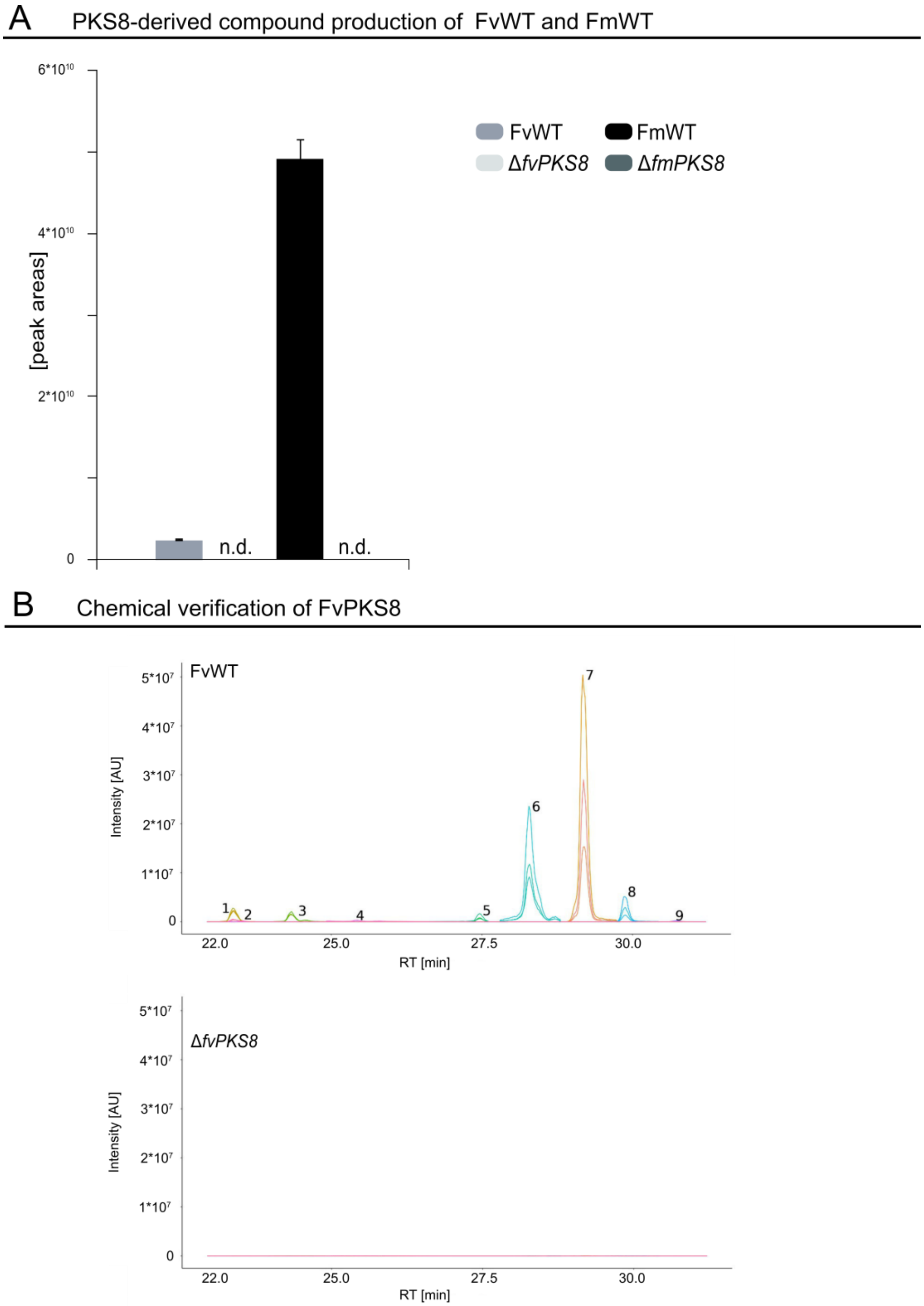

Figure S6 Comparison and verification of PKS8-related compound production by *F. verticillioides* (FvWT) and *F. mangiferae* (FmWT). (A) Comparison of FvWT and FmWT PKS8 production levels, including the respective PKS8 deletion strains. Measurement of fungal cultures revealed that the respective *PKS8* deletion strains did not produce PKS8 related compounds **1**-**9** anymore. FvW and FmWT produced **1**-**9**, albeit FvWT in lower amounts. Fungal strains were cultivated in triplicates in *PKS8*-inducing conditions for 7 days in the dark. Mean values and standard deviations are shown in diagram. n.d., not detected in the supernatant. (B) Chromatograms of FvWT and *FvPKS8* deletion strain under PKS8-inducing conditions. For FvWT target compounds **1**-**9** are detected, while for the Δ*fvPKS8* strain biosynthesis of **1**-**9** was abolished. Strains were cultivated for 7 days in PKS8-inducing media in the dark. Fungal strains were cultivated in triplicates.

Chemical anaylsis of PKS8-target compounds after deletion of putative cluster border genes

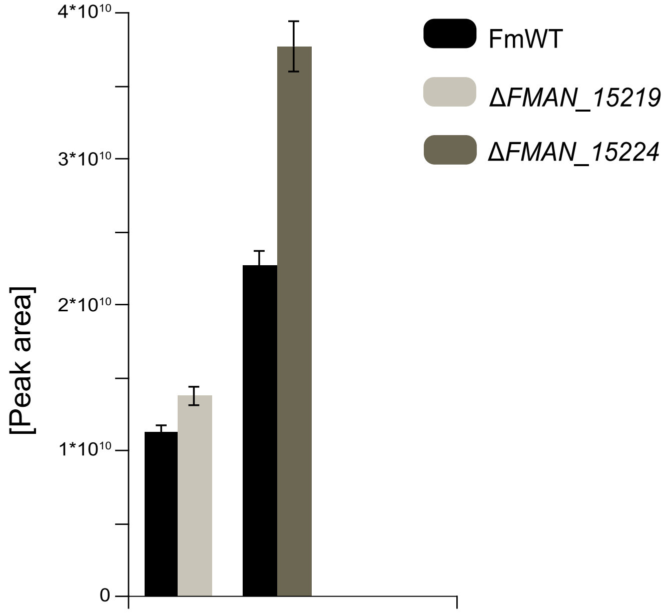

Figure S7 Chemical analysis of FmPKS8 target compounds after the deletion of cluster border genes. Chemical analysis of the cluster border gene deletion mutants Δ*FMAN_15219* and Δ*FMAN_15224*. *F. mangiferae* (FmWT), *FMAN_15219* and *FMAN_15224* deletion strains were cultivated in triplicates in *PKS8*-inducing conditions for seven days in the dark. Measurement of fungal culture filtrates via HPLC-HRMS revealed that loss of both, FMAN_15219 and FMAN_15224 did not influence the production of **1**-**9**. Mean values and standard deviations are shown in diagram.

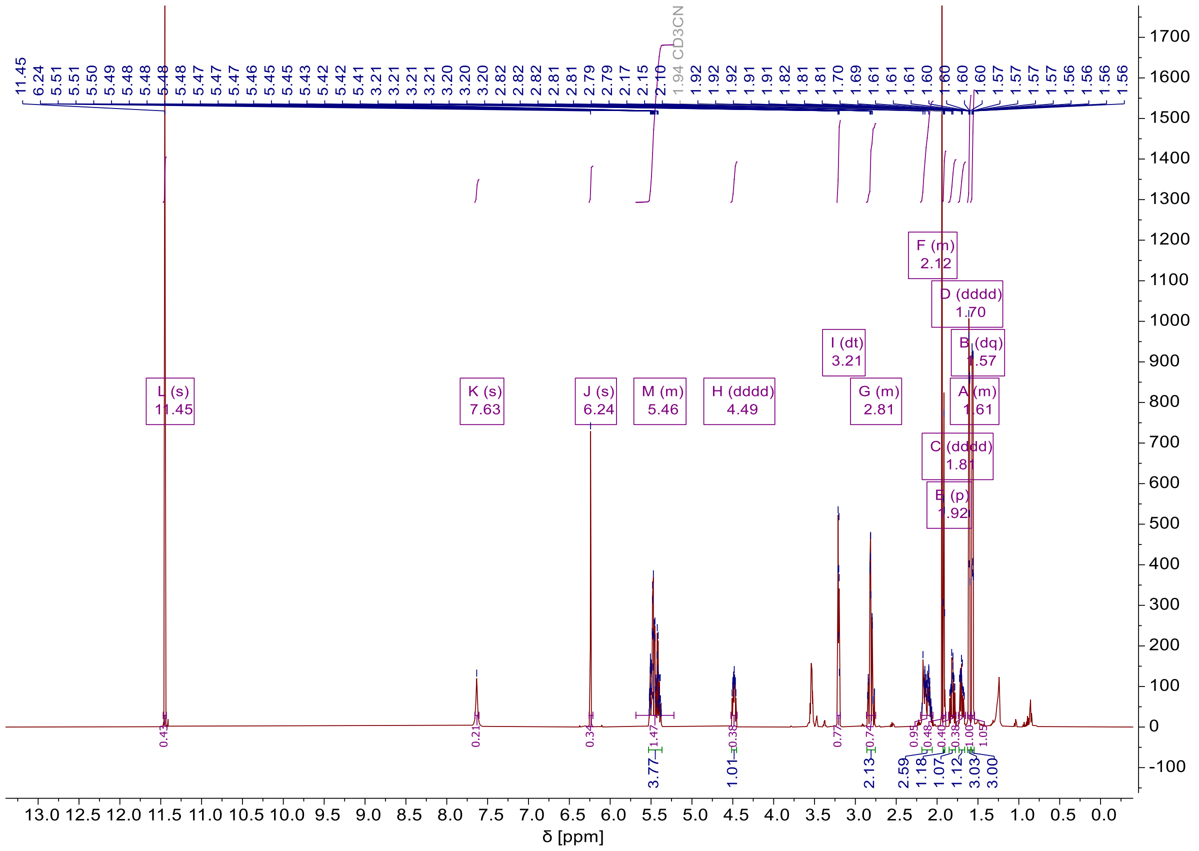

Figure S8 ^{1}H NMR spectrum (600 MHz, MeCN-d_3) of compound **6**.

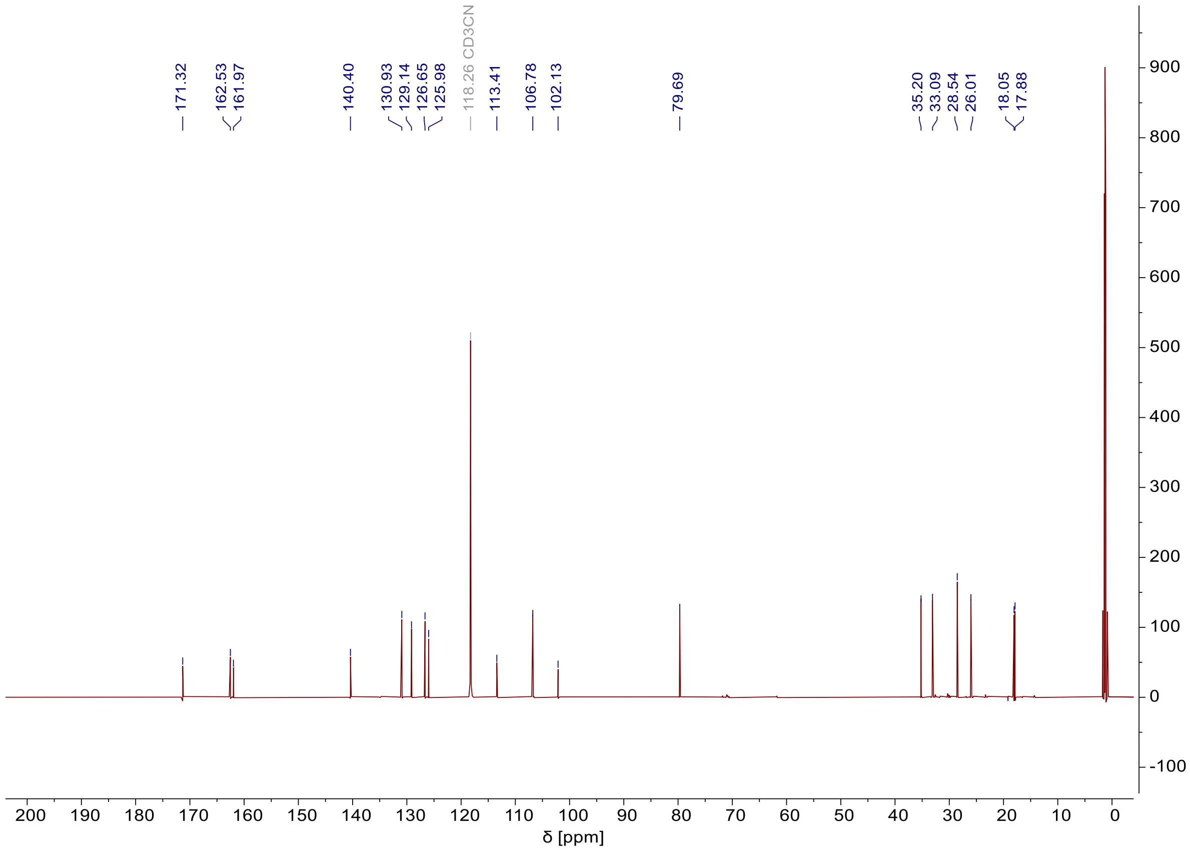

Figure S9 ^{13}C NMR spectrum (150 MHz, MeCN-d_3) of compound **6**.

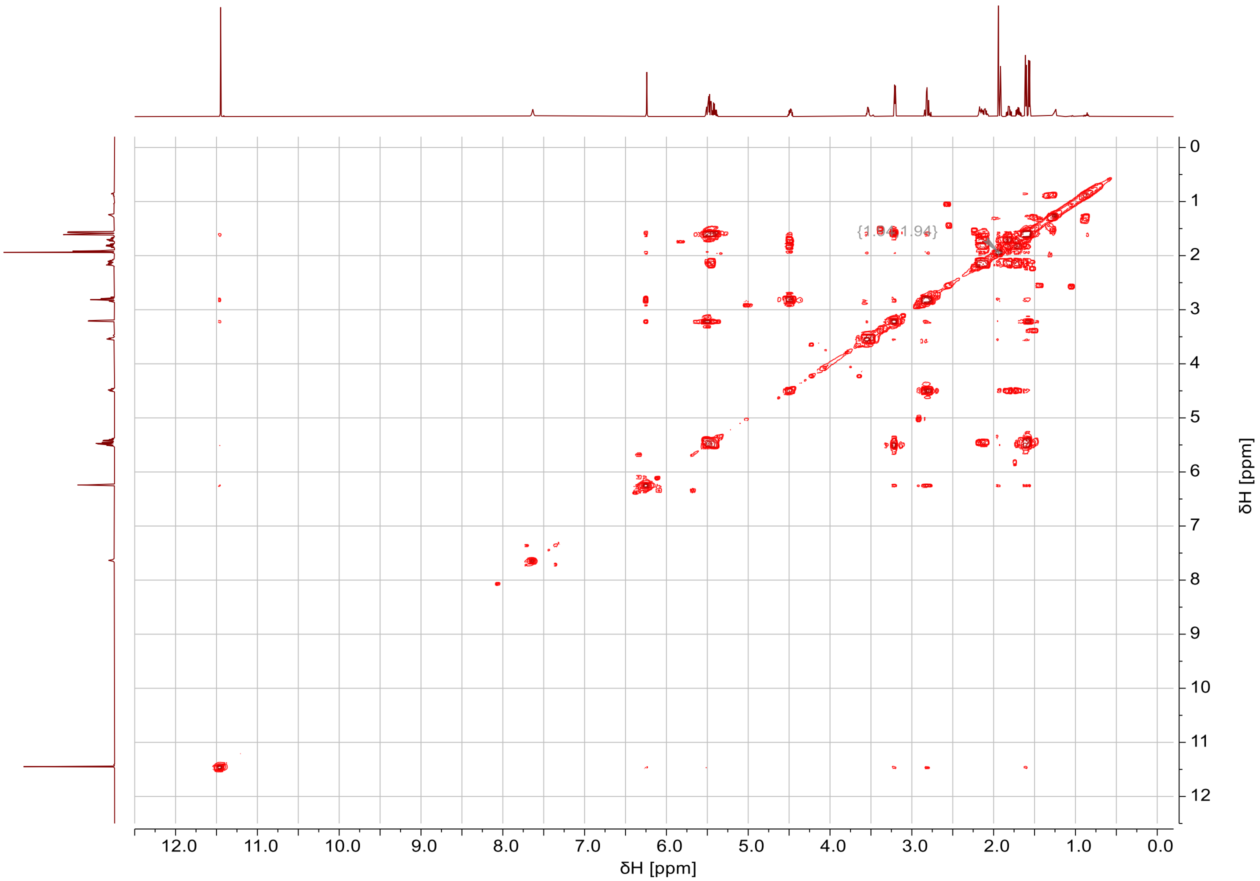

Figure S10 ^{1}H, ^{1}H COSY spectrum (MeCN-*d*$_3$) of compound **6**.

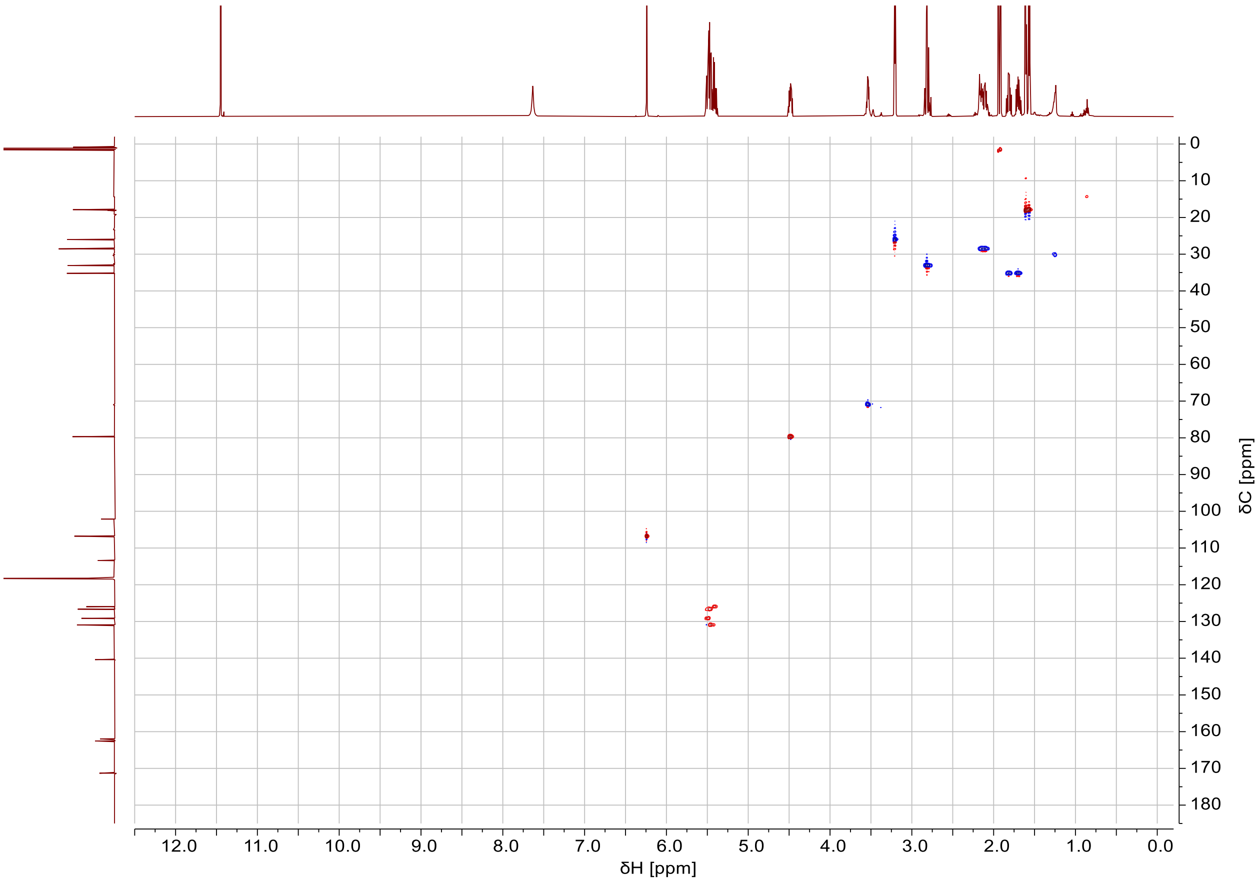

Figure S11 HSQC spectrum (MeCN-d_3) of compound **6**.

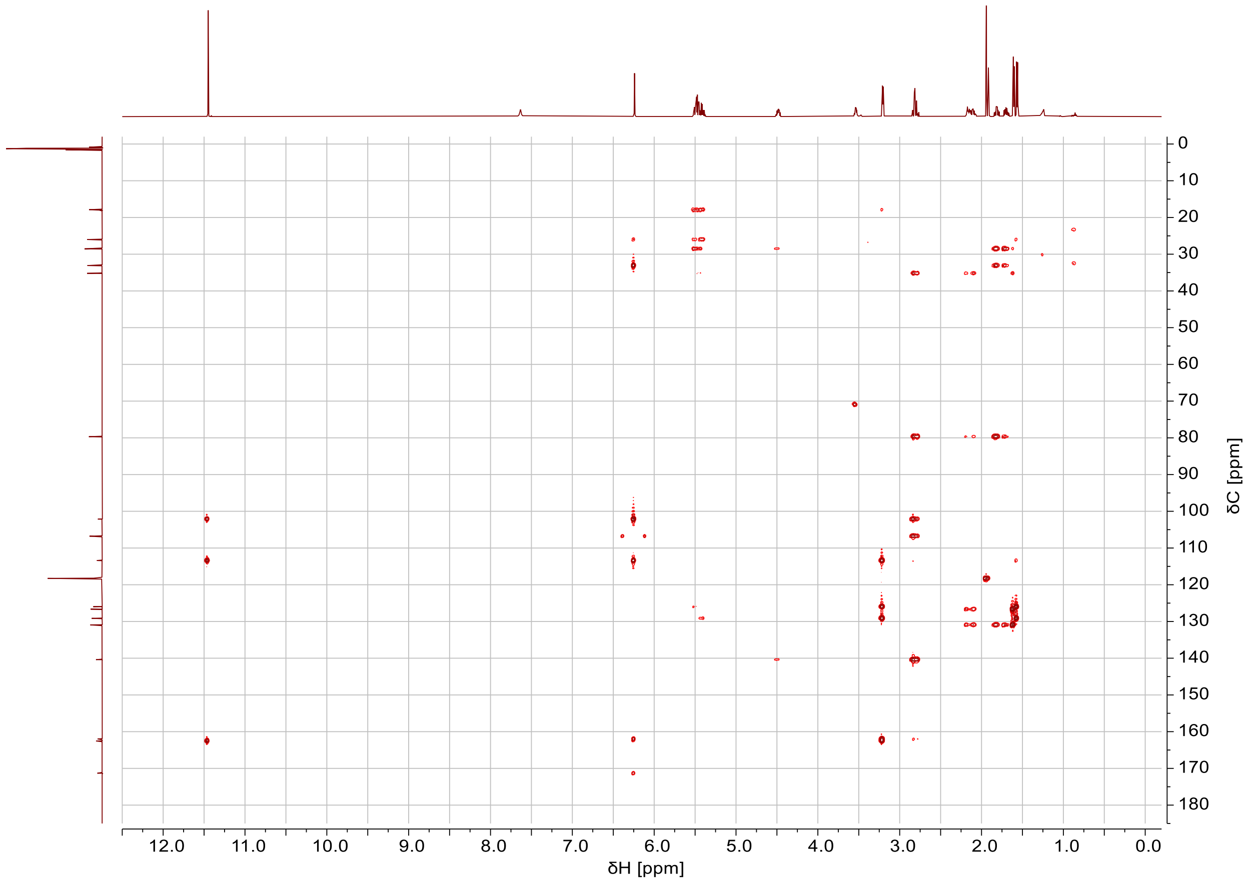

Figure S12 HMBC spectrum (MeCN-d_3) of compound **6**.

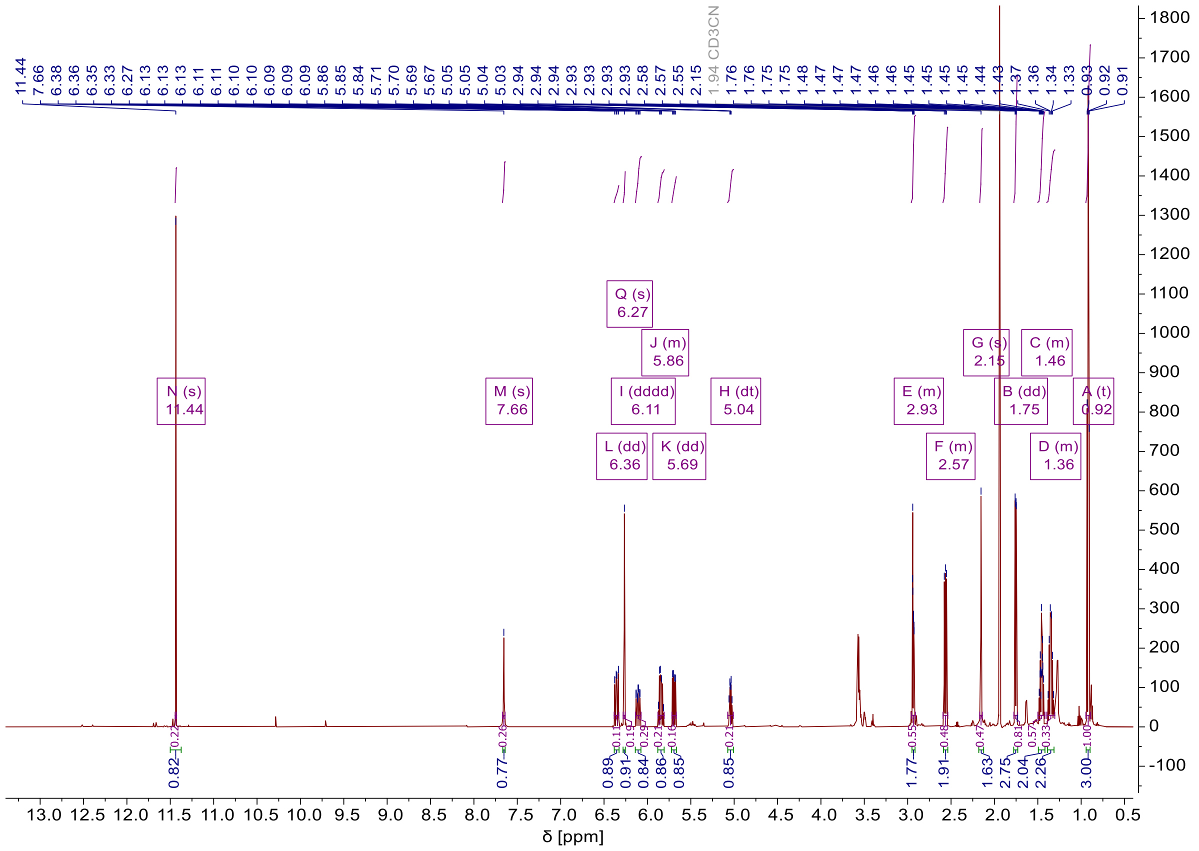

Figure S13 ^{1}H NMR spectrum (600 MHz, MeCN-d_3) of compound **9**.

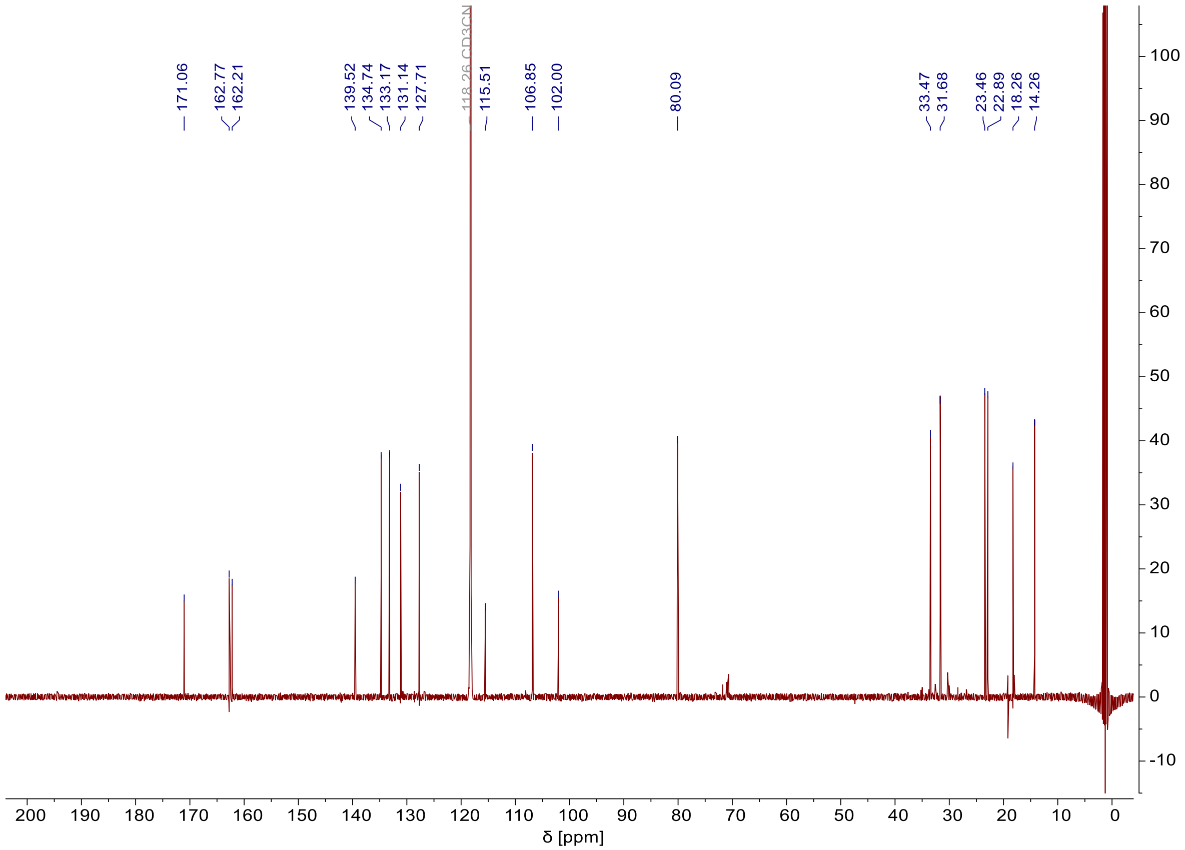

Figure S14 ^{13}C NMR spectrum (150 MHz, MeCN-d_3) of compound **9**.

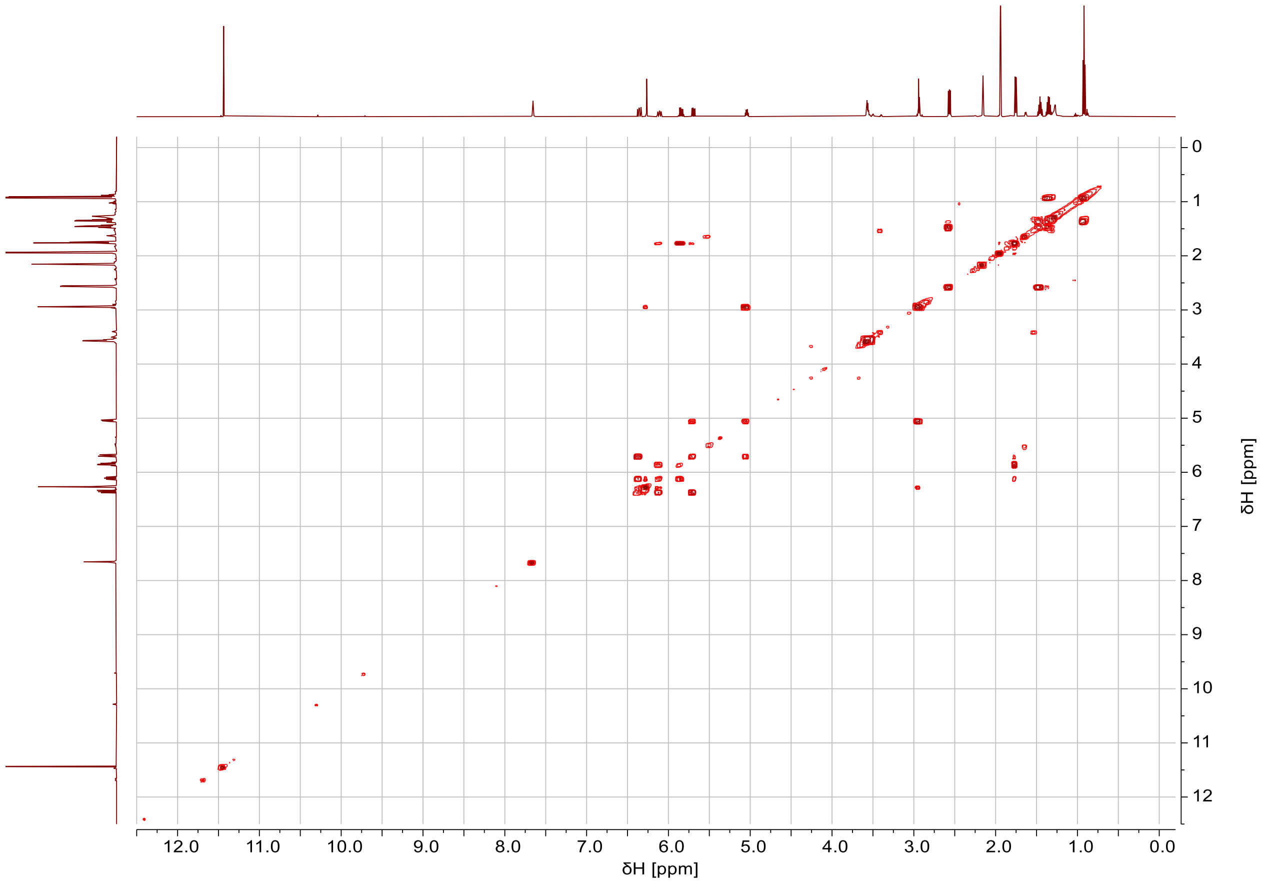

Figure S15 ^{1}H, ^{1}H COSY spectrum (MeCN-d_3) of compound **9**.

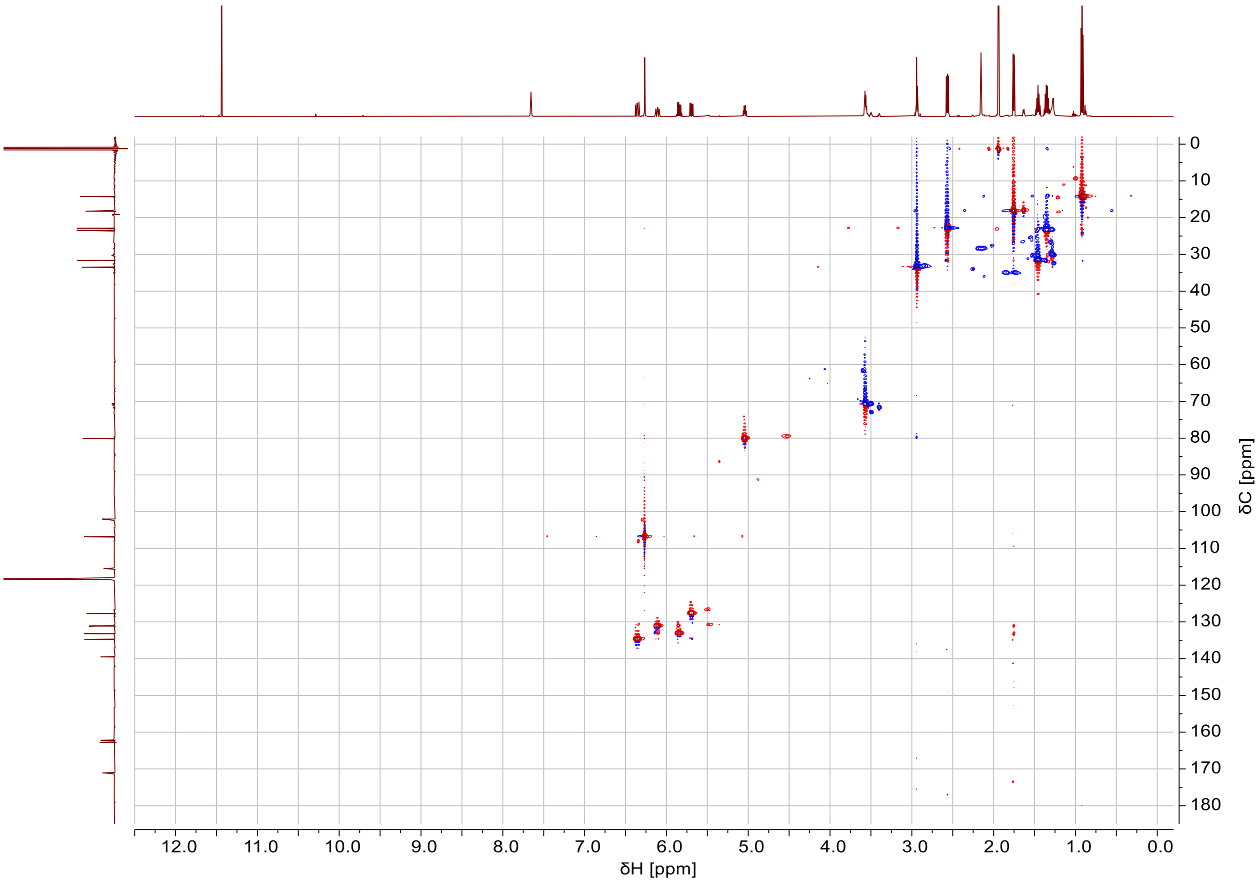

Figure S16 HSQC spectrum (MeCN-d_3) of compound **9**.

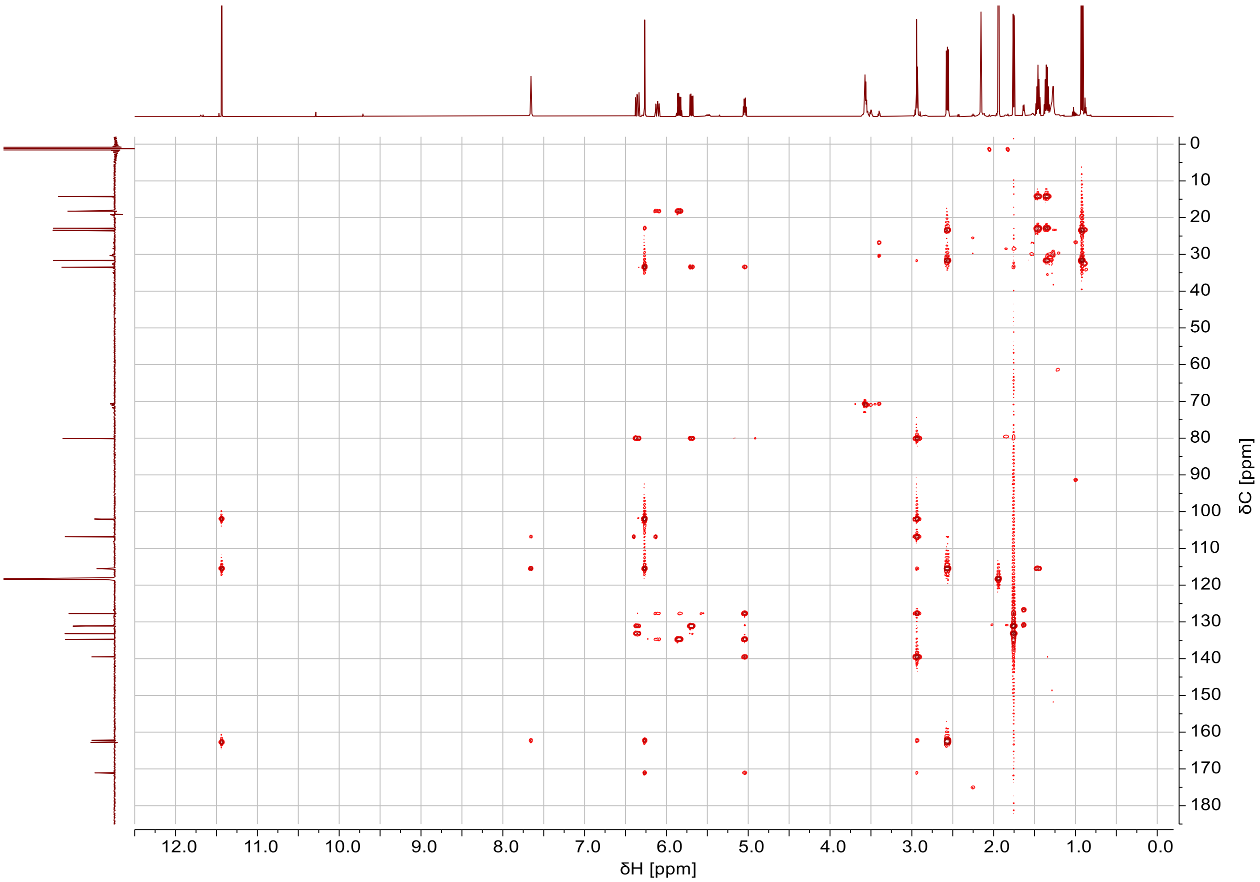

Figure S17 HMBC spectrum (MeCN-d_3) of compound **9**.

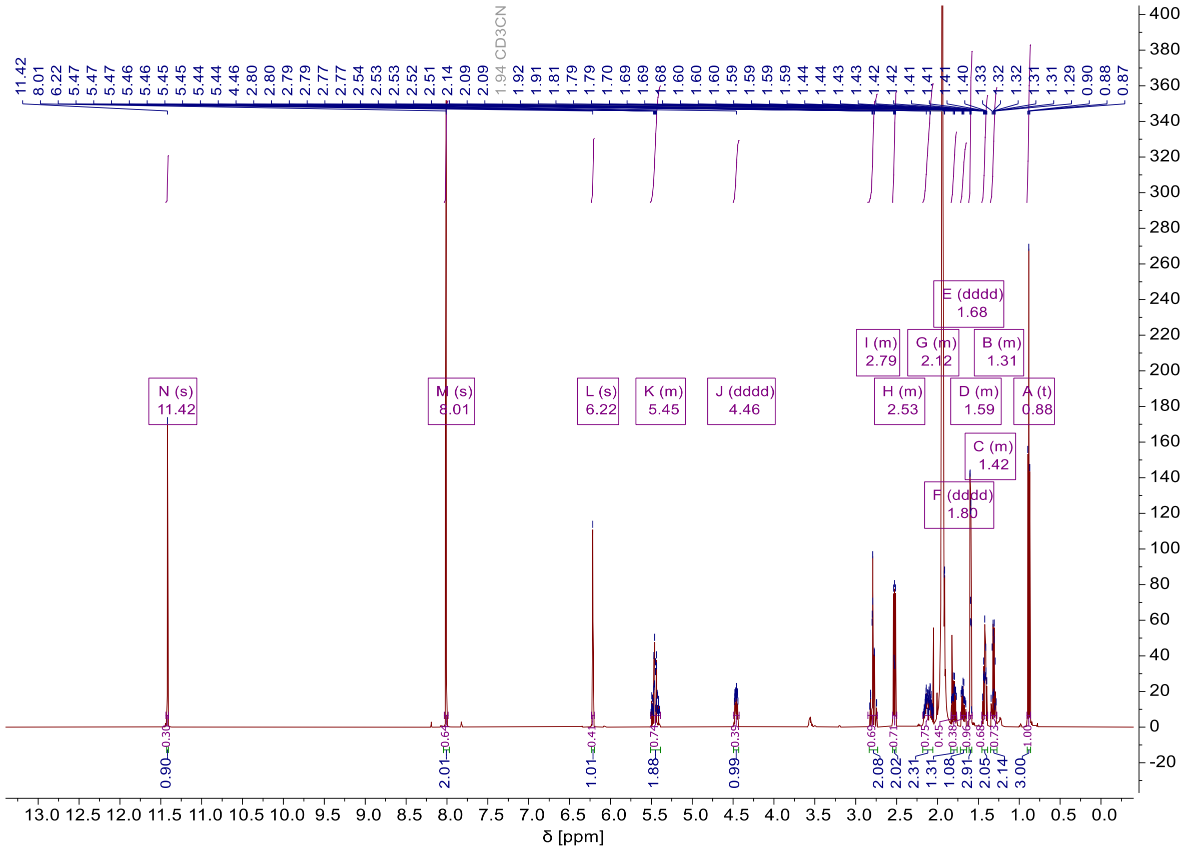

Figure S18 ^{1}H NMR spectrum (600 MHz, MeCN-d_3) of compound **7**.

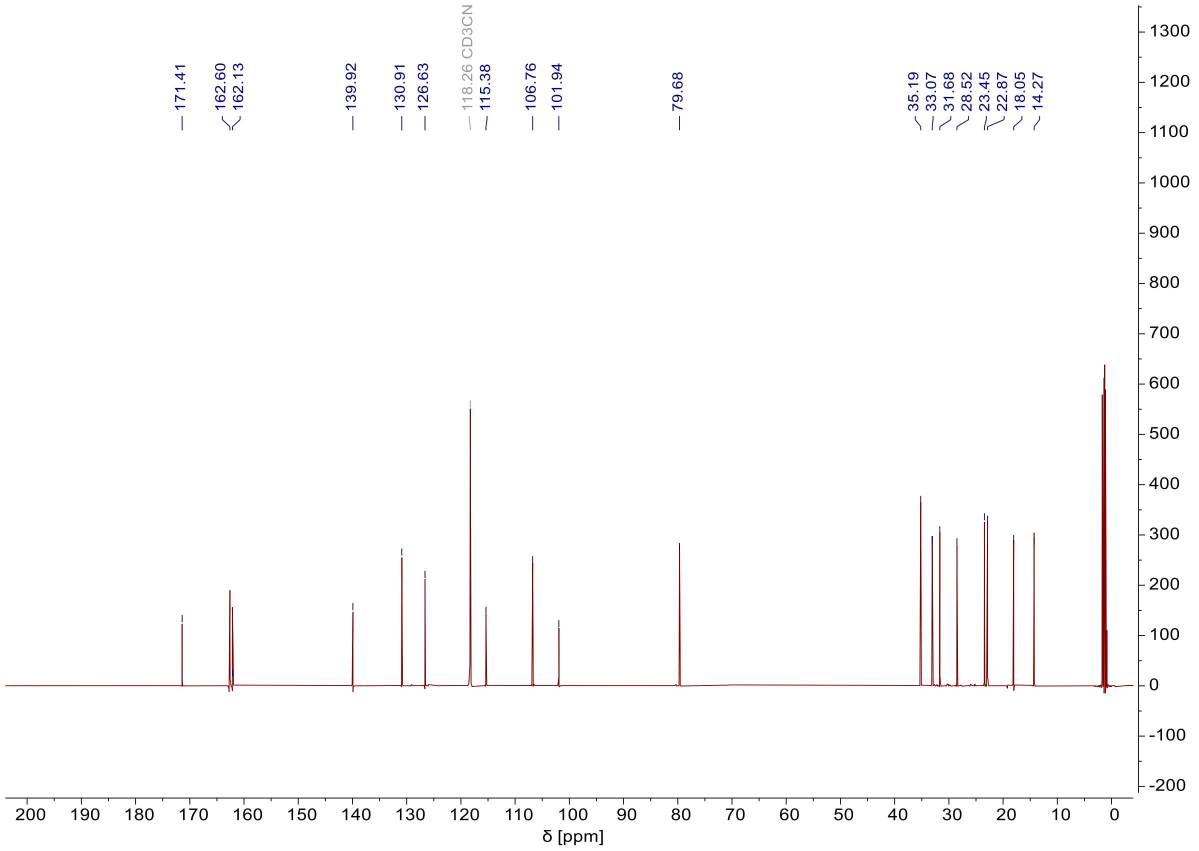

Figure S19 ^{13}C NMR spectrum (150 MHz, MeCN-d_3) of compound **7**.

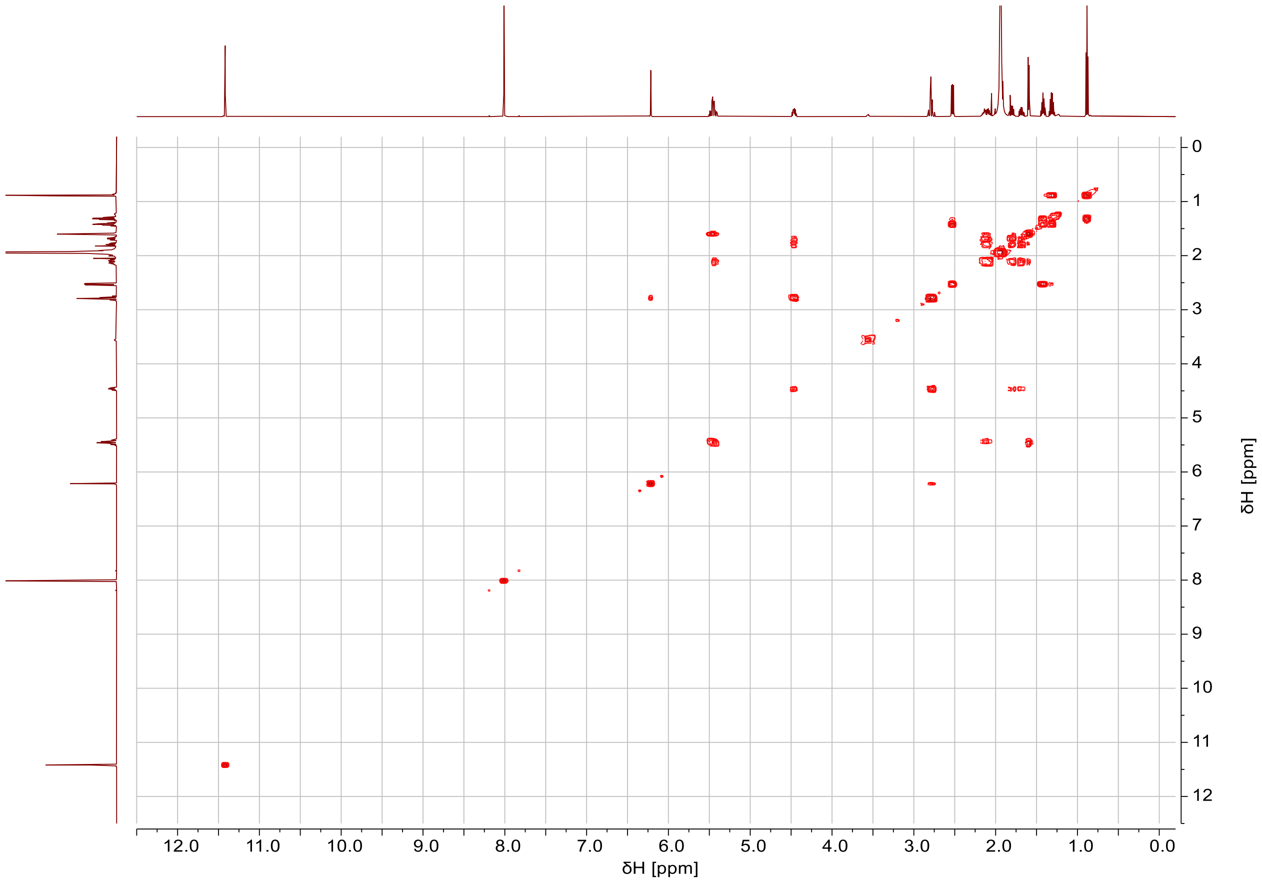

Figure S20 ^{1}H, ^{1}H COSY spectrum (MeCN-d_3) of compound **7**.

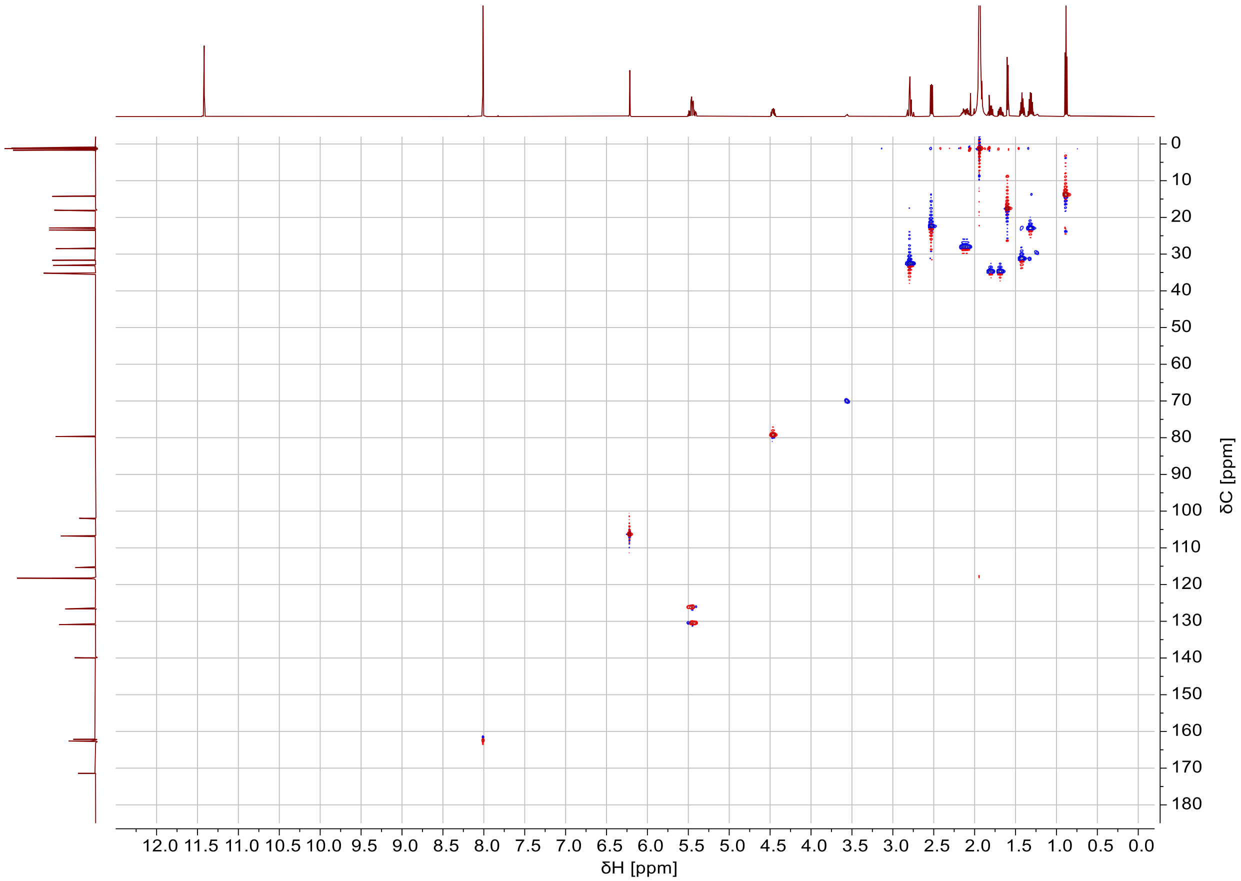

Figure S21 HSQC spectrum (MeCN-d_3) of compound **7**.

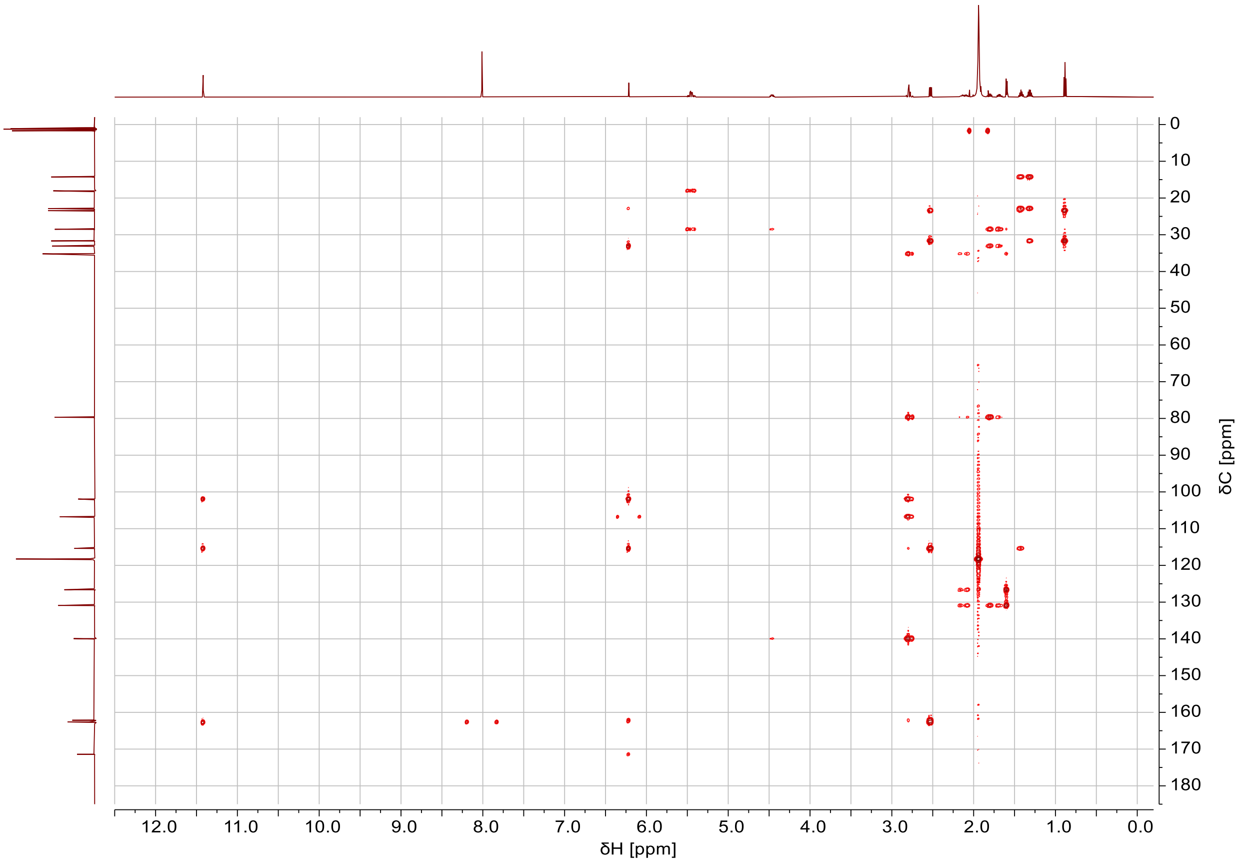

Figure S22 HMBC spectrum (MeCN-d_3) of compound **7**.

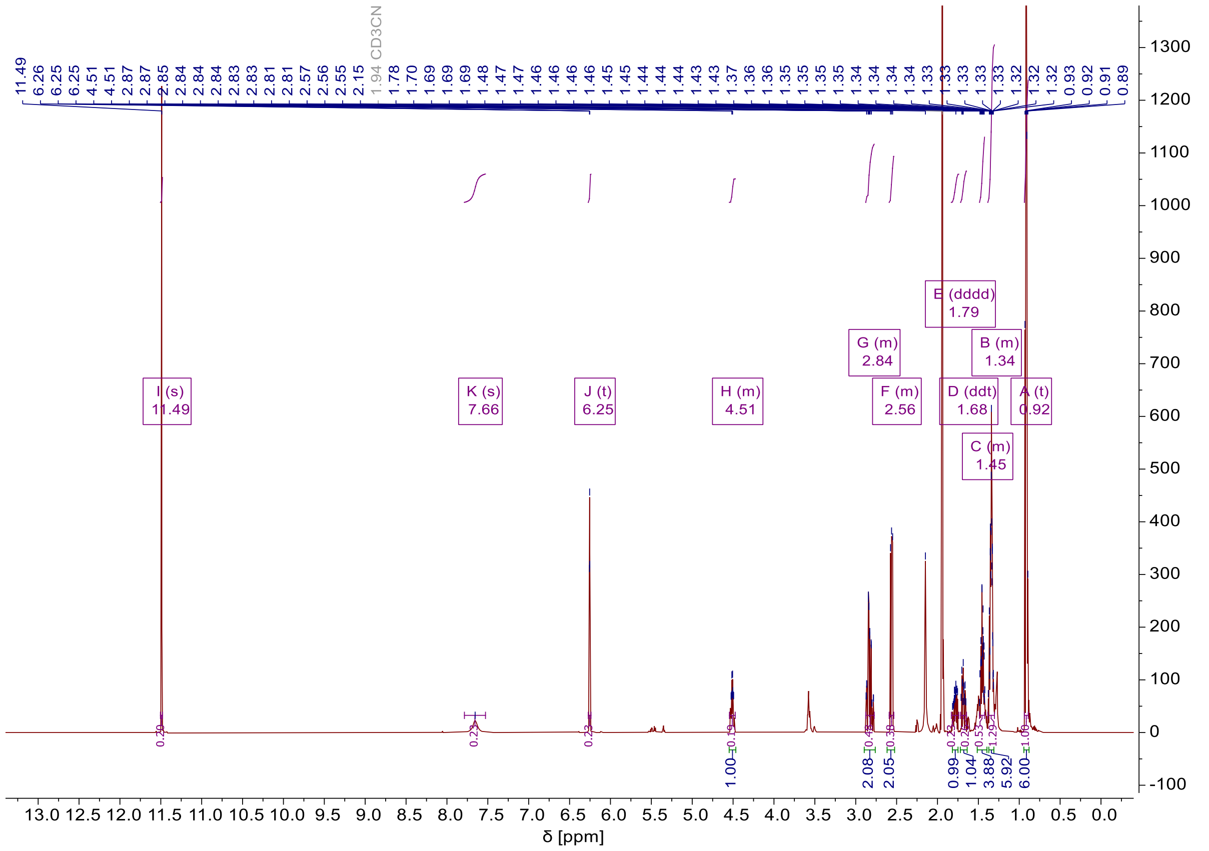

Figure S23: ^{1}H NMR spectrum (600 MHz, MeCN-d_3) of compound **8**.

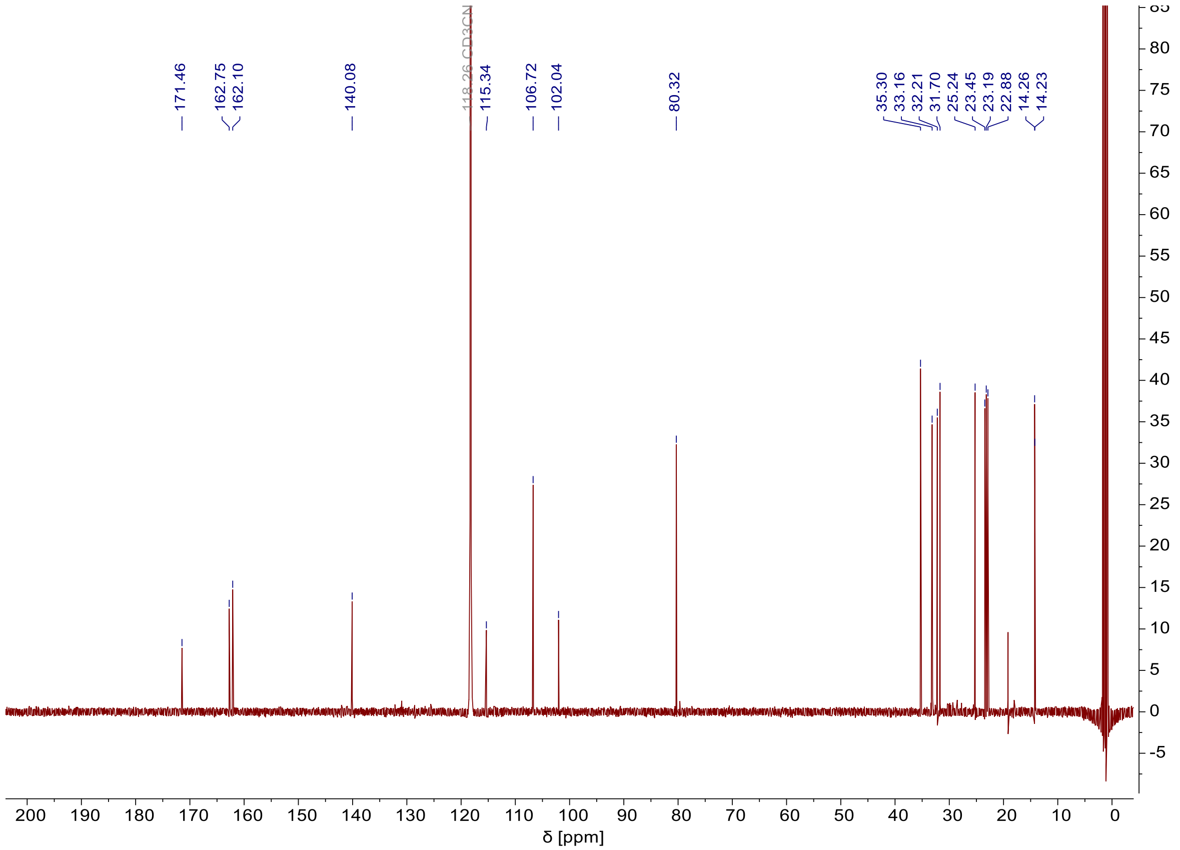

Figure S24 ^{13}C NMR spectrum (150 MHz, MeCN-d_3) of compound **8**.

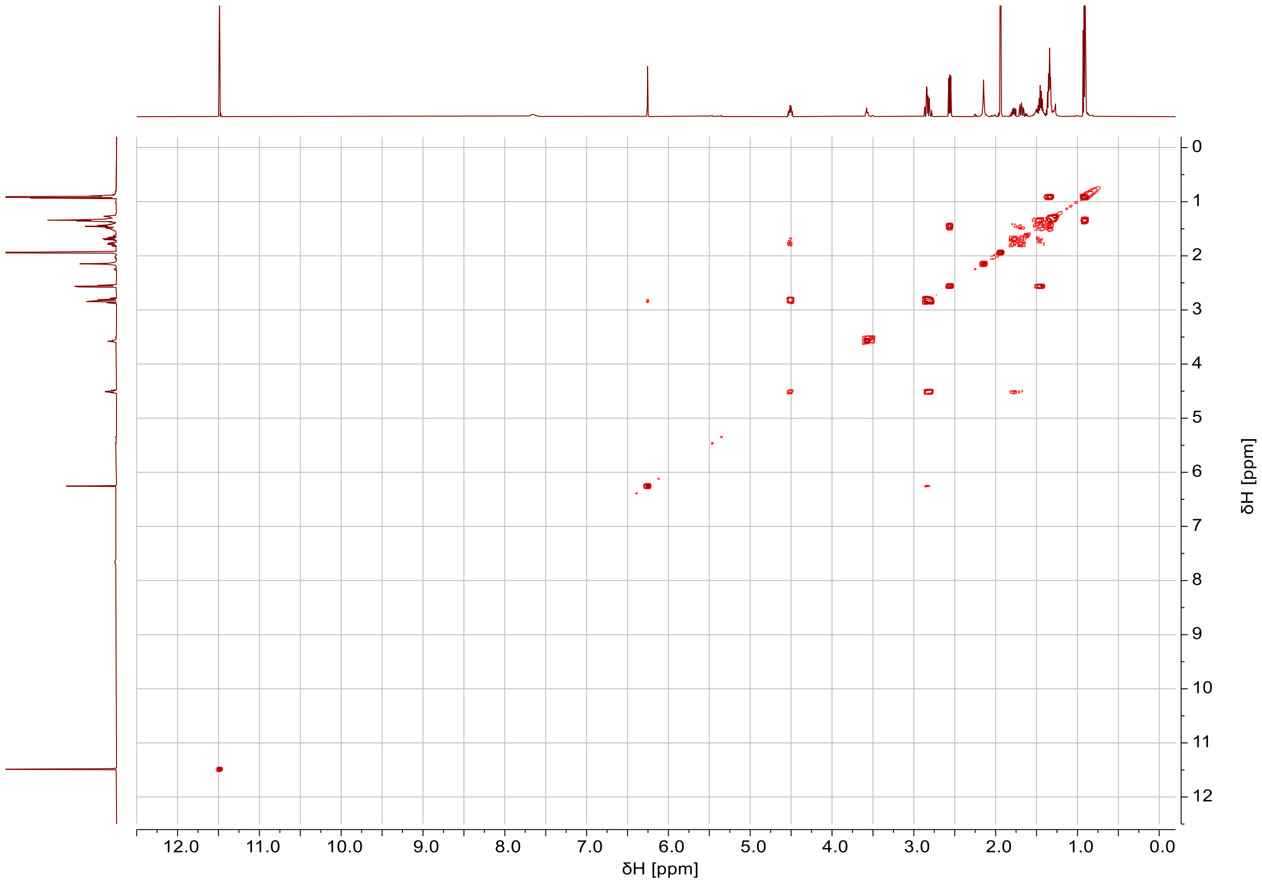

Figure S25 ^{1}H, ^{1}H COSY spectrum (MeCN-d_3) of compound **8**.

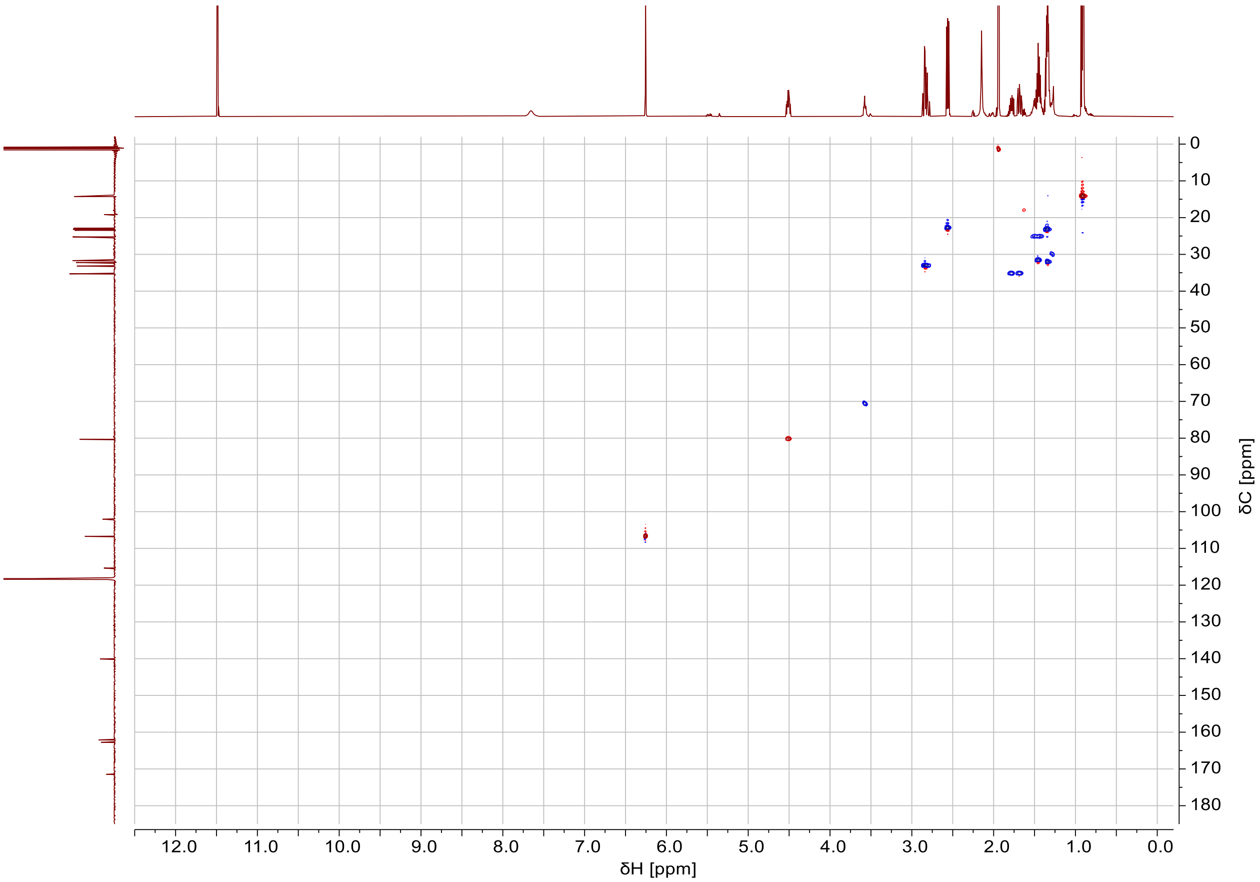

Figure S26 HSQC spectrum (MeCN-d_3) of compound **8**.

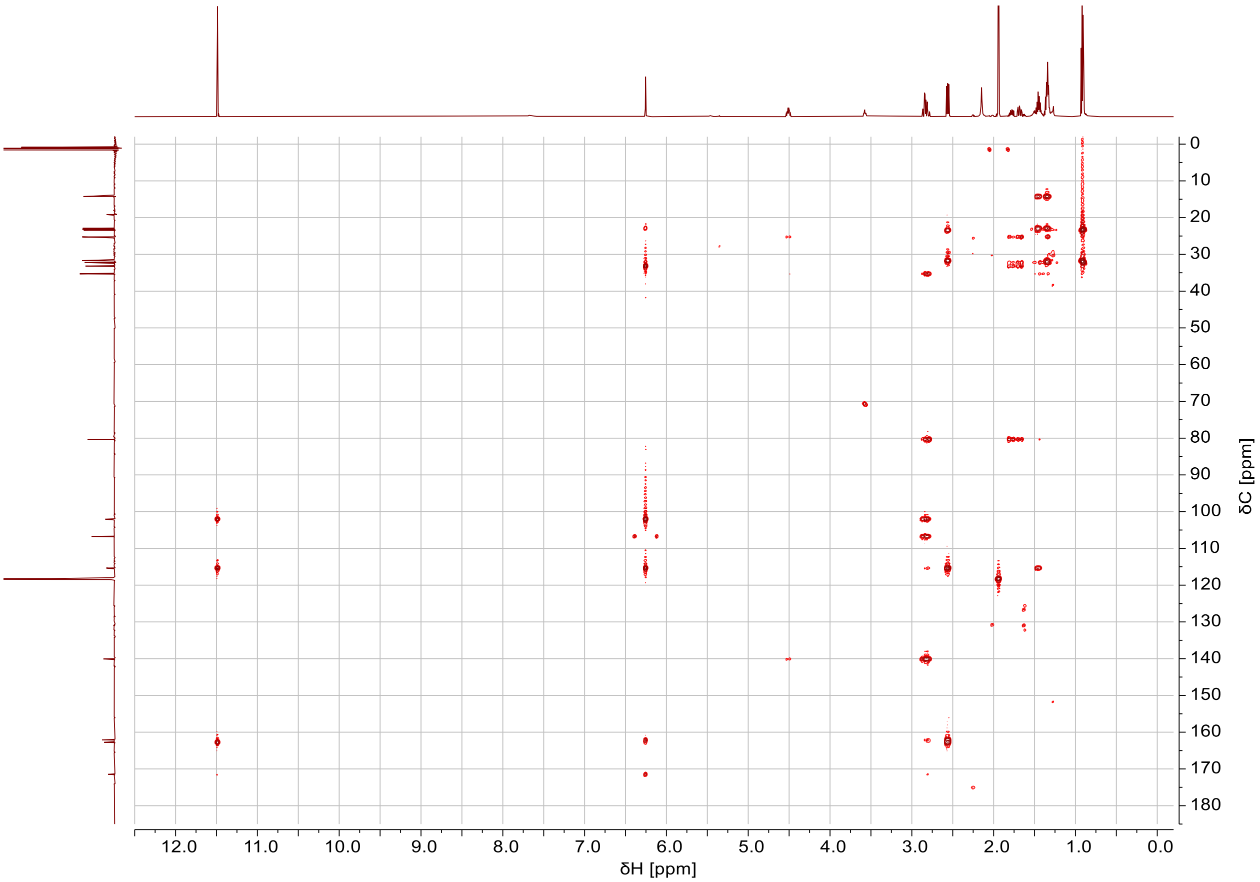

Figure S27 HMBC spectrum (MeCN-d_3) of compound **8**.

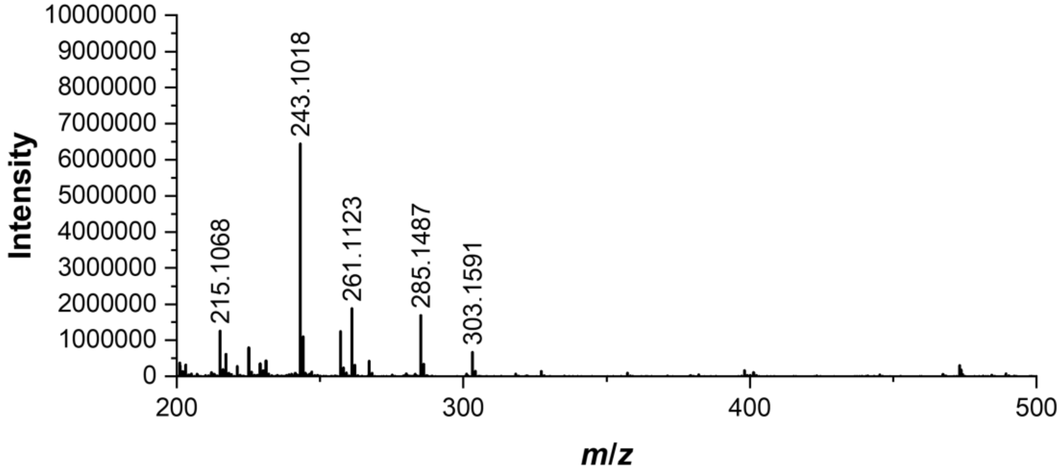

Figure S28 HRMS spectrum of compound **6**.

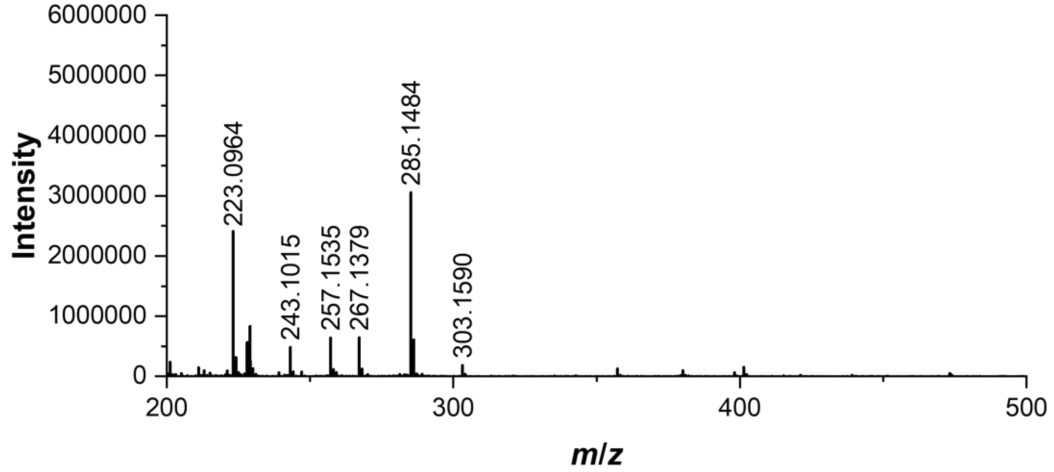

Figure S29 HRMS spectrum of compound **9**.

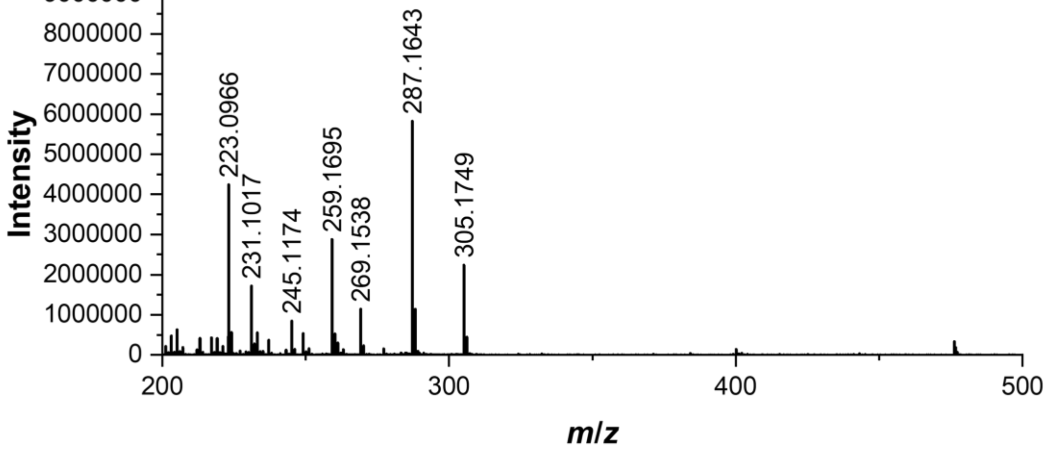

Figure S30 HRMS spectrum on compound **7**.

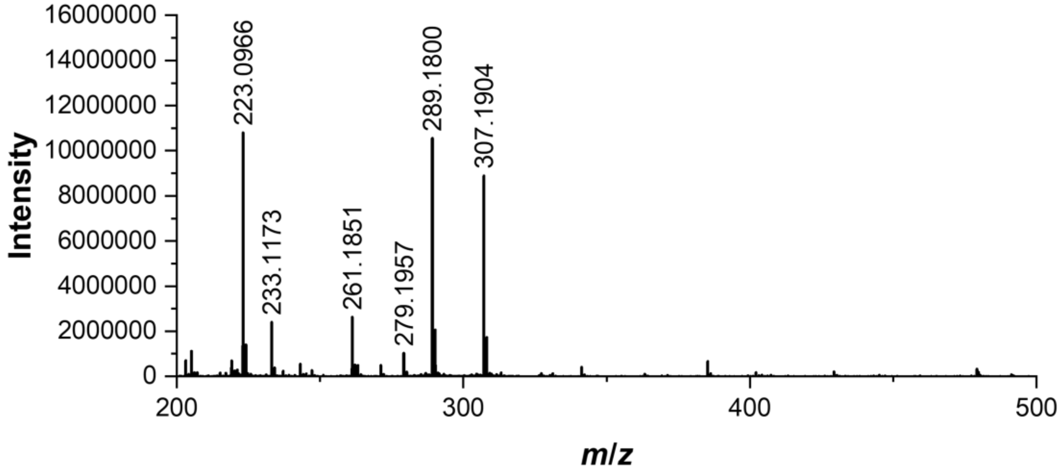

Figure S31 HRMS spectrum of compound **8**.

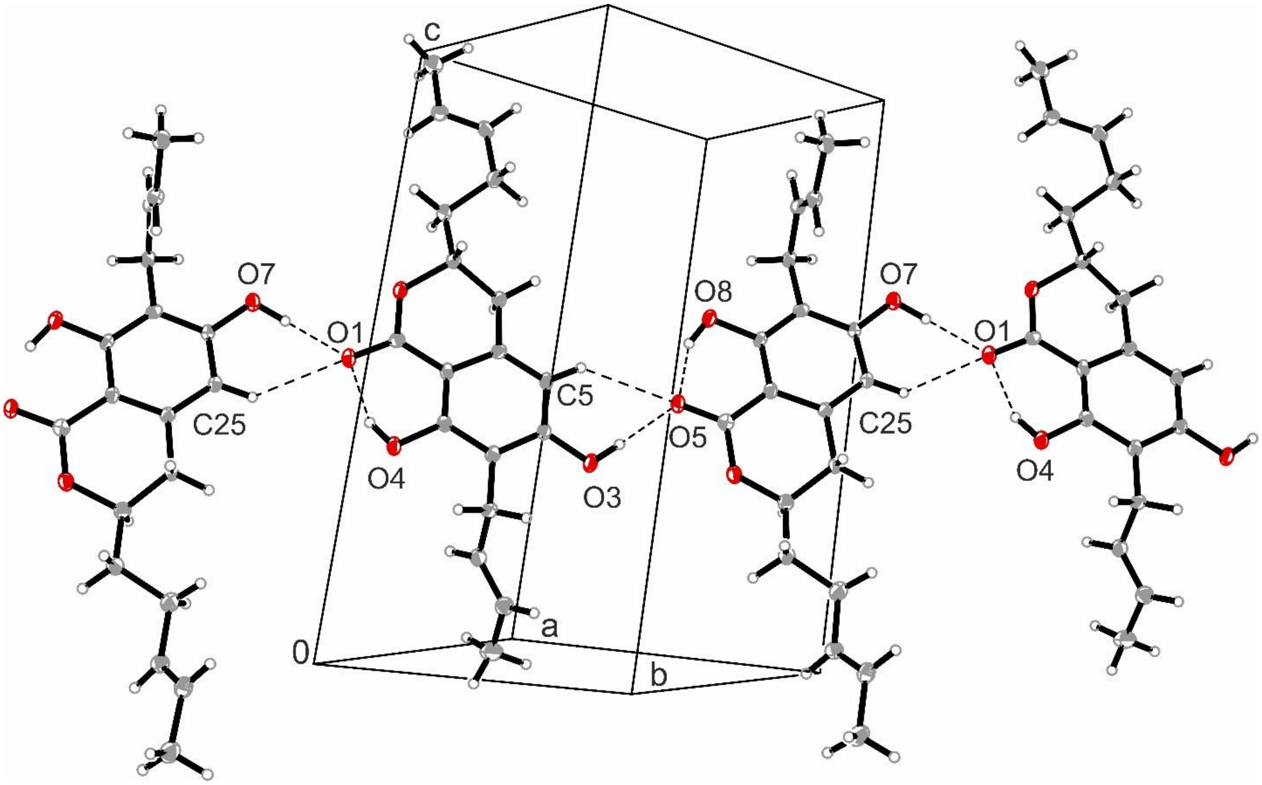

Figure S32 Excerpt of the packing diagram. The packaging diagram represents the formation of linear chains perpendicular to the ab-diagonal involving O-H…O and C-H…O interactions in compound **6**.

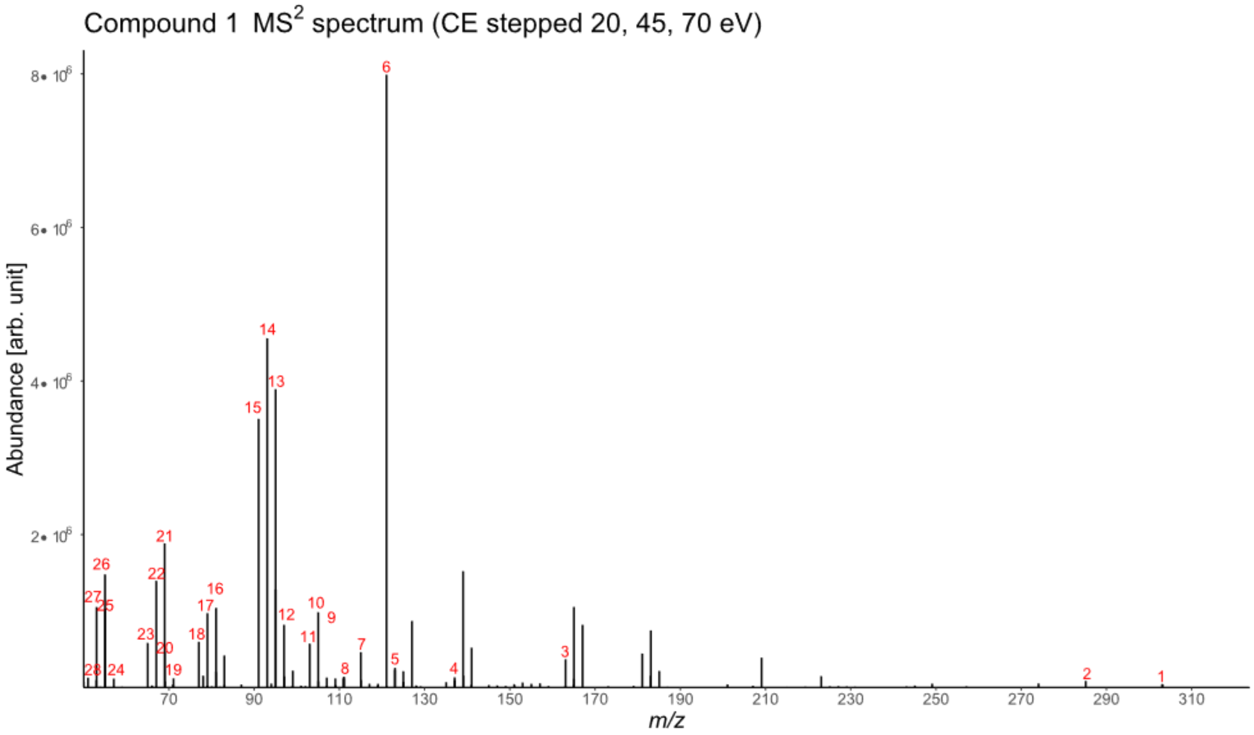

Figure S33 Full MS/MS spectrum of compound 1**.** The peak assignment is as follows: Peak 1: *m/z* = 303.1594, formula = $C_{18}H_{22}O_4$; Peak 2: *m/z* = 285.1485, formula = $C_{18}H_{20}O_3$; Peak 3: *m/z* =163.0754, formula =$C_{10}H_{10}O_2$; Peak 4: *m/z* = 137.0598, formula = $C_8H_8O_2$; Peak 5: *m/z* = 123.0443, formula = $C_7H_6O_2$; Peak 6: *m/z* = 121.0650, formula = C_8H_8O; Peak 7: *m/z* = 115.0545, formula = C_9H_6; Peak 8: *m/z* = 111.0444, formula = $C_6H_6O_2$; Peak 9: *m/z* = 107.0495, formula = C_7H_6O; Peak 10: *m/z* = 105.0703, formula = C_8H_8; Peak 11: *m/z* = 103.0546, formula = C_8H_6; Peak 12: *m/z* = 95.086, formula = C_7H_{10}; Peak 13: *m/z* = 95.0496, formula = C_6H_6O; Peak 14: *m/z* = 93.0704, formula = C_7H_8; Peak 15: *m/z* = 91.0548, formula = C_7H_6; Peak 16: *m/z* = 81.0705, formula = C_6H_8; Peak 17: *m/z* = 79.0549, formula = C_6H_6; Peak 18: *m/z* = 77.0392, formula = C_6H_4; Peak 19: *m/z* = 69.0706, formula = C_5H_8; Peak 20: *m/z* = 69.0342, formula = C_4H_4O; Peak 21: *m/z* = 68.9978, formula = C_3O_2; Peak 22: *m/z* = 67.0549, formula = C_5H_6; Peak 23: *m/z* = 65.0393, formula = C_5H_4; Peak 24: *m/z* = 57.0706, formula = C_4H_8; Peak 25: *m/z* = 55.055, formula = C_4H_6; Peak 26: *m/z* = 55.0186, formula = C_3H_2O; Peak 27: *m/z* = 53.0393, formula = C_4H_4; Peak 28: *m/z* = 53.003, formula = C_3O.

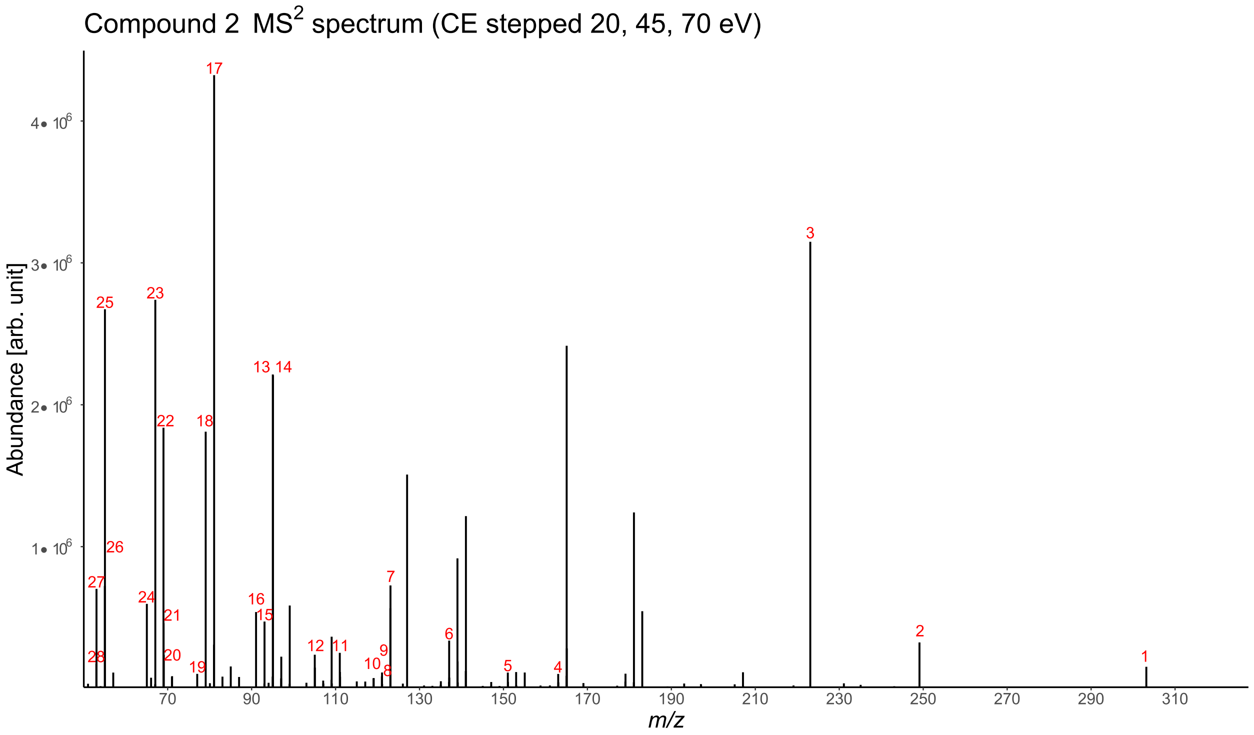

Figure S34 Full MS/MS spectrum of compound 2**.** The peak assignment is as follows: Peak 1: *m/z* = 303.1591, formula = $C_{18}H_{22}O_4$; Peak 2: *m/z* = 249.1121, formula = $C_{14}H_{16}O_4$; Peak 3: *m/z* = 223.0965, formula = $C_{12}H_{14}O_4$; Peak 4: *m/z* = 163.0389, formula = $C_9H_6O_3$; Peak 5: *m/z* = 151.0390, formula = $C_8H_6O_3$; Peak 6: *m/z* = 137.0598, formula = $C_8H_8O_2$; Peak 7: *m/z* = 123.0443, formula = $C_7H_6O_2$; Peak 8: *m/z* = 121.0650, formula = C_8H_8O; Peak 9: *m/z* = 121.0286, formula = $C_7H_4O_2$; Peak 10: *m/z* = 119.0858, formula = C_9H_{10}; Peak 11: *m/z* = 111,0444, formula = $C_6H_6O_2$; Peak 12: *m/z* = 105.0703, formula = C_8H_8; Peak 13: *m/z* = 95.0860, formula = C_7H_{10}; Peak 14: *m/z* = 95.0496, formula = C_6H_6O; Peak 15: *m/z* = 93.0704, formula = C_7H_8; Peak 16: *m/z* = 91.0548, formula = C_7H_6; Peak 17: *m/z* = 81.0705, formula = C_6H_8; Peak 18: *m/z* = 79.0549, formula = C_6H_6; Peak 19: *m/z* = 77,0392, formula = C_6H_4; Peak 20: *m/z* = 69.0706, formula = C_5H_8; Peak 21: *m/z* = 69.0342, formula = C_4H_4O; Peak 22: *m/z* = 68.9978, formula = C_3O_2; Peak 23: *m/z* = 67.0549, formula = C_5H_6; Peak 24: *m/z* = 65.0393, formula = C_5H_4; Peak 25: *m/z* = 55.0550, formula = C_4H_6; Peak 26: *m/z* = 55.0186, formula = C_3H_2O; Peak 27: *m/z* = 53.0393, formula = C_4H_4; Peak 28: *m/z* = 53.0030, formula = C_3O.

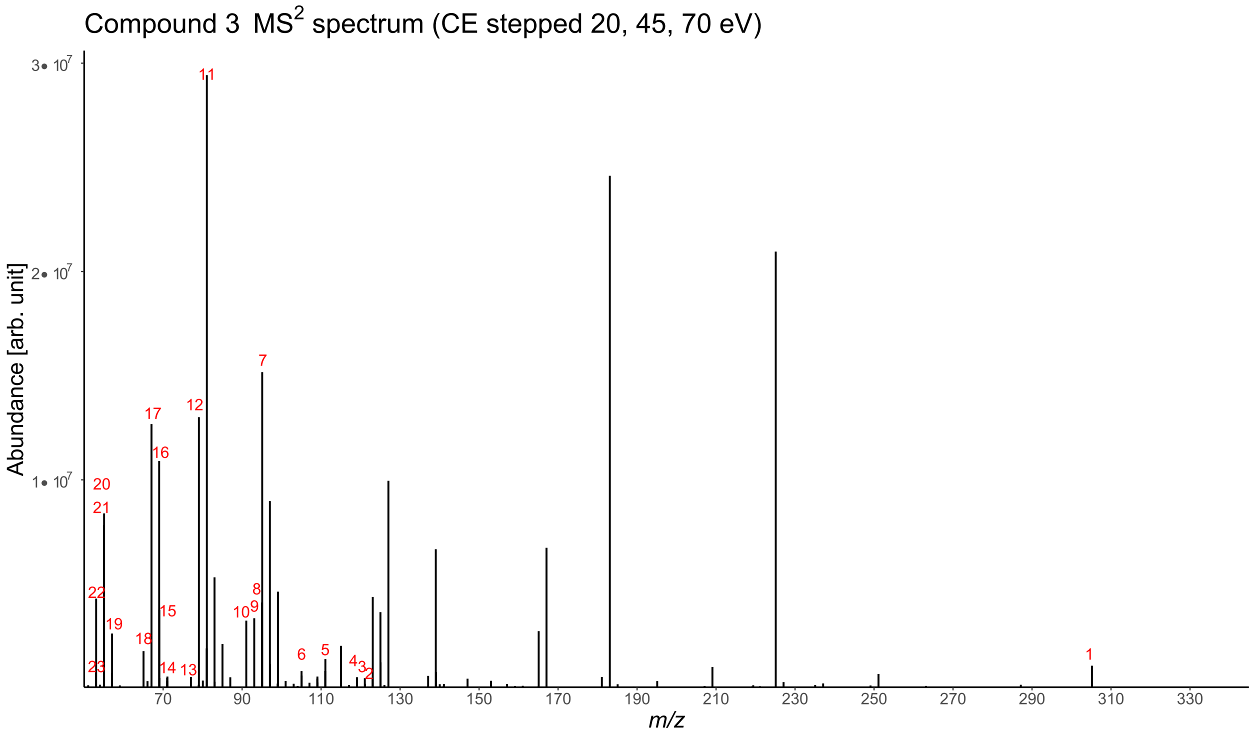

Figure S35 Full MS/MS spectrum of compound 3**.** The peak assignment is as follows: Peak 1: *m/z* = 305.1747, formula = $C_{18}H_{24}O_4$; Peak 2: *m/z* = 123.0443, formula = $C_7H_6O_2$; Peak 3: *m/z* = 121.0651, formula = C_8H_8O; Peak 4: *m/z* = 119.0858, formula = C_9H_{10}; Peak 5: *m/z* = 111.0445, formula = $C_6H_6O_2$; Peak 6: *m/z* = 105.0704, formula = C_8H_8; Peak 7: *m/z* = 95.0861, formula = C_7H_{10}; Peak 8 : *m/z* = 95.0497, formula = C_6H_6O; Peak 9: *m/z* = 93.0704, formula = C_7H_8; Peak 10: *m/z* = 91.0548, formula = C_7H_6; Peak 11: *m/z* = 81.0705, formula = C_6H_8; Peak 12: *m/z* = 79.0549, formula = C_6H_6; Peak 13: *m/z* = 77.0393, formula = C_6H_4; Peak 14: *m/z* = 69.0706, formula = C_5H_8; Peak 15: *m/z* = 69.0342, formula = C_4H_4O; Peak 16: *m/z* = 68.9978, formula = C_3O_2; Peak 17: *m/z* = 67.0550, formula = C_5H_6; Peak 18: *m/z* = 65.0393, formula = C_5H_4; Peak 19: *m/z* = 57.0707, formula = C_4H_8; Peak 20: *m/z* = 55.0550, formula = C_4H_6; Peak 21: *m/z* = 55.0186, formula = C_3H_2O; Peak 22: *m/z* = 53.0394, formula = C_4H_4; Peak 23: *m/z* = 53.0030, formula = C_3O.

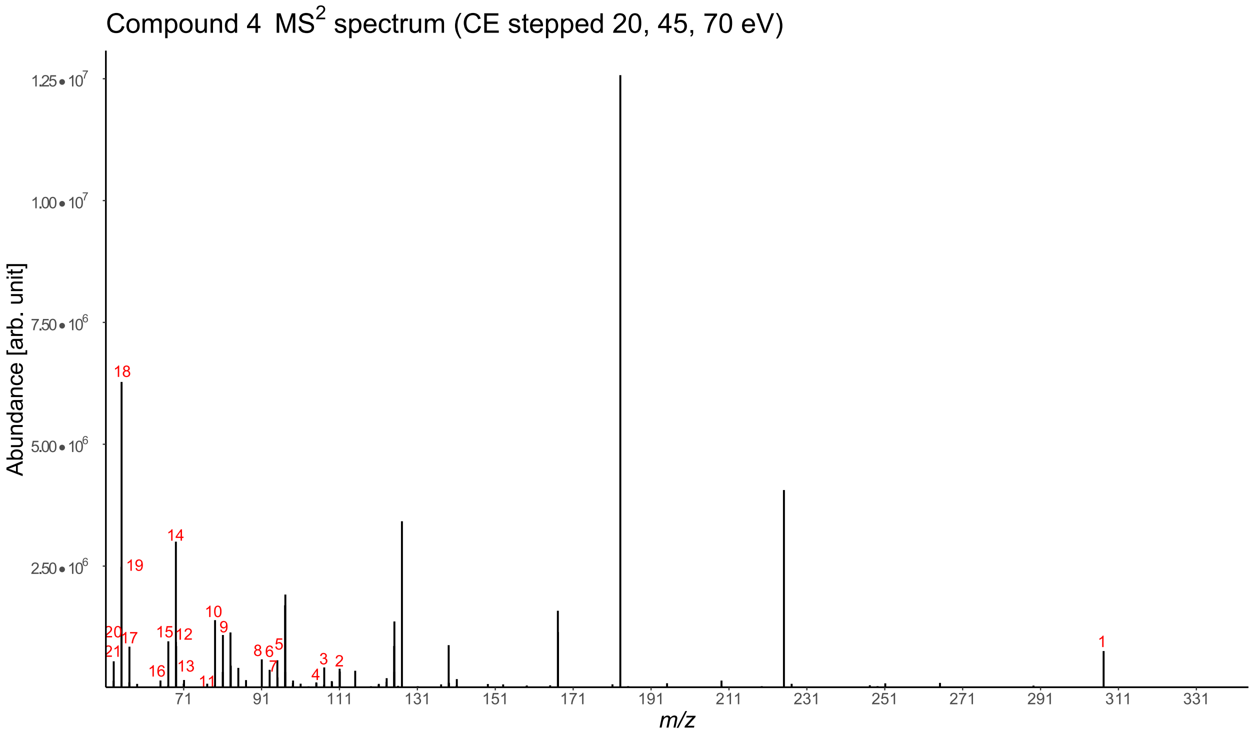

Figure S36 Full MS/MS spectrum of compound 4**.** The peak assignment is as follows: Peak 1: *m/z* = 307.1903, formula = $C_{18}H_{26}O_4$; Peak 2: *m/z* = 111.0444, formula = $C_6H_6O_2$; Peak 3: *m/z* = 107.0859, formula = C_8H_{10}; Peak 4: *m/z* = 105.0703, formula = C_8H_8; Peak 5: *m/z* = 95.0860, formula = C_7H_{10}; Peak 6: *m/z* = 95.0496, formula = C_6H_6O; Peak 7: *m/z* = 93.0704, formula = C_7H_8; Peak 8: *m/z* = 91.0548, formula = C_7H_6; Peak 9: *m/z* = 81.0705, formula = C_6H_8; Peak 10: *m/z* = 79.0549, formula = C_6H_6; Peak 11: *m/z* = 77.0392, formula = C_6H_4; Peak 12: *m/z* = 69.0706, formula = C_5H_8; Peak 13: *m/z* = 69.0342, formula = C_4H_4O; Peak 14: *m/z* = 68.9978, formula = C_3O_2; Peak 15: *m/z* = 67.0549, formula = C_5H_6; Peak 16: *m/z* = 65.0393, formula = C_5H_4; Peak 17: *m/z* = 57.0706, formula = C_4H_8; Peak 18: *m/z* = 55.0550, formula = C_4H_6; Peak 19: *m/z* = 55.0186, formula = C_3H_2O; Peak 20: *m/z* = 53.0393, formula = C_4H_4; Peak 21: *m/z* = 53.0030, formula = C_3O.

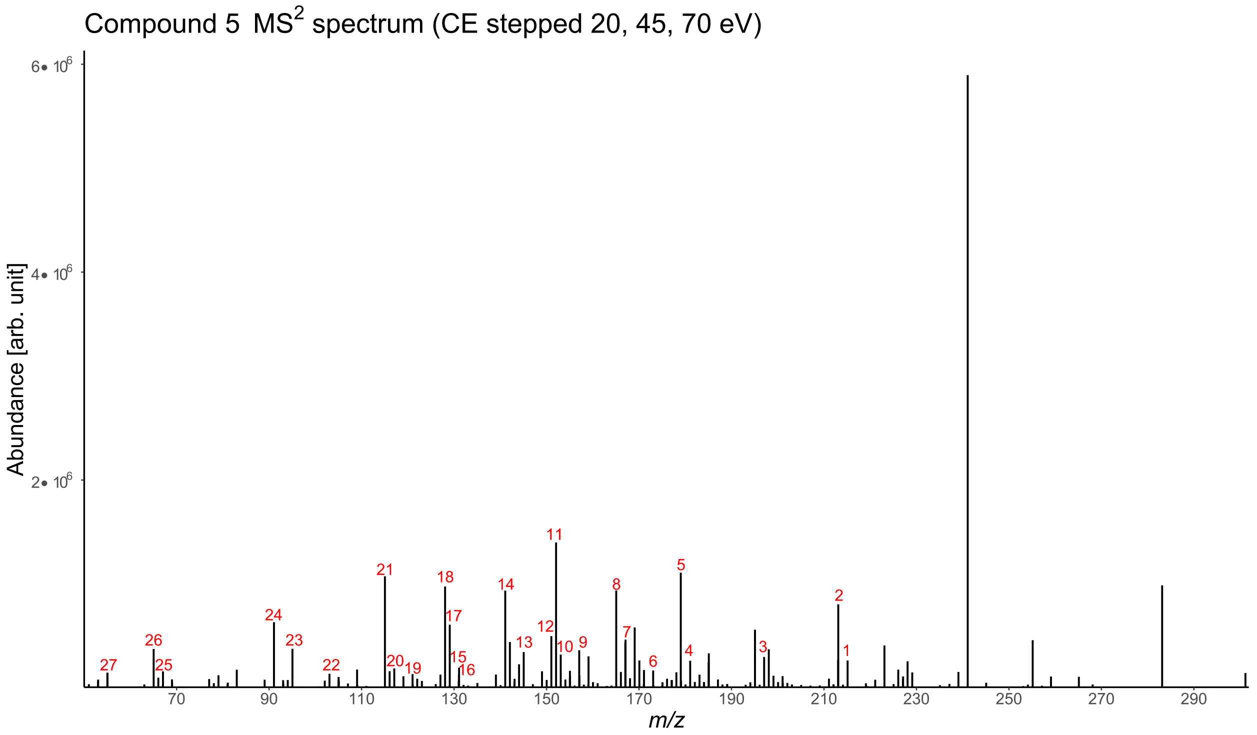

Figure S37 Full MS/MS spectrum of compound 5**.** The peak assignment is as follows: Peak 1: *m/z* = 215.1067, formula = $C_{14}H_{14}O_2$; Peak 2: *m/z* = 213.0910, formula = $C_{14}H_{12}O_2$; Peak 3: *m/z* = 197.0598, formula = $C_{13}H_8O_2$; Peak 4: *m/z* = 181.0648, formula = $C_{13}H_8O$; Peak 5: *m/z* = 179.0339, formula = $C_9H_6O_4$; Peak 6: *m/z* = 173.0598, formula = $C_{11}H_8O_2$; Peak 7: *m/z* = 167.0340, formula = $C_8H_6O_4$; Peak 8: *m/z* = 165.0700, formula = $C_{13}H_8$; Peak 9: *m/z* = 157.0649, formula = $C_{11}H_8O$; Peak 10: *m/z* = 153.0699, formula = $C_{12}H_8$; Peak 11: *m/z* = 152.0621, formula = $C_{12}H_7$; Peak 12: *m/z* = 151.0390, formula = $C_8H_6O_3$; Peak 13: *m/z* = 145.0648, formula = $C_{10}H_8O$; Peak 14: *m/z* = 141.0699, formula = $C_{11}H_8$; Peak 15: *m/z* = 131.0857, formula = $C_{10}H_{10}$; Peak 16: *m/z* = 131.049, formula =C_9H_6O; Peak 17: *m/z* = 129.0700, formula = $C_{10}H_8$; Peak 18: *m/z* = 128.0622, formula = $C_{10}H_7$; Peak 19: *m/z* = 121.0286, formula = $C_7H_4O_2$; Peak 20: *m/z* = 117.0701, formula = C_9H_8; Peak 21: *m/z* = 115.0545, formula = C_9H_6; Peak 22: *m/z* = 103.0546, formula = C_8H_6; Peak 23: *m/z* = 95.0496, formula = C_6H_6O; Peak 24: *m/z* = 91.0548, formula = C_7H_6; Peak 25: *m/z* = 67.0549, formula = C_5H_6; Peak 26: *m/z* = 65.0393, formula = C_5H_4; Peak 27: *m/z* = 55.0186, formula = C_3H_2O.

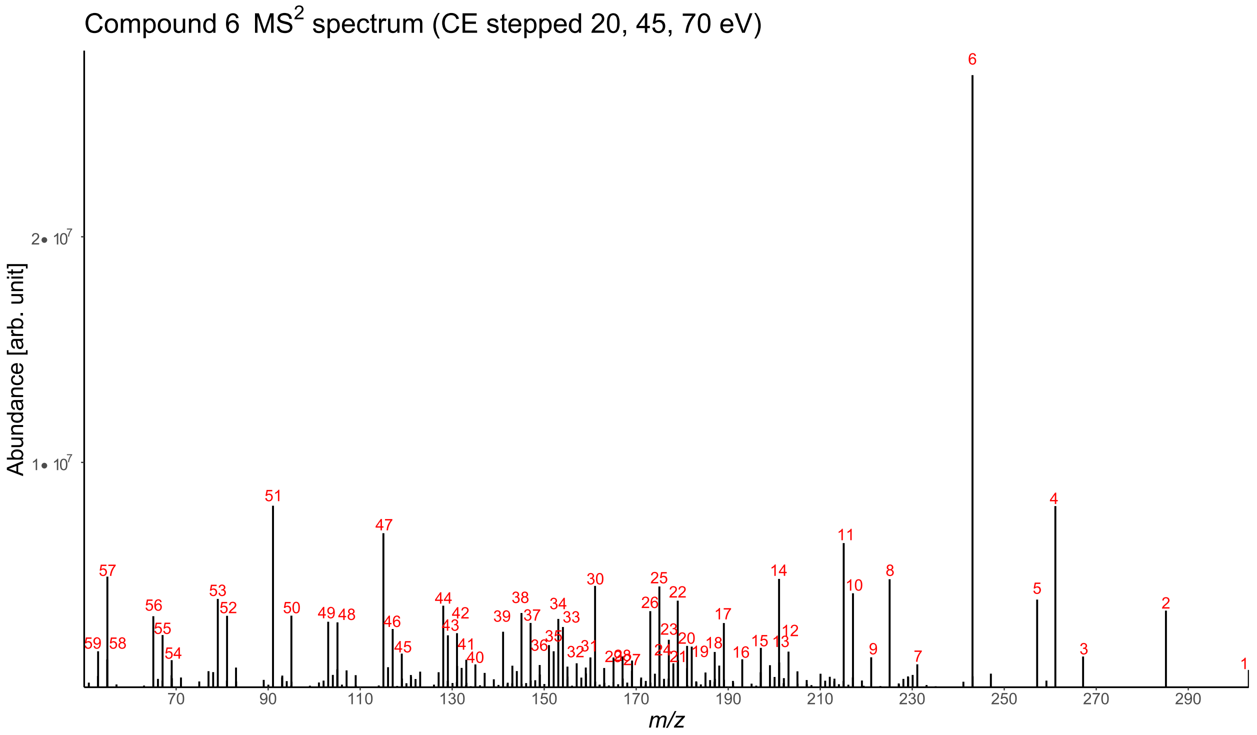

Figure S38 Full MS/MS spectrum of compound 6. The peak assignment is as follows: Peak 1: *m/z* = 303.1590, formula = $C_{18}H_{22}O_4$; Peak 2 : *m/z* = 285.1483, formula = $C_{18}H_{20}O_3$; Peak 3: *m/z* = 267.1378, formula = $C_{18}H_{18}O_2$; Peak 4: *m/z* = 261.1120, formula = $C_{15}H_{16}O_4$; Peak 5: *m/z* = 257.1534, formula = $C_{17}H_{20}O_2$; Peak 6: *m/z* = 243.1015, formula = $C_{15}H_{14}O_3$; Peak 7: *m/z* = 231.1015, formula = $C_{14}H_{14}O_3$; Peak 8: *m/z* = 225.0909, formula = $C_{15}H_{12}O_2$; Peak 9: *m/z* = 221.0807, formula = $C_{12}H_{12}O_4$; Peak 10: *m/z* = 217.1223, formula = $C_{14}H_{16}O_2$; Peak 11: *m/z* = 215.1066, formula = $C_{14}H_{14}O_2$; Peak 12: *m/z* = 203.1066, formula = $C_{13}H_{14}O_2$; Peak 13: *m/z* = 201.0909, formula = $C_{13}H_{12}O_2$; Peak 14: *m/z* = 201.0546, formula = $C_{12}H_8O_3$; Peak 15: *m/z* = 197.0961, formula = $C_{14}H_{12}O$; Peak 16: *m/z* = 193.0495, formula = $C_{10}H_8O_4$; Peak 17: *m/z* = 189.0546, formula = $C_{11}H_8O_3$; Peak 18: *m/z* = 187.0754, formula = $C_{12}H_{10}O_2$; Peak 19: *m/z* = 182.0726, formula = $C_{13}H_9O$; Peak 20: *m/z* = 181.0648, formula = $C_{13}H_8O$; Peak 21: *m/z* = 179.0855, formula = $C_{14}H_{10}$; Peak 22: *m/z* = 179.0338, formula = $C_9H_6O_4$; Peak 23: *m/z* = 177.0910, formula = $C_{11}H_{12}O_2$; Peak 24: *m/z* = 175.0754, formula = $C_{11}H_{10}O_2$; Peak 25: *m/z* = 175.0390, formula = $C_{10}H_6O_3$; Peak 26: *m/z* = 173.0597, formula = $C_{11}H_8O_2$; Peak 27: *m/z* = 169.1012, formula = $C_{13}H_{12}$; Peak 28: *m/z* = 167.0339, formula = $C_8H_6O_4$; Peak 29: *m/z* = 165.0699, formula = $C_{13}H_8$; Peak 30: *m/z* = 161.0597, formula = $C_{10}H_8O_2$; Peak 31: *m/z* = 160.0519, formula = $C_{10}H_7O_2$; Peak 32: *m/z* = 157.0648, formula = $C_{11}H_8O$; Peak 33: *m/z* = 154.0777, formula = $C_{12}H_9$; Peak 34: *m/z* = 153.0699, formula = $C_{12}H_8$; Peak 35: *m/z* = 152.0620, formula = $C_{12}H_7$; Peak 36: *m/z* = 151.0390, formula = $C_8H_6O_3$; Peak 37: *m/z* = 147.0440, formula = $C_9H_6O_2$; Peak 38: *m/z* = 145.0648, formula = $C_{10}H_8O$; Peak 39: *m/z* = 141.0699, formula = $C_{11}H_8$; Peak 40 : *m/z* = 135.0441, formula = $C_8H_6O_2$; Peak 41: *m/z* = 133.0649, formula = C_9H_8O; Peak 42: *m/z* = 131.0492, formula = C_9H_6O; Peak 43: *m/z* = 129.0700, formula = $C_{10}H_8$; Peak 44: *m/z* = 128.0622, formula = $C_{10}H_7$; Peak 45: *m/z* = 119.0494, formula = C_8H_6O; Peak 46: *m/z* = 117.0701, formula = C_9H_8; Peak 47: *m/z* = 115.0545, formula = C_9H_6; Peak 48: *m/z* = 105.0703, formula = C_8H_8; Peak 49: *m/z* = 103.0546, formula = C_8H_6; Peak 50: *m/z* = 95.0496, formula = C_6H_6O; Peak 51: *m/z* = 91.0547, formula = C_7H_6; Peak 52: *m/z* = 81.0705, formula = C_6H_8; Peak 53: *m/z* = 79.0548, formula = C_6H_6; Peak 54: *m/z* = 69.0342, formula = C_4H_4O; Peak 55: *m/z* = 67.0549, formula = C_5H_6; Peak 56: *m/z* = 65.0393, formula = C_5H_4; Peak 57: *m/z* = 55.0550, formula = C_4H_6; Peak 58: *m/z* = 55.0186, formula = C_3H_2O; Peak 59: *m/z* = 53.0393, formula = C_4H_4.

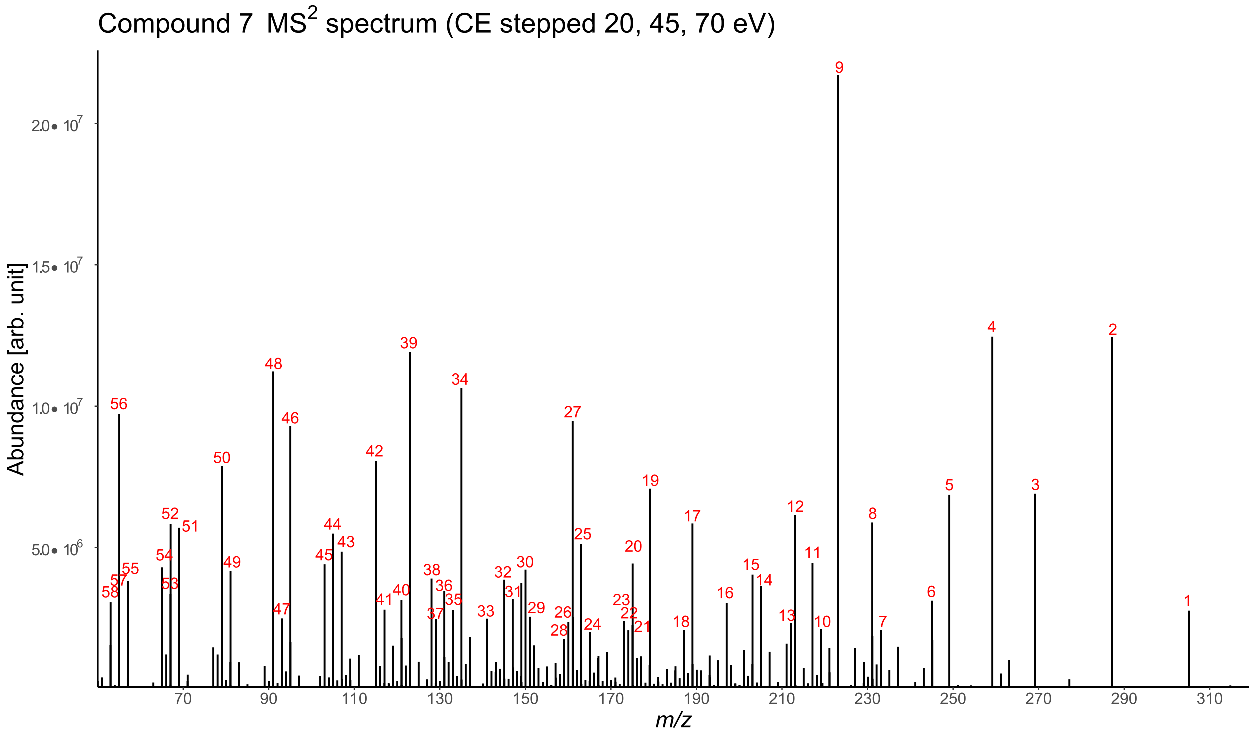

Figure S39 Full MS/MS spectrum of compound 7. The peak assignment is as follows: Peak 1: *m/z* = 305.1747, formula = $C_{18}H_{24}O_4$; Peak 2: *m/z* = 287.1642, formula = $C_{18}H_{22}O_3$; Peak 3: *m/z* = 269.1537, formula = $C_{18}H_{20}O_2$; Peak 4: *m/z* = 259.1694, formula = $C_{17}H_{22}O_2$; Peak 5: *m/z* = 249.1123, formula = $C_{14}H_{16}O_4$; Peak 6: *m/z* = 245.1174, formula = $C_{15}H_{16}O_3$; Peak 7: *m/z* = 233.1174, formula = $C_{14}H_{16}O_3$; Peak 8: *m/z* = 231.1017, formula = $C_{14}H_{14}O_3$; Peak 9: *m/z* = 223.0966, formula = $C_{12}H_{14}O_4$; Peak 10: *m/z* = 219.1017, formula = $C_{13}H_{14}O_3$; Peak 11: *m/z* = 217.1225, formula = $C_{14}H_{16}O_2$; Peak 12: *m/z* = 213.0912, formula = $C_{14}H_{12}O_2$; Peak 13: *m/z* = 212.0833, formula = $C_{14}H_{11}O_2$; Peak 14: *m/z* = 205.1225, formula = $C_{13}H_{16}O_2$; Peak 15: *m/z* = 203.1068, formula = $C_{13}H_{14}O_2$; Peak 16: *m/z* = 197.0598, formula = $C_{13}H_8O_2$; Peak 17: *m/z* = 189.0548, formula = $C_{11}H_8O_3$; Peak 18: *m/z* = 187.0755, formula = $C_{12}H_{10}O_2$; Peak 19: *m/z* = 179.1068, formula = $C_{11}H_{14}O_2$; Peak 20: *m/z* = 175.0755, formula = $C_{11}H_{10}O_2$; Peak 21: *m/z* = 175.0392, formula = $C_{10}H_6O_3$; Peak 22: *m/z* = 174.0677, formula = $C_{11}H_9O_2$; Peak 23: *m/z* = 173.0598, formula = $C_{11}H_8O_2$; Peak 24: *m/z* = 165.0701, formula = $C_{13}H_8$; Peak 25: *m/z* = 163.0391, formula = $C_9H_6O_3$; Peak 26: *m/z* = 161.0962, formula = $C_{11}H_{12}O$; Peak 27: *m/z* = 161.0598, formula = $C_{10}H_8O_2$; Peak 28: *m/z* = 160.0520, formula = $C_{10}H_7O_2$; Peak 29: *m/z* = 151.039, formula = $C_8H_6O_3$; Peak 30: *m/z* = 149.0599, formula = $C_9H_8O_2$; Peak 31: *m/z* = 147.0442, formula = $C_9H_6O_2$; Peak 32: *m/z* =145.0649, formula = $C_{10}H_8O$; Peak 33: *m/z* = 141.0700, formula = $C_{11}H_8$; Peak 34: *m/z* = 135.0442, formula = $C_8H_6O_2$; Peak 35: *m/z* = 133.0650, formula = C_9H_8O; Peak 36: *m/z* = 131.0494, formula = C_9H_6O; Peak 37: *m/z* = 129.0701, formula = $C_{10}H_8$; Peak 38: *m/z* = 128.0623, formula = $C_{10}H_7$; Peak 39: *m/z* = 123.0444, formula = $C_7H_6O_2$; Peak 40: *m/z* = 121.0287, formula = $C_7H_4O_2$; Peak 41: *m/z* = 117.0702, formula = C_9H_8; Peak 42: *m/z* =115.0546, formula =C_9H_6; Peak 43: *m/z* = 107.0496, formula = C_7H_6O; Peak 44: *m/z* = 105.0704, formula =C_8H_8; Peak 45: *m/z* = 103.0547, formula = C_8H_6; Peak 46: *m/z* = 95.0497, formula = C_6H_6O; Peak 47: *m/z* = 93.0704, formula = C_7H_8; Peak 48: *m/z* = 91.0548, formula = C_7H_6; Peak 49: *m/z* = 81.0705, formula = C_6H_8; Peak 50: *m/z* = 79.0549, formula = C_6H_6; Peak 51: *m/z* = 68.9978, formula = C_3O_2; Peak 52: *m/z* = 67.0550, formula = C_5H_6; Peak 53: *m/z* = 67.0186, formula = C_4H_2O; Peak 54: *m/z* = 65.0393, formula = C_5H_4; Peak 55: *m/z* = 57.0707, formula = C_4H_8; Peak 56: *m/z* = 55.0550, formula = C_4H_6; Peak 57: *m/z* = 55.0186, formula = C_3H_2O; Peak 58: *m/z* = 53.0394, formula = C_4H_4.

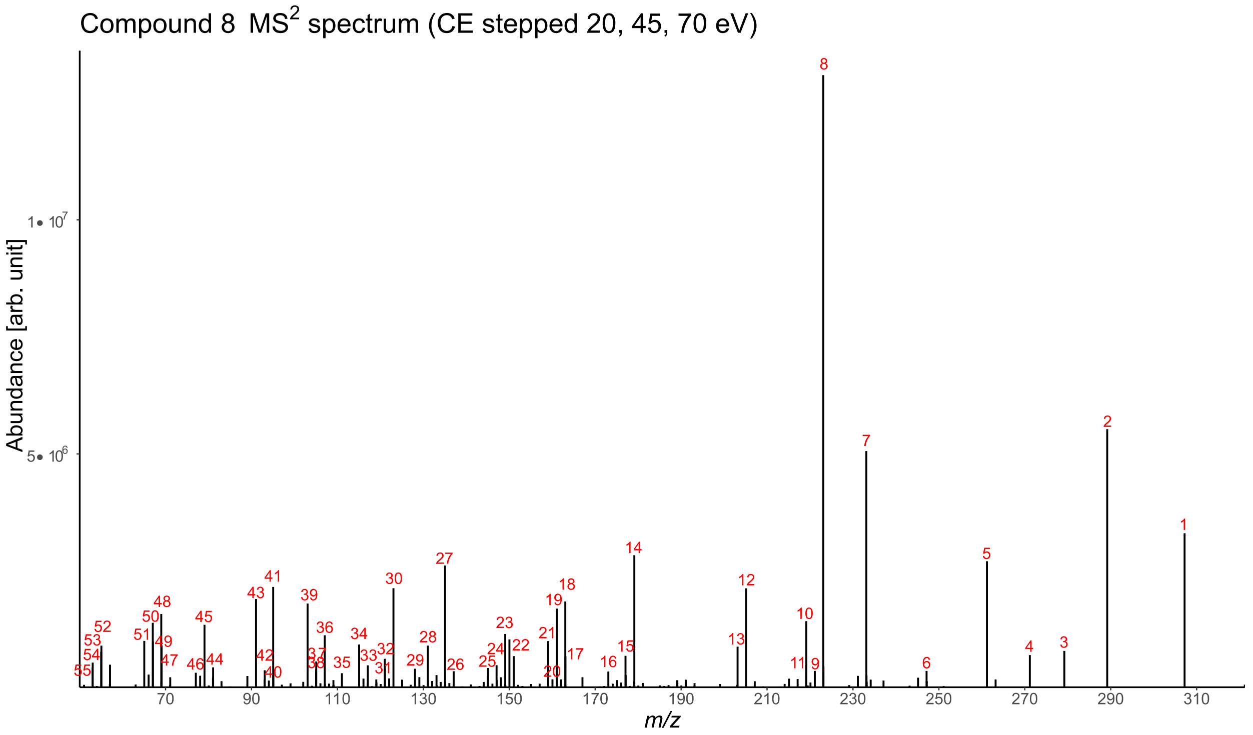

Figure S40 Full MS/MS spectrum of compound 8. The peak assignment is as follows: Peak 1: *m/z* = 307.1902, formula = $C_{18}H_{26}O_4$; Peak 2: *m/z* = 289.1796, formula = $C_{18}H_{24}O_3$; Peak 3: *m/z* = 279.1954, formula = $C_{17}H_{26}O_3$; Peak 4: *m/z* = 271.1691, formula = $C_{18}H_{22}O_2$; Peak 5: *m/z* = 261.1848, formula = $C_{17}H_{24}O_2$; Peak 6: *m/z* = 247.1328, formula = $C_{15}H_{18}O_3$; Peak 7: *m/z* = 233.1172, formula = $C_{14}H_{16}O_3$; Peak 8 : *m/z* = 223.0964, formula = $C_{12}H_{14}O_4$; Peak 9: *m/z* = 221.1172, formula = $C_{13}H_{16}O_3$; Peak 10: *m/z* = 219.1380, formula = $C_{14}H_{18}O_2$; Peak 11: *m/z* = 219.1015, formula = $C_{13}H_{14}O_3$; Peak 12: *m/z* = 205.1223, formula = $C_{13}H_{16}O_2$; Peak 13: *m/z* = 203.1431, formula = $C_{14}H_{18}O$; Peak 14: *m/z* =179.1066, formula = $C_{11}H_{14}O_2$; Peak 15: *m/z* = 177.0547, formula = $C_{10}H_8O_3$; Peak 16: *m/z* = 173.0598, formula = $C_{11}H_8O_2$; Peak 17: *m/z* = 163.0753, formula = $C_{10}H_{10}O_2$; Peak 18: *m/z* = 163.0390, formula = $C_9H_6O_3$; Peak 19: *m/z* = 161.0961, formula = $C_{11}H_{12}O$; Peak 20: *m/z* = 161.0597, formula = $C_{10}H_8O_2$; Peak 21: *m/z* = 159.0441, formula = $C_{10}H_6O_2$; Peak 22: *m/z* = 151.0390, formula = $C_8H_6O_3$; Peak 23: *m/z* = 149.0598, formula = $C_9H_8O_2$; Peak 24: *m/z* = 147.0441, formula = $C_9H_6O_2$; Peak 25: *m/z* = 145.0648, formula = $C_{10}H_8O$; Peak 26: *m/z* = 137.0597, formula = $C_8H_8O_2$; Peak 27: *m/z* = 135.0441, formula = $C_8H_6O_2$; Peak 28: *m/z* = 131.0492, formula = C_9H_6O; Peak 29: *m/z* = 128.0622, formula = $C_{10}H_7$; Peak 30 : *m/z* = 123.0443, formula = $C_7H_6O_2$; Peak 31: *m/z* = 121.0650, formula = C_8H_8O; Peak 32: *m/z* = 121.0286, formula = $C_7H_4O_2$; Peak 33: *m/z* = 117.0701, formula = C_9H_8; Peak 34: *m/z* = 115.0545, formula = C_9H_6; Peak 35: *m/z* = 111.0444, formula = $C_6H_6O_2$; Peak 36: *m/z* = 107.0495, formula = C_7H_6O; Peak 37: *m/z* = 105.0703, formula = C_8H_8; Peak 38: *m/z* =105.0339, formula = C_7H_4O; Peak 39: *m/z* = 103.0546, formula = C_8H_6; Peak 40: *m/z* = 95.0860, formula = C_7H_{10}; Peak 41: *m/z* = 95.0496, formula = C_6H_6O; Peak 42: *m/z* = 93.0704, formula = C_7H_8; Peak 43: *m/z* = 91.0548, formula = C_7H_6; Peak 44: *m/z* = 81.0705, formula = C_6H_8; Peak 45: *m/z* = 79.0548, formula = C_6H_6; Peak 46: *m/z* = 77.0392, formula = C_6H_4; Peak 47: *m/z* = 69.0706, formula = C_5H_8; Peak 48: *m/z* = 68.9978, formula = C_3O_2; Peak 49: *m/z* =67,0549, formula = C_5H_6; Peak 50: *m/z* = 67.0185, formula = C_4H_2O; Peak 51: *m/z* = 65.0393, formula = C_5H_4; Peak 52: *m/z* = 55.0550, formula = C_4H_6; Peak 53: *m/z* = 55.0186, formula = C_3H_2O; Peak 54: *m/z* = 53.0393, formula = C_4H_4; Peak 55: *m/z* = 53.0030, formula =C_3O.

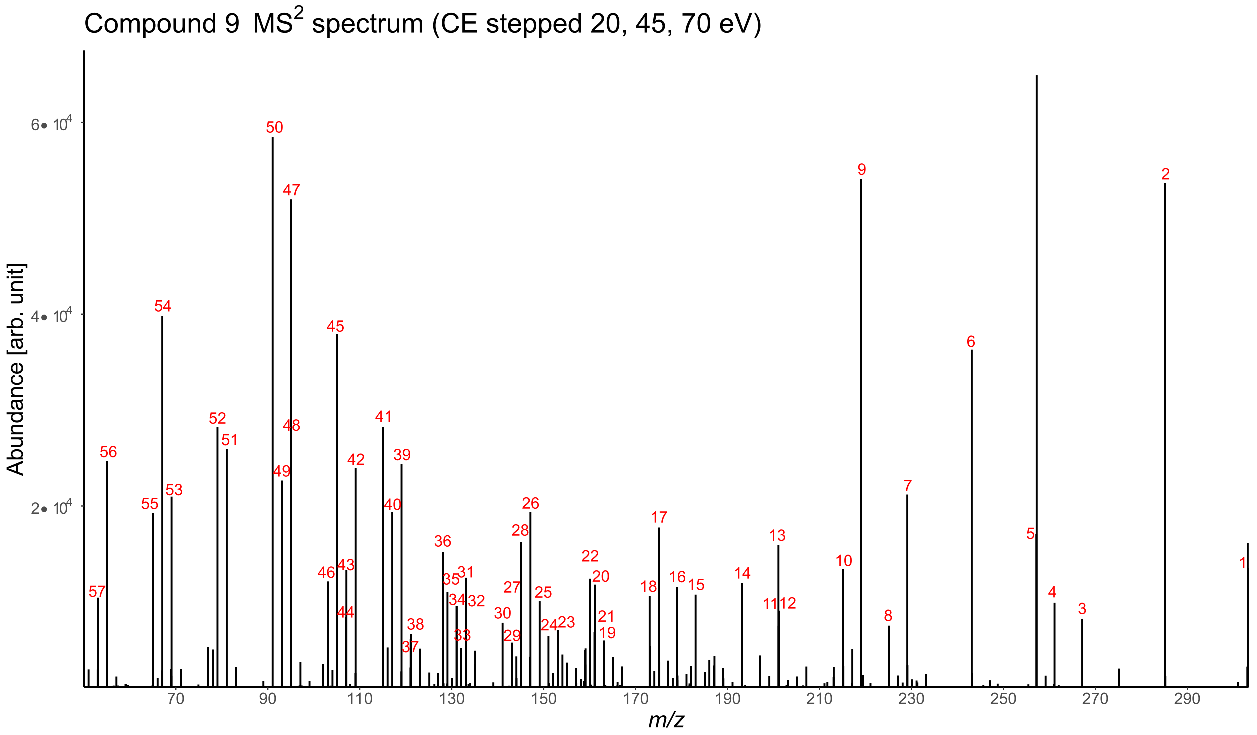

Figure S41 Full MS/MS spectrum of compound 9. The peak assignment is as follows: Peak 1: *m/z* = 303.1587, formula = $C_{18}H_{22}O_4$; Peak 2: *m/z* = 285.1484, formula = $C_{18}H_{20}O_3$; Peak 3: *m/z* = 267.1379, formula = $C_{18}H_{18}O_2$; Peak 4: *m/z* = 261.1121, formula = $C_{15}H_{16}O_4$; Peak 5: *m/z* = 257.1534, formula = $C_{17}H_{20}O_2$; Peak 6: *m/z* = 243.1016, formula = $C_{15}H_{14}O_3$; Peak 7: *m/z* = 229.0860, formula = $C_{14}H_{12}O_3$; Peak 8: *m/z* = 225.0911, formula = $C_{15}H_{12}O_2$; Peak 9: *m/z* = 219.0652, formula = $C_{12}H_{10}O_4$; Peak 10: *m/z* =215.1067, formula = $C_{14}H_{14}O_2$; Peak 11: *m/z* = 201.1638, formula = $C_{15}H_{20}$; Peak 12: *m/z* = 201.0910, formula = $C_{13}H_{12}O_2$; Peak 13: *m/z* = 201.0545, formula = $C_{12}H_8O_3$; Peak 14: *m/z* = 193.1223, formula = $C_{12}H_{16}O_2$; Peak 15: *m/z* = 183.0441, formula = $C_{12}H_6O_2$; Peak 16: *m/z* = 179.0339, formula = $C_9H_6O_4$; Peak 17: *m/z* = 175.0754, formula = $C_{11}H_{10}O_2$; Peak 18: *m/z* = 173.0598, formula = $C_{11}H_8O_2$; Peak 19: *m/z* = 163.1481, formula = $C_{12}H_{18}$; Peak 20: *m/z* = 161.1325, formula = $C_{12}H_{16}$; Peak 21: *m/z* = 161.0598, formula = $C_{10}H_8O_2$; Peak 22: *m/z* = 160.0519, formula = $C_{10}H_7O_2$; Peak 23: *m/z* = 153.0699, formula = $C_{12}H_8$; Peak 24: *m/z* = 151.0390, formula = $C_8H_6O_3$; Peak 25: *m/z* = 149.1326, formula = $C_{11}H_{16}$; Peak 26: *m/z* = 147.1168, formula = $C_{11}H_{14}$; Peak 27: *m/z* = 145.1013, formula = $C_{11}H_{12}$; Peak 28: *m/z* = 145.0648, formula = $C_{10}H_8O$; Peak 29: *m/z* = 143.0855, formula = $C_{11}H_{10}$; Peak 30: *m/z* = 141.0699, formula = $C_{11}H_8$; Peak 31: *m/z* = 133.1013, formula = $C_{10}H_{12}$; Peak 32: *m/z* = 133.0649, formula = C_9H_8O; Peak 33: *m/z* = 131.0856, formula = $C_{10}H_{10}$; Peak 34: *m/z* = 131.0493, formula = C_9H_6O; Peak 35: *m/z* = 129.0701, formula = $C_{10}H_8$; Peak 36: *m/z* = 128.0622, formula = $C_{10}H_7$; Peak 37: *m/z* = 121.1013, formula = C_9H_{12}; Peak 38: *m/z* = 121.0650, formula = C_8H_8O; Peak 39: *m/z* = 119.0858, formula = C_9H_{10}; Peak 40: *m/z* = 117,0702, formula = C_9H_8; Peak 41: *m/z* = 115.0545, formula = C_9H_6; Peak 42: *m/z* = 109.1016, formula = C_8H_{12}; Peak 43: *m/z* = 107.0859, formula = C_8H_{10}; Peak 44: *m/z* = 107.0495, formula = C_7H_6O; Peak 45: *m/z* = 105.0703, formula = C_8H_8; Peak 46: *m/z* = 103.0547, formula = C_8H_6; Peak 47: *m/z* = 95.0861, formula = C_7H_{10}; Peak 48: *m/z* = 95.0496, formula = C_6H_6O; Peak 49: *m/z* = 93.0704, formula = C_7H_8; Peak 50: *m/z* = 91.0548, formula = C_7H_6; Peak 51: *m/z* = 81.0705, formula = C_6H_8; Peak 52: *m/z* = 79.0549, formula = C_6H_6; Peak 53: *m/z* = 69.0706, formula = C_5H_8; Peak 54: *m/z* = 67.0549, formula = C_5H_6; Peak 55: *m/z* = 65.0393, formula = C_5H_4; Peak 56: *m/z* = 55.0550, formula = C_4H_6; Peak 57: *m/z* = 53.0393, formula = C_4H_4.

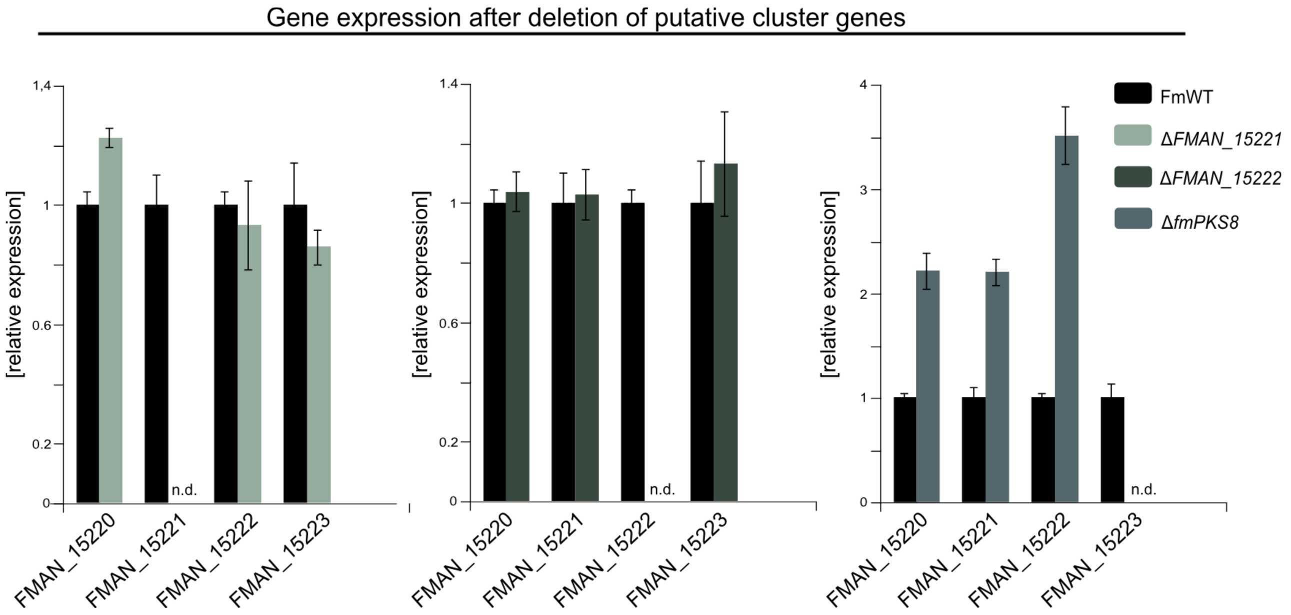

Figure S42 Expressional analysis of putative cluster genes involved in fusamarin biosynthesis. *F. mangiferae* (FmWT), Δ*FMAN_15221*, Δ*FMAN_15222* and Δ*fmPKS8* deletion strains were cultivated under *FmPKS8*-inducing conditions for 3 days at 30°C in the dark. Gene expression was determined after RNA extraction and cDNA synthesis using RT-qPCR. Lack of FMAN_15221, FMAN_15222 nor FmPKS8 influenced gene expression of the remaining cluster genes. For data normalization, the house-keeping genes actin, GPD and β-tubulin were used. Primers are listed in Supplementary Table 2. The FmWT gene expression was arbitrarily set to 1. Mean values and standard deviations are shown. n.d., not detected; RE, relative expression.

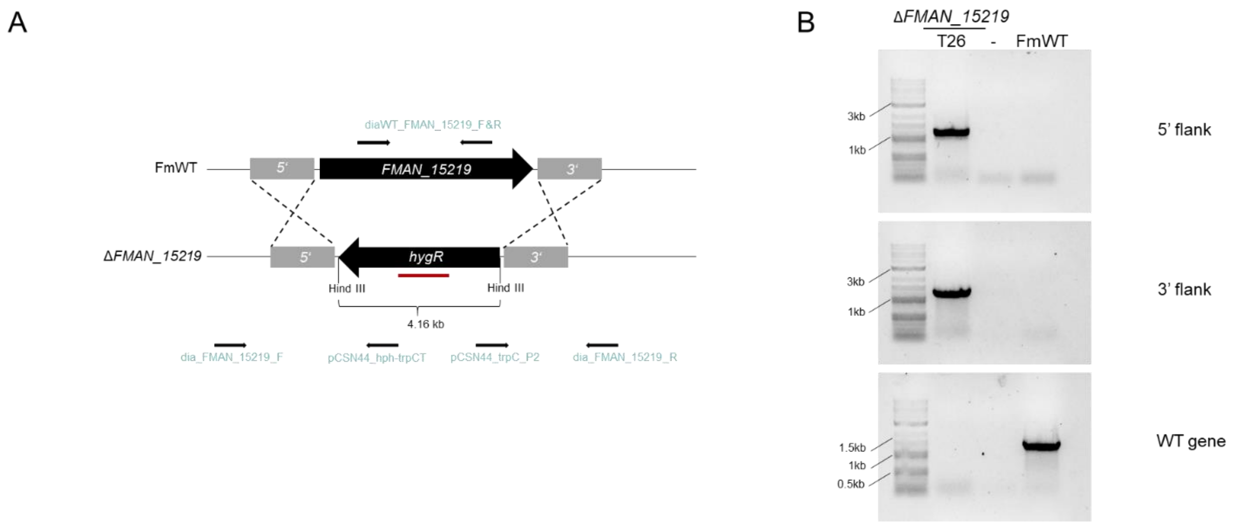

Figure S43 Deletion strategy and verification of Δ*FMAN_15219* deletion by diagnostic PCR. (A) Deletion of *FMAN_15219 via* homologous recombination. Primer used for cloning of the gene deletion cassette and for diagnostic PCRs are depicted in turquoise. (B) Verification of successful gene deletion by the replacement of the native gene against a hygromycin cassette. Homologous integration was verified by the presence of the upstream (5') and downstream (3') region with the primer pairs dia_FMAN_15219_F/pCSN44_hph-trpC-T and dia_FMAN_15219_R/ pCSN44_trpC_P2, respectively. Removal of the wild-type gene was verified with the primer pairs diaWT_FMAN_15219_F/R for the independent mutant Δ*FMAN_15219*_T26. As size marker 1 kb Plus DNA ladder (NEB) was used. As negative (-) control for the 5' and 3' flank sterile IonEx was used, as well as *F. mangiferae* (FmWT) gDNA. For the amplification of the wild-type gene FmWT gDNA served as positive control.

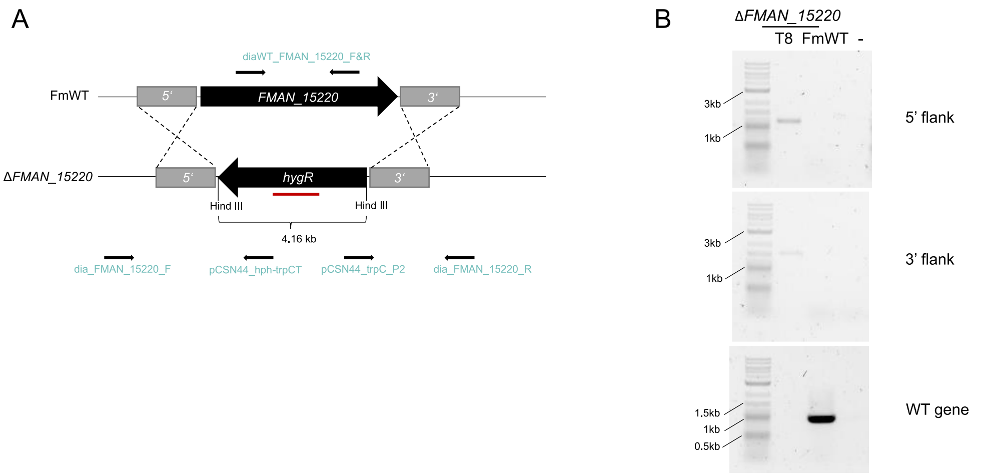

Figure S44 Deletion strategy and verification of Δ*FMAN_15220* deletion by diagnostic PCR. (A) Gene deletion of *FMAN_15220* by homologous recombination. Primers used for cloning of the gene deletion cassette as well as for diagnostic PCRs for the verification of successful homologous recombination events are shown in turquoise. (B) Homologous recombination events and presence of the hygromycin cassette were verified by diagnostic PCRs. Presence of the upstream region (5') was tested with the primer pair dia_FMAN_15220_F/pCSN44_hph-trpC-T, while for the downstream region (3') the primer pair dia_FMAN_15220_R/ pCSN44_trpC_P2 was used. Absence of wild-type gene was verified with the primer pairs diaWT_FMAN_15220_F/R for the independent transformant Δ*FMAN_15220*_T8. The 1 kb Plus DNA ladder (NEB) was used as a size marker. Sterile IonEX and FmWT gDNA served as a negative (control) for the 5' and 3' flank. Amplification of the wild-type gene was verified using *F. mangiferae* (FmWT) gDNA as a positive control.

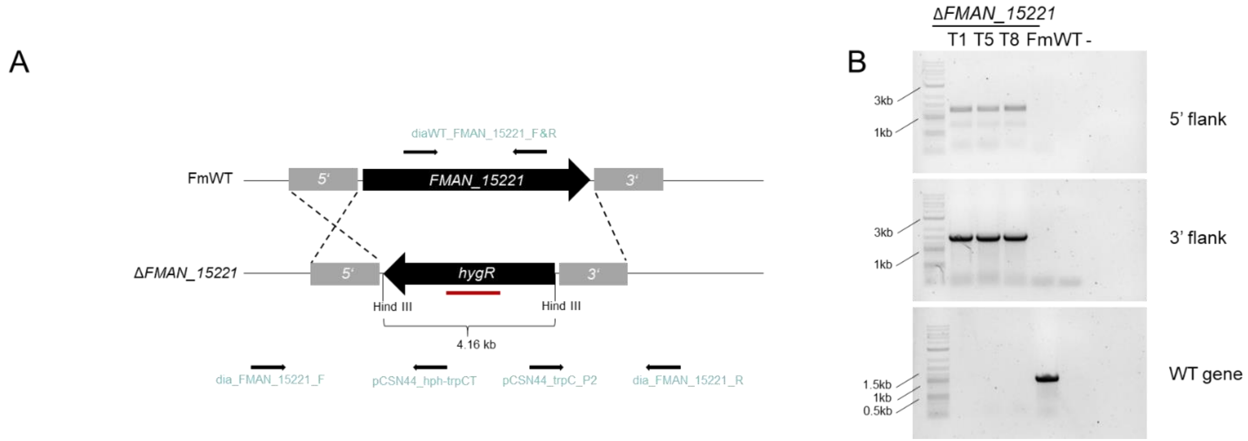

Figure S45 Deletion strategy and verification of Δ*FMAN_15221* deletion by diagnostic PCR. (A) Targeted gene deletion of *FMAN_15221* was performed *via* homologous recombination. Primer pairs used for yeast recombinational cloning of the deletion cassette and diagnostic PCRs are shown in turquoise. (B) Verification of gene deletion by homologous recombination against a hygromycin resistance cassette. Successful gene deletion was verified using the primer pairs dia_FMAN_15221_F/pCSN44_hph-trpC-T for the amplification of the upstream (5') region and dia_FMAN_15221_R/ pCSN44_trpC_P2 for the amplification of the downstream region (3'). Removal of the wild-type gene was tested with the primer pairs diaWT_FMAN_15221_F/R for Δ*FMAN_15221*_T1, T5 and T8. The 1 kb Plus DNA ladder (NEB) was used as size marker. As negative (-) either FmWT gDNA or sterile IonEx was used. For the amplification of the wild-type gene *F. mangiferae* (FmWT) gDNA served as positive control.

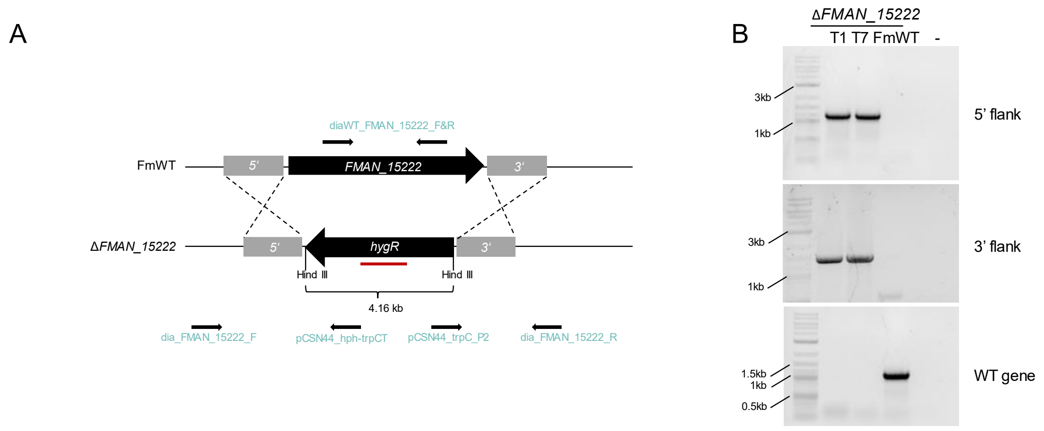

Figure S46 Deletion strategy and verification of Δ*FMAN_15222* deletion by diagnostic PCR. (A) Deletion and verification strategy by homologous recombination of *FMAN_15222*. Primer pairs used for the cloning of the deletion cassette as well as for diagnostic PCRs are depicted in turquoise. (B) Verification of homologous recombination *via* diagnostic PCRs. Primer pairs used for cloning of the deletion cassette and diagnostic PCRs are depicted in turquoise. Verification of gene deletion was performed by the amplification of the upstream (5') region using the primer pairs dia_FMAN_15222_F/pCSN44_hph-trpC-T and dia_FMAN_15222_R/ pCSN44_trpC_P2 for the amplification of the downstream region (3'). Absence of the wild type gene was verified with the primer pairs diaWT_FMAN_15222_F/R for Δ*FMAN_15222*_T1 and T7. The 1 kb Plus DNA ladder (NEB) was used as size marker. *F. mangiferae* (FmWT) gDNA or sterile IonEX served as negative (-) control. For the amplification of the wild-type gene FmWT gDNA was used as positive control.

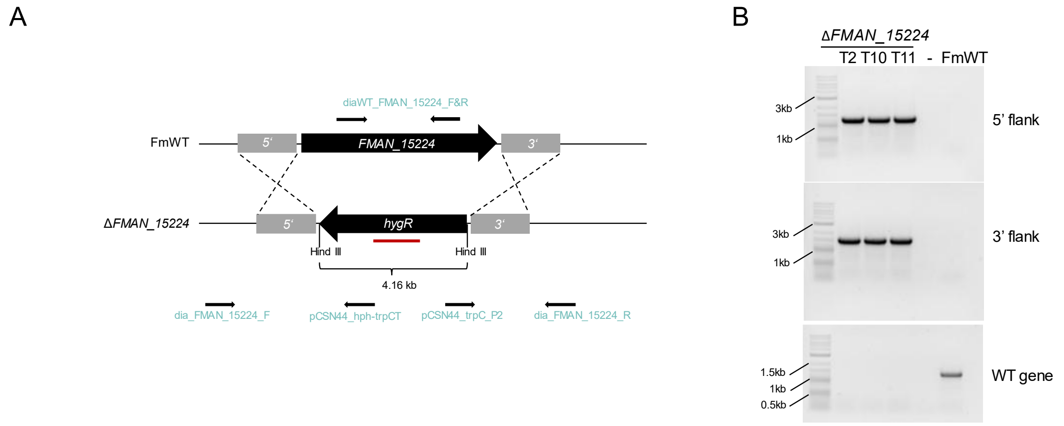

Figure S47 Deletion strategy and verification of Δ*FMAN_15224* deletion by diagnostic PCR. (A) Deletion strategy of *FMAN_15224 via* homologous recombination. Primer pairs used for cloning of the linear deletion cassette and verification via diagnostic PCRs are shown in turquoise. (B) Verification of homologous recombination by the exchange of the native gene against a hygromycin cassette by diagnostic PCRs. For the amplification of the upstream (5') region primer pairs dia_FMAN_15224_F/pCSN44_hph-trpC-T and for the downstream (3') region primer pairs dia_FMAN_15222_R/ pCSN44_trpC_P2 are used. Removal of the of the wild type gene was verified with the primer pairs diaWT_FMAN_15224_F/R for Δ*FMAN_15224*_T2, T10 and T11. The 1 kb Plus DNA ladder (NEB) was used as size marker. *F. mangiferae* (FmWT) gDNA or sterile IonEX served as negative (-) control. For the amplification of the wild-type gene FmWT gDNA was used as positive control.

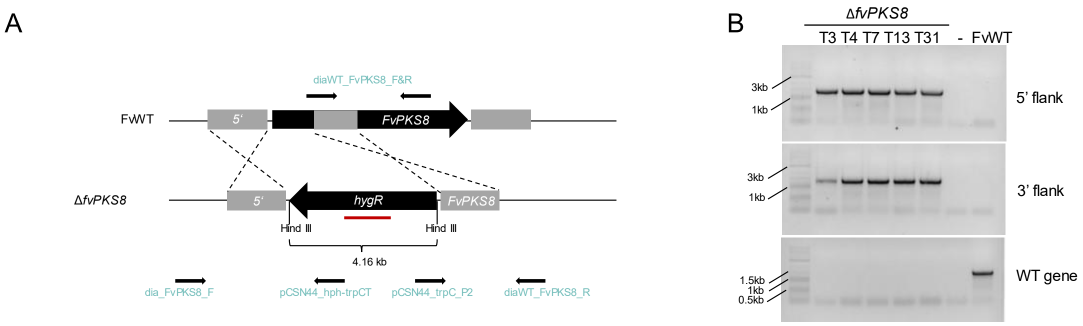

Figure S48 Deletion strategy and verification of Δ*fvPKS8* partial gene deletion by diagnostic PCR. (A) Partial gene deletion strategy of *fvPKS8 via* homologous recombination. Primer pairs used for yeast recombinational cloning of the linear deletion cassette and verification *via* diagnostic PCRs are shown in turquoise. (B) Verification of the partial gene deletion of the wild type gene and integration of a hygromycin resistance cassette by diagnostic PCRs. The upstream gene (5') was amplified using the primer pairs dia_FvPKS8_F/pCSN44_hph-trpC-T and the downstream gene (3') was amplified using the primer pair diaWT_FvPKS8_R/ pCSN44_trpC_P2. Absence of the wild type gene was tested using the primers diaWT_FvPKS8_F/R for Δ*fvPKS8*_T3, T4, T7, T13 and T31. The 1 kb Plus DNA ladder (NEB) was used as size marker. *F. verticillioides* (FvWT) gDNA or sterile IonEX served as negative (-) control. For the amplification of the wild-type gene FvWT gDNA was used as positive control.

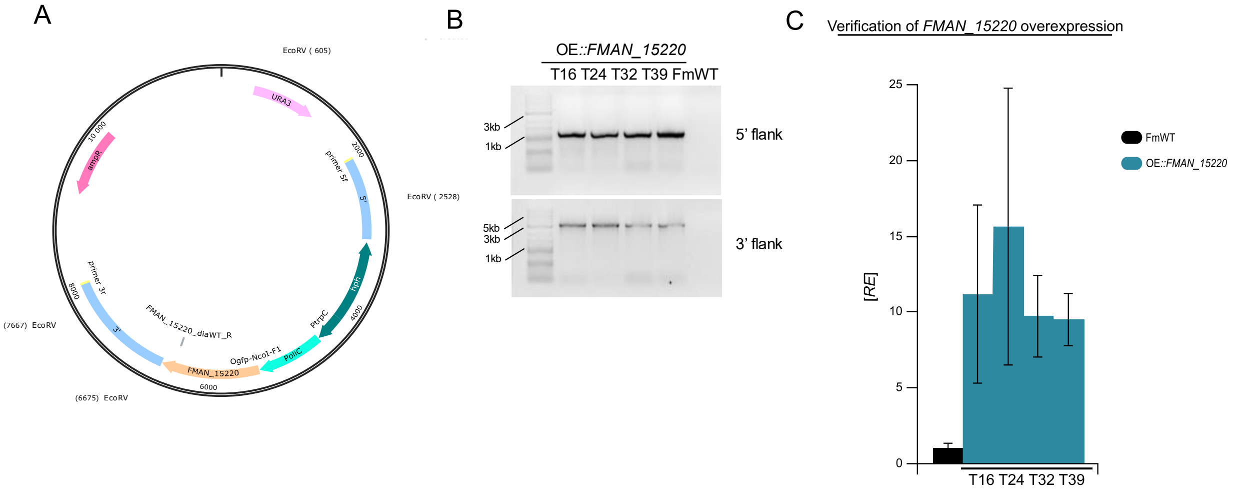

Figure S49 Overexpression strategy and verification of OE*::FMAN_15220*. (A) Strategy for the *in loco* overexpression of *FMAN_15220*. Enzyme used for the restriction digest is depicted. (B) Verification of the in loco integration of the *FMAN_15220* gene under the strong constitutive oliC promoter from *Aspergillus nidulans via* diagnostic PCR. For the amplification of the upstream (5') region the primer pairs dia_FMAN_15220_F/pCSN44_hph-trpC-T are used, while for the amplification of the downstream region (3') the primer pairs dia_FMAN_15220_F2/pOliC_F2 are used. Overall four FMAN_15220 overexpression mutants were obtained i.e., OE*::FMAN_15220*_T16, T24, T32, T39. The 1 kb Plus DNA ladder (NEB) was used as size marker. *F. mangiferae* (FmWT) gDNA or sterile IonEX served as negative (-) control. For the amplification of the wild-type gene FmWT gDNA was used as positive control. (C) Determination of *FMAN_15220* transcript levels in the OE*::FMAN_15220* mutant strains. To evaluate suitability of the gained overexpression strains, *FMAN_15220* transcript levels were assessed by RT-qPCR after 3 days of growth in PKS8-inducing conditions. Data represent mean values (n = x).

Table S1: Metabolomic comparison of different strains. Metabolites missing in Δ*fmPKS8* and Δ*fmPPT1* metabolite profile compared to FmWT.

predicted formula	mass [Da]	retention time [min]	max. peak area [arb. units]
$C_{18} H_{24} O_5$	320.16206	28.264	1.18E+07
$C_{18} H_{20} O_4$	300.13597	27.522	8.97E+07
$C_{16} H_{20} O_7 S$	356.09294	26.466	7.04E+07
$C_{18} H_{26} O_5$	322.17792	24.401	4.15E+07
$C_{18} H_{24} O_5$	320.16226	23.44	3.30E+07
$C_{19} H_{20} N_4 O_3 S$	384.12414	29.503	1.89E+08
$C_{18} H_{31} O_2 P_3$	372.15454	29.251	3.26E+07
$C_{18} H_{22} O_7 S$	382.10856	28.118	4.70E+08
$C_{12} H_{16} O_4$	224.10454	19.154	8.41E+07
$C_{20} H_{26} O_4$	330.18297	30.153	1.06E+07
$C_{18} H_{20} O_7 S$	380.09283	27.039	7.96E+07
$C_{10} H_{14} O_3$	182.09346	19.459	1.26E+07
$C_{18} H_{24} O_4$	304.16722	29.252	3.19E+09
$C_{18} H_{24} O_7 S$	384.12404	29.736	4.53E+08
$C_{18} H_{22} O_4$	302.15164	30.718	4.49E+08
$C_{18} H_{26} O_4$	306.18301	29.929	4.51E+08
$C_{18} H_{22} O_7 S$	382.10843	28.294	6.35E+08
$C_{18} H_{22} O_4$	302.15163	28.353	7.67E+08

Table S2: Overview of primers used in this work. Introduced overhangs required for yeast recombinational cloning are written in uppercase letters. Primers are designed with * also bind in *F. mangiferae* genome. Primer designed with α also bind and *F. verticillioides* M3125 genome.

Gene ID	Primer ID	Primer sequence
**Primers for plasmid construction and fragment amplification**		
FMAN_15219	FMAN_15219_5F	GCCAGGGTTTTCCCAGTCACGACGgctccggtgcaaaatattgaagacc
	FMAN_15219_5R	CTTAACGTTACTGAAATCTCCAACggagcgtcgcacagtttagatatag
	FMAN_15219_3F	TTCAATATCATCTTCTGTCTCCGACtcgttcaggctgtataccagctac
	FMAN_15219_3R	TAACAATTTCACACAGGAAACAGCctcacctgcattagtaactatgcgc
FMAN_15220	FMAN_15220_5F	CGCCAGGGTTTTCCCAGTCACGACGcgactgtgacagctagagagaagc
	FMAN_15220_5R	ACTTAACGTTACTGAAATCTCCAACttgtctctggcccaaagcgtaagg
	FMAN_15220_3F	TCAATATCATCTTCTGTCTCCGACagtaaagtaggttgcggttacagg
	FMAN_15220_3R	ATAACAATTTCACACAGGAAACAGCcgcagtatatacaagctcacagtcg
	OE-FMAN_15220_F	caatcgatccaaccatgatgcgatgccgacacgtc
/	OliC_R1	ggttggatcgattgtgatgtgatgg
/	TtrpC-hph_R	cccggggttaacgttaactggttcc
FMAN_15221	FMAN_15221_5F	CGCCAGGGTTTTCCCAGTCACGACGgatggtgtttatggacggtctagc
	FMAN_15221_5R	ACTTAACGTTACTGAAATCTCCAACatgagtatgaatgcgactgtgagc
	FMAN_15221_3F	TTCAATATCATCTTCTGTCTCCGACgtgccattcttggtctgtcttgtt
	FMAN_15221_3R	ATAACAATTTCACACAGGAAACAGCcgtcccagaatattgatccagacc
FMAN_15222	FMAN_15222_5F	CGCCAGGGTTTTCCCAGTCACGACGtgatactgtggtaggctgtcaagg
	FMAN_15222_5R	CTTAACGTTACTGAAATCTCCAACggatgacatactgaacgatgacgag
	FMAN_15222_3F	TCAATATCATCTTCTGTCTCCGACggcgaacgaaatggccttaatttca
	FMAN_15222_3R	ATAACAATTTCACACAGGAAACAGCcgttgaagttcaatggcgattcga
FMAN_15224	FMAN_15224_5F	CGCCAGGGTTTTCCCAGTCACGACGtcagtggactgggctatgttcagc

	FMAN_15224_5R	ACTTAACGTTACTGAAATCTCCAACcaagttgttctgtctgttgacgcc
	FMAN_15224_3F	TCAATATCATCTTCTGTCTCCGACcctaagaacccctcacattggttgc
	FMAN_15224_3R	TAACAATTTCACACAGGAAACAGCtcgcttactggatggagacattcgg
FVEG_10497	FvPKS8_5F	GCCAGGGTTTTCCCAGTCACGACGactgacaatttcagaagctgggtgg
	FvPKS8_5R	CTTAACGTTACTGAAATCTCCAACgagtgatctctaatgcgtgcgttcc
	FmPKS8_3F	TCAATATCATCTTCTGTCTCCGACtctcattgaagaactccgtgctcc
	FmPKS8_3R	AACAATTTCACACAGGAAACAGCacgaaggacttgtttccagtgaacc
/	Split-mark_hphF	cgttgcaagacctgcctgaa
/	Split-mark_hphR	ggatgcctccgctcgaagta
**Primers used for diagnostic PCR**		
FMAN_15219	dia_FMAN_15219_F	cagtagagccatatgggtatcgc
	dia_FMAN_15219_R	aacaggcttaccatatgcacgc
	diaWT_FMAN_15219_F	catcacactcggagttatggttgc
	diaWT_FMAN_15219_R	gccagagttagtcatgatcgtcc
FMAN_15220	dia_FMAN_15220_F	atgctcaacataggactgcc
	dia_FMAN_15220_R	cgtcttacgtgactcaaagg
	diaWT_FMAN_15220_F	gtgtgtacaactgtatggtcg
	diaWT_FMAN_15220_R	tctctgtacgtgtcagatcc
FMAN_15221	dia_FMAN_15221_F	catgctcaccagccaacaactcc
	dia_FMAN_15221_R	tgctccaaatgtgtcaaggc
	diaWT_FMAN_15221_F	cacctttaaaacactcgatggcc
	diaWT_FMAN_15221_R	tccataacagagtcgacatcgcc
FMAN_15222	dia_FMAN_15222_F	tgctgtatcaatacccacagacgg

	dia_FMAN_15222_R2	catcagacatttccgaccgtgc
	diaWT_FMAN_15222_F	atgatgctagttcgcgttgagg
	diaWT_FMAN_15222_R	gataacaccgtctcgtagatcctg
FMAN_15224	dia_FMAN_15224_F	ctgcatatggctcatcgaattgg
	dia_FMAN_15224_R	aagtgtgagacgaatcagcagc
	diaWT_FMAN_15224_F	tcttccctggttattgcgatcg
	diaWT_FMAN_15224_R	gttgaagaagatcgcatagcagg
FVEG_10497	dia_FvPKS8_F	cgcacgttgcagagttatagtcg
	diaWT_FvPKS8_F	tcctaaacaagatccctcgcacc
	diaWT_FvPKS8_R	ttgtgacatagcacgttgctgg
/	pCSN44-hph-trpC-T	ggaatagagtagatgccgaccgg
/	pCSN44-trpC_P2	gtgatccgcctggacgactaaacc
/	pOliC_F2	gaaactcagtctccttgg
**Primers used for sequencing**		
/	pRS426_seqF	gccattcaggctgcgcaactg
/	hph-hiF	gtctggaccgatggctgtgtagaag
/	TtrpC-hph_R	cccggggttaacgttaactggttcc
/	ptrpC-hph_F	gatattgaaggagcattttttggg
/	pCSN44-trpC-P2	gtgatccgcctggacgactaaacc
/	pOliC-F2	gaaactcagtctccttgg
/	pRS426_seq_R	gttgtgtggaattgtgagcgg
FMAN_15220	diaWT_FMAN_15220_R	tctctgtacgtgtcagatcc
	FMAN_15220_3F	tcaatatcatcttctgtctccgacagtaaagtaggttgcggttacagg

	FMAN_15220_F	gctccaccaactgtctcgtcac
**Primers for semi-quantitative and RT-qPCR**		
*cDNA verification**		
FFUJ_02611	cDNA_check_Actin_F	gtatgtgcaaggccggtttcg
	cDNA_check_Actin_R	gagaccagggtacatggtgg
*Housekeeping genes**		
FFUJ_02611	Actin_F	ccaccatgtaccctggtctctcc
	Actin_R	aatggaaccaccgatccagacgg
FFUJ_07385	ß-TUB_F	gaggcagtacgatggcatgcg
	ß-TUB_R	ggtaatctgcgtcttcagcagcttcg
FFUJ_13490	GPD_F	gcctctgagggtgacctcaagg
	GPD_R	cgttgtcgtaccaggagaccagc
Cluster genes		
FMAN_15219	FMAN_15219_F	gaggtgtcgaagatgtgccagg
	FMAN_15219_R	gcgaggcaagactgtactgagc
FMAN_15220	FMAN_15220_F	gctccaccaactgtctcgtcac
	FMAN_15220_R	gaagatgctgctgacaggcaagg
FMAN_15221	FMAN_15221_F	tctgccctacgtcaacgaagatgg
	FMAN_15221_R	tcaccatcattgcaggcgtgg
FMAN_15222	FMAN_15222_F	atcccgtcatagtcctcgtggc
	FMAN_15222_R	tagtcaaagcaggctgaggctcc
FMAN_15223	FmPKS8_F	ctccagtccgaggacgacagc
	FmPKS8_R	acattggtgtcaagctcctctggc

FMAN_15224	FMAN_15224_F	tcccgattctacggcgatgg
	FMAN_15224_R	gtgggaagatctcagcgacaacg
PKS genes		
FMAN_01974	FmPKS2_F	gctgatggcggttgagttgagg
	FmPKS2_R	cgagcttcgcatctcagcgac
FMAN_08330	FmPKS5_F	gatccgggtttcaccatgccg
	FmPKS5_R	tctgctgctctatccagagccg
FMAN_09265	FmPKS7_F	tgtctacggtgttgactccctgg
	FmPKS7_R	ccaaaaccgcctcgtctagcg
FMAN_15223	FmPKS8_F	ctccagtccgaggacgacagc
	FmPKS8_R[α]	acattggtgtcaagctcctctggc
FVEG_10497	FvPKS8_F	gcttgtggcattacctccaggc
FMAN_06335	FmPKS9_F	ctgctaccgagactgccgatga
	FmPKS9_R[α]	ccgtgccattcagacctgttgg
FMAN_13212	FmPKS12_F	cacttactgcgattgagttgcggc
	FmPKS12_R	caaccgtctctcaacgacggtgc
FMAN_13912	FmPKS14_F	cagccacggtgtactcaacga
	FmPKS14_R	tgacagagccgagggaatgc
FMAN_15195	FmPKS17_F	atggtgtcgactcactggttgc
	FmPKS17_R	aatctcgctagccaacgctcg
FMAN_15205	FmPKS18_F	ccgcatcccgtctaccatatcg
	FmPKS18_R	tacgcaccagctttacccatcg
FMAN_11871	FmPKS20_F	gatgcactcaacgctctgcttcg

	FmPKS20_R	ttgaacgtcacgcccaagtgg
FMAN_02337	FmPKS38_F	ccttcagacacatgcagcaaggc
	FmPKS38_R	tggagatcgggtttcctagtggc
FMAN_09800	FmPKS43_F	tgcttaagcctgtggaggaccttg
	FmPKS43_R	ccaaggacgtccagcgtcc

Table S3 Non-covalent intermolecular interactions in compound **6** (Å and deg)

D-H···*A*	*d*(*D*-H)	*d*(H···*A*)	*d*(*D*···*A*)	∠(*DHA*)
C3-H3A···O7$^{\#1}$	1.00	2.560	3.359(6)	136.9
C4-H4A···O8$^{\#2}$	0.99	2.620	3.495(7)	147.5
C5-H5···O5$^{\#3}$	0.95	2.520	3.229(6)	131.9
O3-H3···O5$^{\#3}$	0.87(7)	1.88(7)	2.747(5)	176.0(6)
O4-H4···O1	0.86(9)	1.80(9)	2.607(5)	154.0(8)
O7-H7···O1	0.93(3)	1.80(3)	2.723(5)	179.0(8)
O8-H8···O5	0.91(7)	1.74(7)	2.594(5)	155.0(6)
C24-H24B···O5$^{\#1}$	0.99	2.620	3.409(7)	136.4
C25-H25···O1	0.95	2.560	3.264(6)	130.6

Symmetry transformations used to generate equivalent atoms: $^{\#1}$ x+1, y, z; $^{\#2}$ x+1, y+1, z; $^{\#3}$ x+2, y+1, z.

Table S4: Table of fragmented ions of compounds **6**-**9**

Fragmented ions of compounds **6**-**9**
$C_{18}H_{22}O_4$, $C_{18}H_{20}O_3$, $C_{18}H_{18}O_2$, $C_{15}H_{16}O_4$, $C_{17}H_{20}O_2$, $C_{15}H_{14}O_3$, $C_{14}H_{14}O_3$, $C_{15}H_{12}O_2$, $C_{12}H_{12}O_4$, $C_{14}H_{16}O_2$, $C_{14}H_{14}O_2$, $C_{13}H_{14}O_2$, $C_{13}H_{12}O_2$, $C_{12}H_8O_3$, $C_{14}H_{12}O$, $C_{10}H_8O_4$, $C_{11}H_8O_3$, $C_{12}H_{10}O_2$, $C_{13}H_9O$, $C_{13}H_8O$, $C_{14}H_{10}$, $C_9H_6O_4$, $C_{11}H_{12}O_2$, $C_{11}H_{10}O_2$, $C_{10}H_6O_3$, $C_{11}H_8O_2$, $C_{13}H_{12}$, $C_8H_6O_4$, $C_{13}H_8$, $C_{10}H_8O_2$, $C_{10}H_7O_2$, $C_{11}H_8O$, $C_{12}H_9$, $C_{12}H_8$, $C_{12}H_7$, $C_8H_6O_3$, $C_9H_6O_2$, $C_{10}H_8O$, $C_{11}H_8$, $C_8H_6O_2$, C_9H_8O, C_9H_6O, $C_{10}H_8$, $C_{10}H_7$, C_8H_6O, C_9H_8, C_9H_6, C_8H_8, C_8H_6, C_6H_6O, C_7H_6, C_6H_8, C_6H_6, C_4H_4O, C_5H_6, C_5H_4, C_4H_6, C_3H_2O, C_4H_4, $C_{18}H_{24}O_4$, $C_{18}H_{22}O_3$, $C_{18}H_{20}O_2$, $C_{17}H_{22}O_2$, $C_{14}H_{16}O_4$, $C_{15}H_{16}O_3$, $C_{14}H_{16}O_3$, $C_{12}H_{14}O_4$, $C_{13}H_{14}O_3$, $C_{14}H_{12}O_2$, $C_{14}H_{11}O_2$, $C_{13}H_{16}O_2$, $C_{13}H_8O_2$, $C_{11}H_{14}O_2$, $C_{11}H_9O_2$, $C_9H_6O_3$, $C_{11}H_{12}O$, $C_9H_8O_2$, $C_7H_6O_2$, $C_7H_4O_2$, C_7H_6O, C_7H_8, C_3O_2, C_4H_2O, C4H8, $C_{18}H_{26}O_4$, $C_{18}H_{24}O_3$, $C_{17}H_{26}O_3$, $C_{18}H_{22}O_2$, $C_{17}H_{24}O_2$, $C_{15}H_{18}O_3$, $C_{13}H_{16}O_3$, $C_{14}H_{18}O_2$, $C_{14}H_{18}O$, $C_{10}H_8O_3$, $C_{10}H_{10}O_2$, $C_{10}H_6O_2$, $C_8H_8O_2$, C_8H_8O, $C_6H_6O_2$, C_7H_4O, C_7H_{10}, C_6H_4, C_5H_8, C_3O, $C_{14}H_{12}O_3$, $C_{12}H_{10}O_4$, $C_{15}H_{20}$, $C_{12}H_{16}O_2$, $C_{12}H_6O_2$, $C_{12}H_{18}$, $C_{12}H_{16}$, $C_{11}H_{16}$, $C_{11}H_{14}$, $C_{11}H_{12}$, $C_{11}H_{10}$, $C_{10}H_{12}$, $C_{10}H_{10}$, C_9H_{12}, C_9H_{10}, C_8H_{12}, C_8H_{10}

Table S5: Table of neutral losses of neutral losses of compounds **6**-**9**

Neutral losses of compounds **6**-**9**
H_2O, C_3H_6, CO, C_4H_6, C_3H_4, CO_2, CH_4, C_2H_4, CH_3, C_2H_2, CH_4O, H_2, CH_2O_2, C_2O_2, C_3O, C_4H_4, C_4H_8, CH_5O, C_3H_8, C_3H_7, CH_4O_2, C_5H_8, C_3H_2O, C_5O, C_6H_{12}, C_2H_6O, $C_4H_4O_2$, C_5H_4, $C_5H_2O_2$, C_2H_2O

Table S6: Table of fragmented ions of compound **1**.

Fragmented ions	Neutral losses
$C_{18}H_{22}O_4$, $C_{18}H_{20}O_3$, $C_{10}H_{10}O_2$, $C_8H_8O_2$, $C_7H_6O_2$, C_8H_8O, C_9H_6, $C_6H_6O_2$, C_7H_6O, C_8H_8, C_8H_6, C_7H_{10}, C_6H_6O, C_7H_8, C_7H_6, C_6H_8, C_6H_6, C_6H_4, C_5H_8, C_4H_4O, C_3O_2, C_5H_6, C_5H_4, C_4H_8, C_4H_6, C_3H_2O, C_4H_4, C_3O	H_2O, C_2H_2, CO, CH_4, C_2H_2O, CH_4O_2, $C_4H_4O_2$, CH_3, C_2H_4, C_3H_2O, C_4H_4
Percentage of agreement	Percentage of agreement
23.1%	36.7%

Table S7: Table of fragmented ions of compound **2**.

Fragmented ions	Neutral losses
$C_{18}H_{22}O_4$, $C_{14}H_{16}O_4$, $C_{12}H_{14}O_4$, $C_9H_6O_3$, $C_8H_6O_3$, $C_8H_8O_2$, $C_7H_6O_2$, C_8H_8O, $C_7H_4O_2$, C_9H_{10}, $C_6H_6O_2$, C_8H_8, C_7H_{10}, C_6H_6O, C_7H_8, C_7H_6, C_6H_8, C_6H_6, C_6H_4, C_5H_8, C_4H_4O, C_3O_2, C_5H_6, C_5H_4, C_4H_6, C_3H_2O, C_4H_4, C_3O	H_2O, C_4H_6, C_2H_2, CH_4, C_3H_4, C_2H_2O, CO, $C_4H_4O_2$, C_3H_6, C_2H_4, C_4H_4, C_2O_2, C_3H_2O, CH_3
Percentage of agreement	Percentage of agreement
23.1%	46.7%

Table S8: Table of fragmented ions of compound **3**.

Fragmented ions	Neutral losses
$C_{18}H_{24}O_4$, $C_7H_6O_2$, C_8H_8O, C_9H_{10}, $C_6H_6O_2$, C_8H_8, C_7H_{10}, C_6H_6O, C_7H_8, C_7H_6, C_6H_8, C_6H_6, C_6H_4, C_5H_8, C_4H_4O, C_3O_2, C_5H_6, C_5H_4, C_4H_8, C_4H_6, C_3H_2O, C_4H_4, C_3O	H_2O, C_4H_6, C_2H_2, CH_4, C_2H_2O, CO, C_4H_8, C_2O_2, C_2H_4, C_4H_4, $C_4H_4O_2$, CH_3, C_3H_2O
percentage of agreement	percentage of agreement
19.0%	43.3%

Table S9: Table of fragmented ions of compound **4**.

Fragmented ions	Neutral losses
$C_{18}H_{26}O_4$, $C_6H_6O_2$, C_8H_{10}, C_8H_8, C_7H_{10}, C_6H_6O, C_7H_8, C_7H_6, C_6H_8, C_6H_6, C_6H_4, C_5H_8, C_4H_4O, C_3O_2, C_5H_6, C_5H_4, C_4H_8, C_4H_6, C_3H_2O, C_4H_4, C_3O	H_2O, C_2H_2O, C_4H_8, C_2H_2, CH_4, CO, C_2H_4, H_2, C_4H_4, C_3H_2O
Percentage of agreement	Percentage of agreement
17.3%	33.3%

Table S10: Table of fragmented ions of compound **5**.

Fragmented ions	Neutral losses
$C_{14}H_{14}O_2$, $C_{14}H_{12}O_2$, $C_{13}H_8O_2$, $C_{13}H_8O$, $C_9H_6O_4$, $C_{11}H_8O_2$, $C_8H_6O_4$, $C_{13}H_8$, $C_{11}H_8O$, $C_{12}H_8$, $C_{12}H_7$, $C_8H_6O_3$, $C_{10}H_8O$, $C_{11}H_8$, $C_{10}H_{10}$, C_9H_6O, $C_{10}H_8$, $C_{10}H_7$, $C_7H_4O_2$, C_9H_8, C_9H_6, C_8H_6, C_6H_6O, C_7H_6, C_5H_6, C_5H_4, C_3H_2O	H_2O, CO, C_3H_6, H_2, CH_3, C_2H_2, C_2O_2, C_2H_2O, C_3H_2O
Percentage of agreement	Percentage of agreement
22.3%	30.0%
